# Black bean pod-derived pyrochar for single and binary adsorption of Cu(ii) and direct red 79: adsorption performance, interfacial interactions and regeneration

**DOI:** 10.1039/d6ra07689k

**Published:** 2026-10-29

**Authors:** Ngoc Anh Hoang, Thi Thuy Ha Dinh, Truong Xuan Vuong, Tien Duc Pham, Thuong Tuan Nguyen, Thi Thao Truong

**Affiliations:** a TNU-University of Sciences, Thai Nguyen University Phan Dinh Phung Ward Thai Nguyen 250000 Vietnam thao.truong671@gmail.com thao.tt@tnus.edu.vn; b Faculty of Chemistry, University of Science, Vietnam National University Hanoi, 19 Le Thanh Tong, Cua Nam Hanoi 100000 Vietnam; c Institute of Life Sciences, Thai Nguyen University Phan Dinh Phung Ward Thai Nguyen 250000 Vietnam

## Abstract

The interplay between interfacial interactions and pore accessibility during binary adsorption of co-existing metal ions and anionic dyes on biomass-derived pyrochars remains insufficiently understood. Here, a pyrochar derived from black bean pods was investigated for Cu(ii) and Direct Red 79 (DR79) adsorption in single-solute and binary systems. Controlled pyrolysis (300–700 °C, 1–5 h) progressively altered the carbon structure and pore characteristics of the resulting pyrochars. BP7003, prepared at 700 °C for 3 h, exhibited a specific surface area of 517 m^2^ g^−1^, a substantial microporous contribution accompanied by mesopore-range features, and oxygen-containing surface functionalities. Cu(ii) showed rapid uptake, reaching a maximum experimentally determined equilibrium adsorption capacity of 117.13 ± 0.95 mg g^−1^ at pH 5 under the investigated conditions. Its adsorption behaviour was consistent with contributions from surface electrostatics and oxygen-containing surface functionalities. DR79 exhibited a lower experimental equilibrium adsorption capacity of 66.05 ± 1.15 mg g^−1^, with its adsorption behaviour suggesting a stronger influence of pore accessibility within the heterogeneous carbon structure. Kinetic and equilibrium analyses were consistent with heterogeneous adsorption environments for both solutes. Binary adsorption exhibited concentration-dependent behaviour under the investigated binary conditions, with small deviations from the corresponding single-solute reference systems across the tested concentration combinations. These observations are compatible with changes in the interfacial environment and competition for accessible adsorption domains, although a specific Cu-associated interaction mechanism remains unconfirmed. BP7003 retained most of its adsorption performance after four regeneration cycles, although a greater decline was observed for DR79. Overall, the results highlight the combined roles of surface chemistry, interfacial electrostatics, and pore accessibility in shaping Cu(ii)–DR79 co-adsorption across different loading regimes.

## Abstract 2

Pyrochar (BP7003) simultaneously removes Cu_2+_ and DR79 *via* distinct yet interacting mechanisms, with synergy at concentrations and competition at higher loadings.

## Graphical abstract

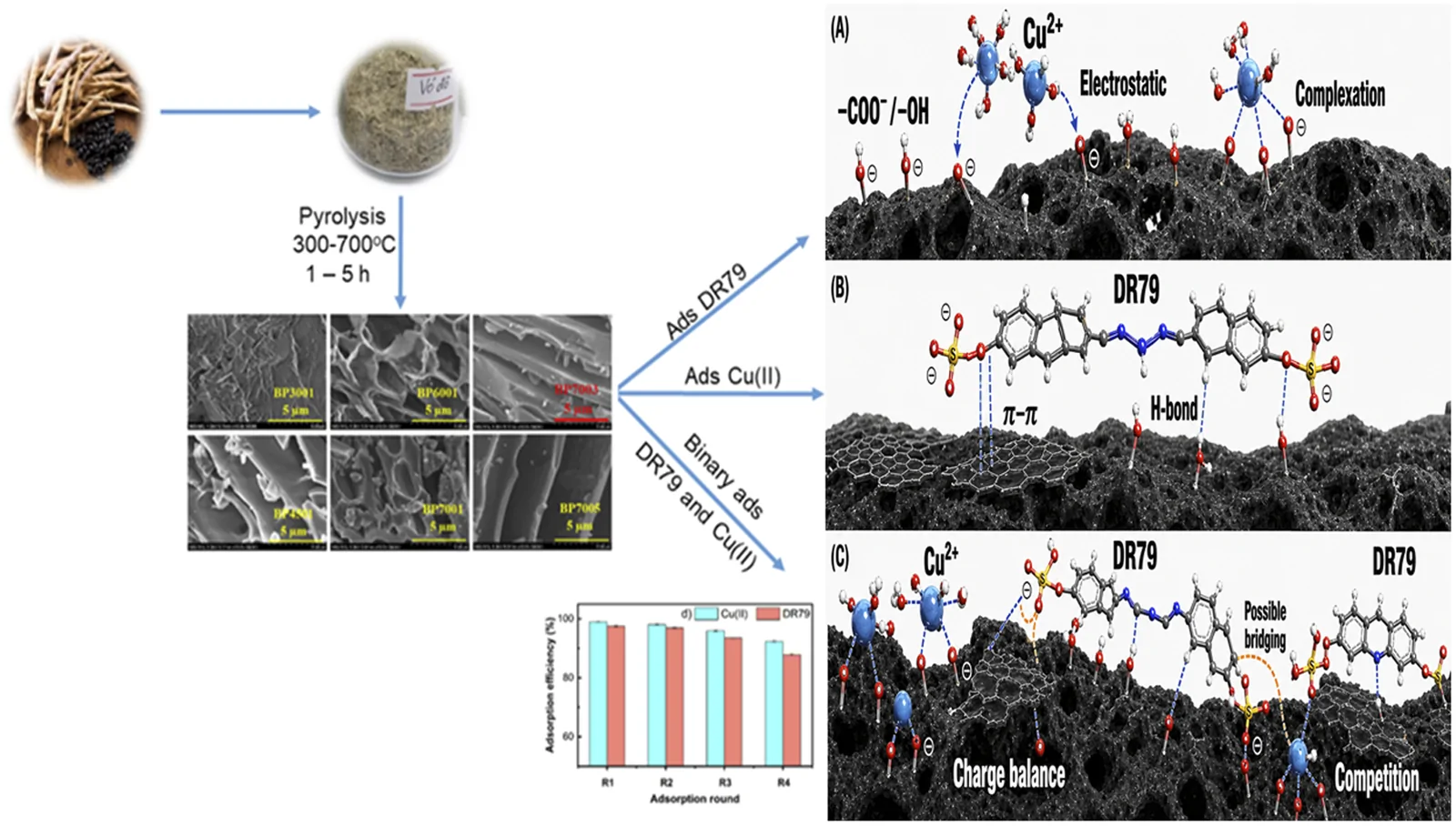

## 1. Introduction

Water pollution is increasingly characterized by the occurrence of multiple contaminants rather than isolated pollutants. In industrially impacted waters, inorganic ions and organic compounds can coexist and interact, complicating contaminant removal and the interpretation of adsorption behaviour. Among inorganic contaminants, Cu(ii) is associated with electroplating, textile dyeing, mining, metal-finishing, and related industrial activities,^1^ particularly in textile and dyeing industries where large volumes of contaminated wastewater are generated.^2^ Although copper is an essential micronutrient at trace levels, elevated concentrations can exert toxic effects on aquatic organisms and disturb ecosystem processes. Effective removal of dissolved Cu(ii) therefore remains an important challenge in water treatment.

Azo dyes are persistent aquatic contaminants because their chemically stable structures can hinder conventional degradation processes. Direct Red 79 (DR79), an anionic azo dye containing extended aromatic/conjugated structures and sulfonate groups, represents a comparatively demanding adsorbate because its molecular dimensions and distributed charge centres can influence interfacial interactions and access to porous structures.^3^ Compared with extensively studied dyes such as methylene blue, Rhodamine B, Congo Red, and Crystal Violet,^4^ structurally complex dyes such as DR79 have received comparatively less attention. This is particularly relevant for large anionic dyes, for which molecular dimensions and distributed charge centres may impose accessibility constraints that are not readily captured by single-solute adsorption experiments.

Textile effluents commonly contain mixtures of dyes and inorganic contaminants rather than isolated pollutants.^2,4^ Heavy metals such as Cu, Cr, Ni, and Zn may enter these waste streams through dyeing auxiliaries, metal-containing formulations, catalysts, and equipment corrosion during industrial processing.^5^ Consequently, treatment systems may need to remove both dyes and metal ions within the same treatment process.^4,6^ Treatment strategies developed for individual pollutants often lose efficiency under such conditions. Biological processes are often inhibited by the combined toxicity of heavy metals and dyes, which impair microbial metabolism. Coagulation–flocculation generates secondary sludge.^7^ Photocatalytic and electrochemical systems increase operational complexity and cost.^8–10^

Recent advances in membrane-based separation have shown that interfacial architecture, nanofiller incorporation and polymer-network regulation can modulate selective solute transport, water permeance and the permeability–selectivity trade-off in aqueous separation.^11–14^ These developments underscore the role of interfacial chemistry and transport pathways in selective separation. In parallel, adsorption using biomass-derived carbons remains an attractive approach for dissolved-contaminant removal because of the availability of low-cost precursors and the tunability of surface chemistry, pore structure and surface–solute interactions.

Adsorption is particularly attractive for such mixed-contaminant systems because carbonaceous materials can accommodate chemically diverse pollutants through electrostatic, surface-chemical, and pore-accessibility-related interactions.^15–17^ However, coexisting solutes need not interact independently: they can compete for adsorption domains, modify local interfacial environments, or alter the accessibility of favourable sites. Previous studies involving Cu(ii) with antibiotics or dyes have demonstrated condition-dependent co-adsorption behaviour.^18–20^ In contrast, binary adsorption of Cu(ii) with DR79 has received limited systematic investigation, and the interfacial and pore-accessibility factors influencing their non-independent behaviour remain poorly constrained. This gap is particularly relevant to large anionic dyes, for which molecular dimensions and distributed charge centres may introduce accessibility effects that are not evident from the corresponding single-solute systems.

Biochar is commonly used as a general term for carbonaceous materials derived from biomass through thermochemical conversion, whereas pyrochar specifically refers to char produced by pyrolysis under oxygen-limited conditions.^21^ In the present study, the term “pyrochar” is used consistently to refer to the carbonaceous materials produced from black bean pods by controlled pyrolysis. The physicochemical properties of pyrochar are strongly influenced by processing parameters such as temperature, heating rate, and residence time, which affect carbonization, pore development, mineral redistribution, and surface functionality.^21,22^ These transformations can produce heterogeneous carbon-rich surfaces containing residual oxygen-bearing functionalities capable of interacting with both metal ions and organic molecules.^22^ Compared with conventional activated carbon, biomass-derived chars also offer renewable feedstocks, potentially lower production costs, and opportunities for physicochemical tailoring.^23^

Agricultural residues are attractive precursors for pyrochar production because of their availability, low cost, and renewable nature. Legume-derived wastes, including pods and shells, have been investigated as precursors for carbonaceous adsorbents because their lignocellulosic composition provides a suitable carbon source and can promote pore development during pyrolysis.^24–26^ Previous studies have demonstrated the adsorption potential of bean-, soybean-, peanut-, and kidney-bean-derived carbonaceous materials for metals,^27,28^ pharmaceuticals, chlorinated compounds, and other aqueous contaminants.^29–34^ However, these studies have largely focused on single-solute applications or individual aspects of material optimization. Moreover, most investigations have emphasized pyrolysis temperature or post-synthesis modification as the principal routes for improving adsorption performance,^35^ whereas residence time has received comparatively less systematic attention despite its potential influence on devolatilization, pore development, carbon condensation, and mineral redistribution.^21,35^ Consequently, the contribution of residence time to pyrochar evolution and subsequent adsorption behaviour remains less well constrained than that of pyrolysis temperature.

Black bean pods provide a relevant but comparatively underexplored precursor in this context. Legumes such as peanuts, soybeans, and black beans are widely cultivated in Vietnam, generating agricultural residues.^36^ Black bean pods therefore represent a potentially renewable biomass resource for producing carbonaceous adsorbents.^25^ Their lignocellulosic composition provides a suitable carbon-rich precursor for pyrochar production and can promote the development of porous structures during thermal conversion.^37^ Previous studies have extensively investigated peanut- and soybean-derived biochars for adsorption applications,^31,38^ whereas black bean pod-derived pyrochars have received comparatively limited attention. This makes black bean pod-derived pyrochar a relevant platform for examining the binary adsorption of a metal ion and an anionic azo dye under controlled pyrolysis conditions.

Previous studies have established that biomass-derived materials can simultaneously remove metal ions and dyes, but how physicochemical changes in these materials influence non-independent adsorption remains incompletely understood. For example, dried sugar beet pulp was investigated for binary biosorption of Cu(ii) and an anionic reactive dye,^39^ while pea-shell-derived biochar has also been evaluated for simultaneous removal of Cu(ii) and malachite green.^40^ Studies using carbonaceous materials have further shown that Cu(ii) can alter dye uptake, with the magnitude and direction of the effect depending on the adsorbate system and experimental conditions.^18,19,41^ These findings indicate that binary metal–dye adsorption cannot always be inferred from single-solute behaviour alone. However, the relationship between the thermal evolution of a biomass-derived carbon surface and concentration-dependent metal–dye interactions remains insufficiently constrained.

The present study addresses this gap using black-bean-pod-derived pyrochars prepared over a controlled range of pyrolysis temperatures (300–700 °C) and residence times (1–5 h). Rather than evaluating binary removal using a single fixed adsorbent, we examine how changes in carbon structure, surface chemistry and textural properties are reflected in the adsorption of Cu(ii) and Direct Red 79 (DR79). DR79 was selected as a relatively large anionic azo dye containing multiple sulfonate groups, providing a chemically and sterically distinct counterpart to Cu(ii). Single-solute and binary adsorption were further compared under different loading conditions to determine whether the nature of the Cu(ii)–DR79 interaction changed with loading.

The principal contribution of this work is therefore not the demonstration of simultaneous metal–dye removal itself, but the identification of loading-dependent, non-independent adsorption behaviour in the Cu(ii)–DR79 system and its relationship to the thermally evolved physicochemical properties of the biomass-derived carbon. The combined adsorption, spectroscopic, electrokinetic and textural evidence is used to distinguish experimentally supported surface effects from plausible but unresolved interfacial interactions.

## 2. Materials and methods

### 2.1. Chemicals

The Cu(ii) standard solution (1000 mg L^−1^) for atomic absorption spectroscopy (AAS) (Sigma-Aldrich), Direct Red 79 (DR79, C_37_H_28_N_6_Na_4_O_17_S_4_, MedChem Express), hydrochloric acid (HCl, 36–37%, Isochem), and sodium hydroxide (NaOH, 98.5%, Isochem) were used in this study. Deionized water was used for all solution preparations. Black bean pods were collected from Phu Binh district, Thai Nguyen province, Vietnam.

All chemicals were of analytical grade and used without further purification.

### 2.2. Synthesis of bean pod pyrochar

The black bean pods were washed thoroughly with deionized water, oven-dried, and ground using a medicinal herb grinder. For each pyrolysis condition, 10.00 g of biomass was used for each independent preparation. Pyrochars were prepared at 300, 450, 600, and 700 °C with the residence times specified in Table S1 (SI). After pyrolysis, the products were dried and mechanically re-ground prior to adsorption experiments. The samples prepared at 700 °C for 1, 3, and 5 h were designated BP7001, BP7003, and BP7005, respectively. The pyrolysis process was conducted in a tubular furnace under a continuous nitrogen flow (120 mL min^−1^) at a heating rate of 3 °C min^−1^.

After pyrolysis, the samples were allowed to cool naturally to room temperature under nitrogen atmosphere. The obtained pyrochar was washed with 0.1 M HCl to remove soluble inorganic species, followed by repeated washing with deionized water until constant conductivity was reached.^21^ The material was then dried, ground, weighed (*m*_f_) and stored in a desiccator. The mass yield (MS) is calculated by equation:1
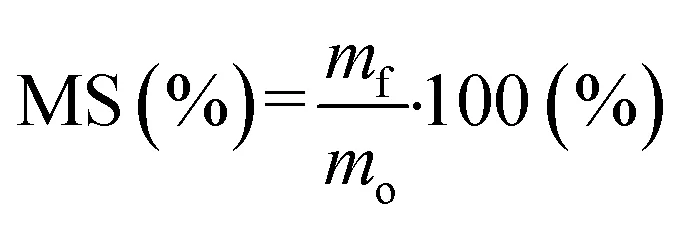


The samples were denoted as BPTt, where *T* represents the pyrolysis temperature (°C) and *t* represents the residence time (h), *e.g.*, BP7003. The preparation process of the pyrochar samples is illustrated in detail in the schematic diagram in Fig. 1 as follows:

**Fig. 1:**
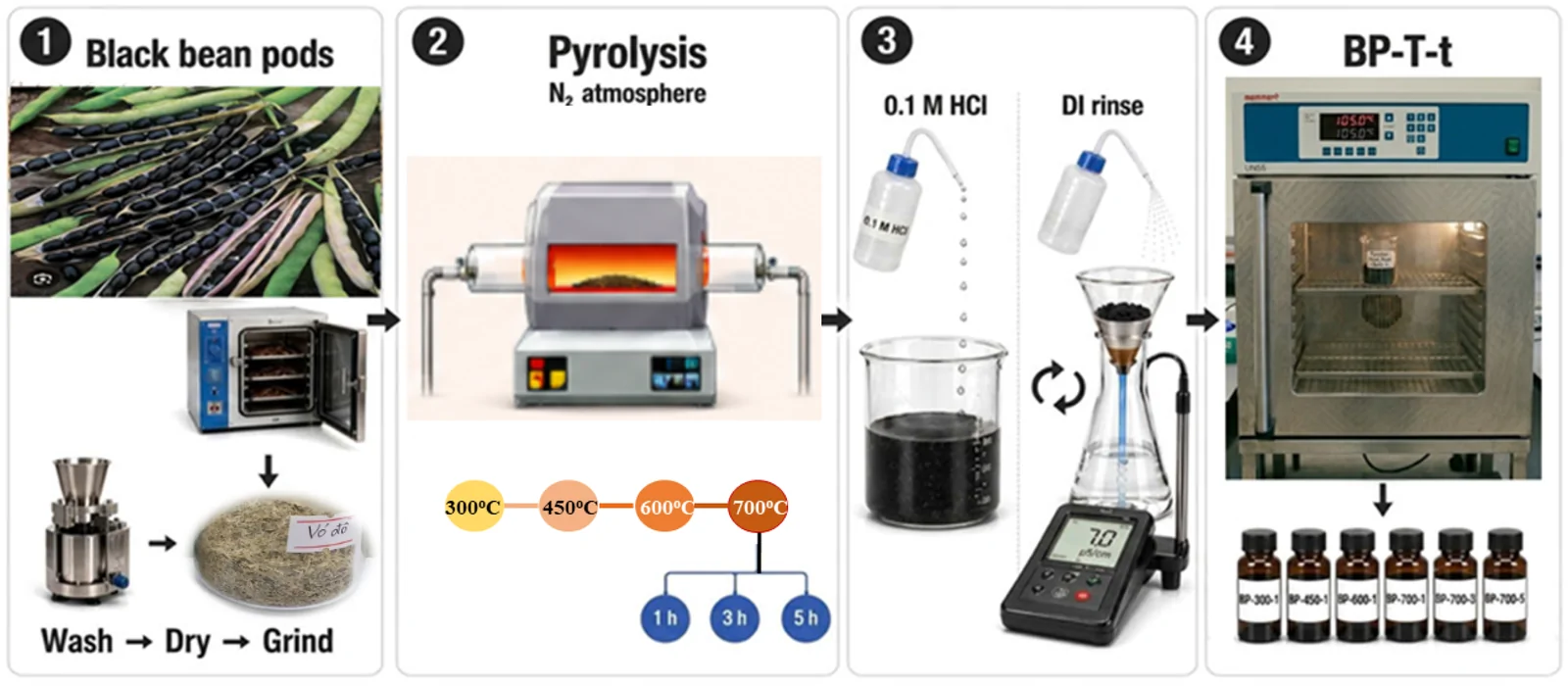
Schematic illustration of the preparation of black bean pod-derived pyrochars (BPTt): (1) pretreatment of black bean pods; (2) N_2_-atmosphere pyrolysis; (3) post-pyrolysis washing; (4) drying, grinding, and storing samples.

### 2.3. Material characterization techniques

BP samples were characterized using several physicochemical techniques: X-ray diffraction (XRD, PANalytical X'Pert PRO MPD diffractometer, Cu Kα radiation, *λ* = 0.15406 nm, 2*θ* range of 14–60°); Raman spectroscopy (LabRAM HR Evolution, Horiba, 532 nm excitation); Fourier-transform infrared spectroscopy (FTIR, Spectrum Two, PerkinElmer, USA, 4000–400 cm^−1^); scanning electron microscopy coupled with energy-dispersive X-ray spectroscopy (SEM-EDS, Hitachi S-4800/JEOL 6490–JED-2300); Brunauer–Emmett–Teller (BET) analysis (TriStar 3000 V6.07 A, liquid nitrogen at 77 K, degassing at 200 °C); zeta-potential measurements (Zetasizer Nano ZS, Malvern Instruments) in 0.1 M NaCl at pH 4 and 25 °C; and X-ray photoelectron spectroscopy (XPS, Nexsa G2, Al Kα radiation, 1486.6 eV). The XPS binding energies were calibrated using the C 1s peak at 284.8 eV. High-resolution spectra were fitted using Gaussian functions with a Shirley background.

### 2.4. Adsorption: experiment and calculation

#### 2.4.1. Analytical determination of DR79 and Cu(ii)

The residual concentration (*C*_t_) of DR79 was determined by UV-Vis spectrophotometry (Agilent Cary 60), whereas Cu(ii) was quantified by atomic absorption spectrometry (AAS, Shimadzu 6300, Japan) at 324.8 nm. Each filtrate was measured in triplicate, and the reported concentration was calculated as the mean of the three instrumental measurements. The corresponding calibration curves are provided in Fig. S1 (SI), and the analytical characteristics are summarized in Table S2 (SI).

For DR79, quantitative determination was performed using calibration standards over 0–30 mg L^−1^ at pH 2 and pH 4, with the absorbance measured at 501 nm. The calibration responses showed excellent linearity, with *R*^2^ = 0.9982 and 0.9987, respectively. For mixed solutions containing DR79 and Cu(ii) in different electrolyte media, the wavelength of maximum absorption for DR79 shifted from approximately 501 nm to 496 nm. Accordingly, two calibration curves for the determination of DR79 in mixed solutions—one for an aqueous medium and one for a medium containing 0.1 M K_2_SO_4_ at pH 4—were established within the concentration range of 0 to 20 mg L^−1^. The slope, intercept, *R*^2^, blank standard deviation, LOD, and LOQ for the investigated DR79 calibration conditions are summarized in Table S2 (SI).

For Cu(ii), quantitative determination was performed using an external calibration curve over 0.1–10 mg L^−1^ at 324.8 nm. Calibration standards were prepared from a 1000 mg L^−1^ Cu standard solution (Sigma-Aldrich). The calibration response showed excellent linearity over the established concentration range (*R*^2^ = 0.9998). The corresponding calibration curve and analytical characteristics are provided in Fig. S1b and Table S2 (SI), respectively.

LOD and LOQ were calculated from the calibration slope and the standard deviation of six blank measurements according to:

2 LOD = 3.3*σ*/*S*

3 LOQ = 10*σ*/*S*

where *σ* is the standard deviation of the six blank measurements and *S* is the calibration slope. The resulting LOD and LOQ values are summarized in Table S2 (SI).

Samples with concentrations exceeding the applicable calibration ranges were appropriately diluted prior to measurement. The dilution factors applied to the initial and post-adsorption samples are summarized in Table S3 (SI), and the final concentrations obtained after dilution were maintained within the corresponding established calibration ranges.

The experiments were conducted as follows. First, the adsorption ability of 20 mg per L DR79 was evaluated using 1 g L^−1^ of the prepared materials (BP3001, BP4501, BP6001, BP7001, BP7003, and BP7005) under defined conditions (pH 4, 25 °C, 120 min), and the optimal adsorbent was selected based on the highest adsorption efficiency (AE). Subsequently, the independent adsorption of DR79 and Cu(ii) by the optimal material was investigated at different pH values (2–6 for DR79 and 2–5 for Cu(ii)), adsorbent dosages (0.5–5.0 g L^−1^ for DR79 and 0.5–5.0 g L^−1^ for Cu(ii)), contact times (5–180 min), and initial adsorbate concentrations (10–500 mg L^−1^ for DR79 and 10–400 mg L^−1^ for Cu(ii)). Based on these results, pH 4 and a Cu(ii)–DR79 concentration combination of 20 and 10 mg L^−1^, respectively, were selected for the binary-system investigation.

#### 2.4.2. Adsorption isotherm models

The experimental data were fitted to five adsorption isotherm models, namely Langmuir, Freundlich, Temkin, Redlich–Peterson, and Sips models, using the following equations.^42,43^

Langmuir equation: 4
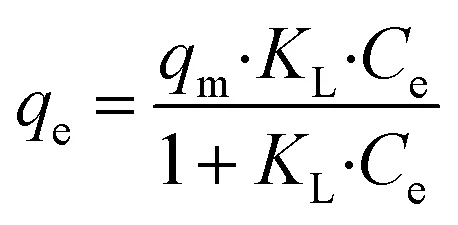


Freundlich equation:

5 *q*_e_ = *K*_F_·*C*_e_^1/*n*_F_^

Temkin equation:

6 *q*_e_ = *B*_T_·ln(*K*_T_·*C*_e_)

Redlich–Peterson equation:7
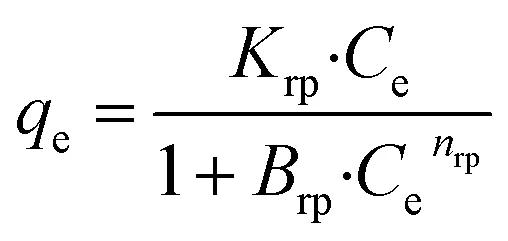


Sips equation: 8
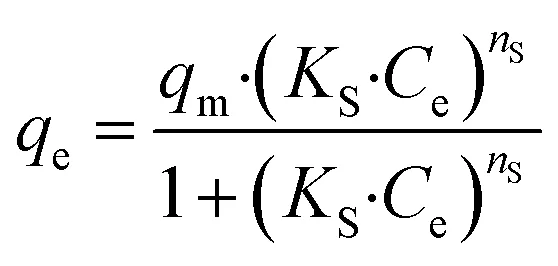
where *q*_e_ and *q*_m_ are the equilibrium adsorption capacity and the maximum adsorption capacity (mg g^−1^), respectively. The maximum experimentally determined equilibrium adsorption capacity is denoted as *q*_e,exp_, whereas *q*_m,cal_ denotes the model-derived maximum adsorption capacity calculated from the corresponding isotherm model. *K*_L_ (L mg^−1^) is the Langmuir constant; *K* (mg^1−1/*n*_F_^ L^1/*n*_F_^ g^−1^) and *n*_F_ are the Freundlich constant and dimensionless exponent, respectively; *K*_*T*_ (L mg^−1^) and *B*_*T*_ (mg g^−1^) are the Temkin constants; *K*_rp_ (L g^−1^), *B*_rp_ ((L mg^−1^)^*n*_rp_^), and *n*_rp_ are the Redlich–Peterson constants, with *n*_rp_ dimensionless; and *K*_s_ (L mg^−1^) and *n*_s_ are the Sips constant and dimensionless exponent, respectively. *R* is the gas constant, and *T* is the temperature.

#### 2.4.3. Adsorption kinetic models

The adsorption kinetics were fitted to curve equations of several models: pseudo-first-order (PFO), pseudo-second-order (PSO), intra-particle diffusion (ID), and Elovich kinetic models:^37^

PFO kinetic model:

9 *q*_*t*_ = *q*_e_(1 − e^−*k*_1_*t*^)

PSO kinetic model: 10
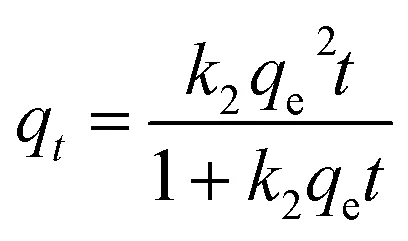


ID kinetic model:

11 *q*_*t*_ = *k*_d_*t*^0.5^ + *C*_ID_

Elovich kinetic model: 12
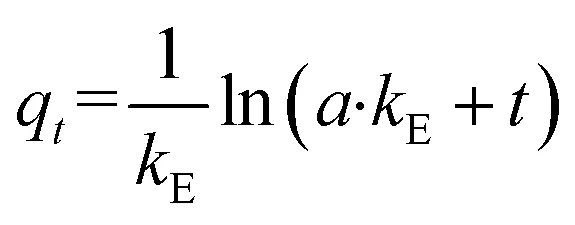
where *k*_1_ (min^−1^), *k*_2_ (g mg^−1^ min^−1^) and *k*_d_ (mg g^−1^ min^−0.5^) are the rate constants corresponding to the PFO, PSO, and ID kinetic model, respectively; and *k*_E_ (g mg^−1^) is the Elovich desorption constant related to surface coverage and activation energy; *C*_ID_ is a parameter in the diffusion kinetics equation, and a is the initial adsorption rate (mg g^−1^ min^−1^).

The agreement between the experimental results and the above models was evaluated through the correlation coefficient (*R*^2^) and root mean square error (RMSE) values. A higher *R*^2^ value combined with a lower RMSE value indicated better agreement between the experimental and calculated data.

### 2.5. Reusability of material

The reusability of the adsorbent was evaluated through adsorption–desorption cycles in the Cu(ii)–DR79 single and binary system (20 and 10 mg L^−1^, respectively) at pH 4. After each adsorption experiment, the used adsorbent was separated by centrifugation and regenerated by sequential desorption. A portion of the material recovered after the first adsorption cycle (prior to reuse) was gently washed with distilled water (confirming the absence of Cu(ii) and DR79 in the washings), dried, and subjected to post-adsorption analyses (zeta potential, FTIR, XPS). The material used for single-adsorbate studies (Cu(ii) or DR79) was processed under identical conditions and procedures. The remaining portion was reserved for reuse.

To reuse, first, the material was treated with 70% ethanol and shaken for 120 min, centrifuged, and the supernatant collected for UV-Vis analysis. This process was repeated until the DR79 concentration in the wash solution was below the applicable detection limit. Next, the solid was treated by soaking in 0.1 M HCl for 15 min; the Cu(ii) concentration in the wash solution was checked by AAS. This process was repeated until the Cu(ii) concentration was below the AAS detection limit. The regeneration endpoint was therefore defined analytically rather than by a predetermined number of washing steps. The cumulative eluent volume and number of repeated washing steps were not recorded because they depended on the residual adsorbate loading and the corresponding analytical endpoint. Finally, the adsorbent was washed with deionized water until the pH was neutral, dried at 60 °C, and reused in the next adsorption cycle.

### 2.6. Statistics

All adsorption experiments were conducted as three independent adsorption experiments (*n* = 3), and results are reported as mean ± standard deviation (SD). For the binary adsorption comparison, one-way ANOVA was performed separately for each matched concentration set, followed by Tukey's multiple-comparison test. Statistical significance was evaluated at *p* < 0.05 using Tukey-adjusted pairwise *p*-values.

Nonlinear regression was applied to fit the adsorption isotherms (Langmuir, Freundlich, Temkin, Redlich–Peterson, and Sips) and kinetic models (PFO, PSO, intra-particle diffusion, and Elovich). The fitting procedure was carried out using OriginPro 2019 (OriginLab Corporation, USA). Model parameters were estimated by nonlinear least-squares fitting by minimizing the sum of squared residuals (SSE) between the experimental and model-calculated values. Model performance was assessed using *R*^2^ and RMSE, while fitted parameters are reported with their associated parameter uncertainties. Statistical data processing and graphical visualization were performed using RStudio with the ggplot2 package.

Model performance was primarily assessed using the coefficient of determination (*R*^2^). In addition, the consistency between experimental and calculated adsorption capacities (*q*_e_,_exp_*vs. q*_e_,_cal_) was considered during model selection to avoid overinterpretation based solely on *R*^2^.

For kinetic analysis, the agreement between experimental data and model predictions was evaluated across the entire time range rather than at isolated points, ensuring a more reliable interpretation of adsorption behavior.

## 3. Results and discussion

### 3.1. Characterization

The mass yields are summarized in Table S1 (SI). Experimental results indicate that the yield was strongly dependent on the pyrolysis temperature: as the temperature increased from 300 °C to 700 °C at a fixed residence time of 1 h, the yield decreased from 28.40% to 24.64%. However, extending the residence time at 700 °C had a negligible effect on the yield; increasing the residence time from 1 to 5 h resulted in only a minor decrease in the yield from 24.64% to 23.98%. This behaviour reflects progressive devolatilization and carbonization during pyrolysis, resulting in volatile loss and enrichment of the carbonaceous fraction. Nevertheless, the pyrochar yield obtained from black bean pods in this study was generally lower than those reported for similar agricultural residues, such as peanut shells and soybean hulls.^44,45^ This discrepancy could be attributed to the loss of soluble ash fractions and loosely bound fine particles during the post-pyrolysis washing process.

The physicochemical properties of the prepared pyrochars were investigated using complementary structural, spectroscopic, morphological, and textural characterization techniques.

#### 3.1.1. XRD analysis

The structural evolution of the black bean pod precursor (BP) and its derived pyrochars (BPTt) under different synthesis conditions was examined using XRD together with complementary techniques (Raman, FTIR, EDS, SEM, and BET).

As shown in Fig. 2a, the raw biomass displays a sharp diffraction peak at 2*θ* ≈ 22°, which is characteristic of crystalline cellulose I.^46^ This feature disappears after thermal treatment, reflecting the breakdown of the native lignocellulosic framework.

**Fig. 2:**
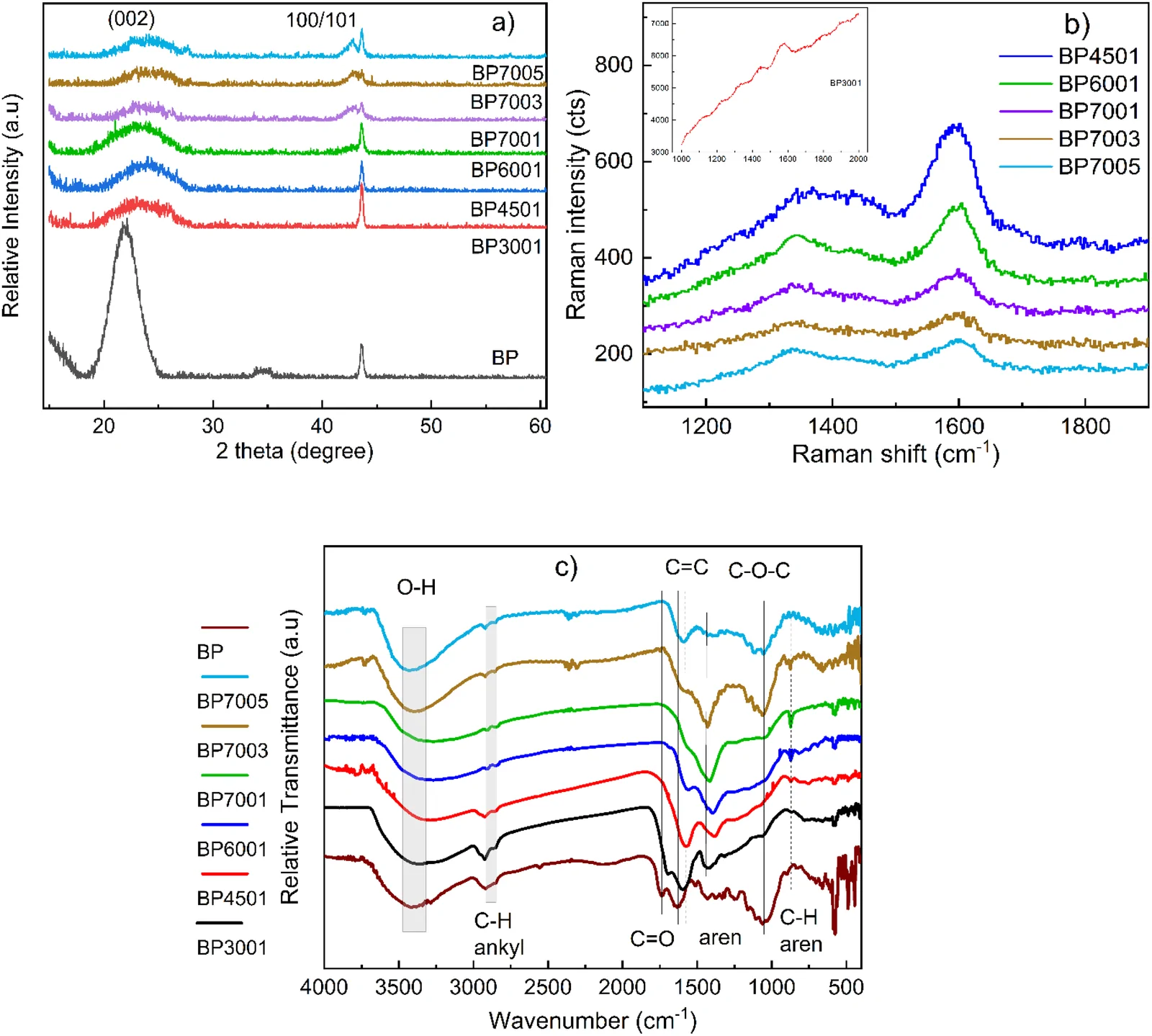
Characterization of BP and BPTt samples: (a) XRD patterns, (b) Raman spectra, and (c) FTIR spectra.

At lower pyrolysis temperatures (300–450 °C, 1 h; BP3001 and BP4501), a broad band emerges in the 23–26° range. This band corresponds to the (002) reflection of disordered carbon structures associated with turbostratic stacking of aromatic layers. The absence of sharp peaks in this region suggests that carbonization is still incomplete and long-range ordering has not yet developed. Raising the temperature to 600–700 °C alters the diffraction pattern. The (002) band becomes more distinct, and a weak feature appears near 43°, attributed to the (100)/(101) reflections of in-plane ordering within aromatic carbon domains. Prolonging the residence time at 700 °C further promotes secondary carbonization and structural rearrangement. For 1–2 h, the broad (002) band reflects small aromatic layers with poor stacking order. When extended to 3–5 h, the band narrows and the background flattens, suggesting growth of aromatic sheets and strengthened π–π stacking. Simultaneously, the feature at 42.6–43.6° becomes more intense and better resolved, consistent with increased ordering within the carbonaceous domains. The improved peak definition is consistent with increased structural ordering within the carbonaceous domains. Overall, the XRD results indicate a progressive increase in the apparent structural ordering of the carbonaceous domains with increasing pyrolysis temperature and residence time. The development of the broad (002)-associated feature and the feature near 43° is consistent with increasing structural ordering within the carbonaceous domains, particularly for the samples treated at 700 °C for longer residence times. However, the broad and relatively diffuse nature of these reflections does not provide evidence of well-developed long-range graphitic order. Therefore, the XRD data are interpreted as evidence of progressive structural rearrangement and increased ordering within the carbonaceous domains, rather than as direct evidence of graphitization. The relationship between these structural characteristics and adsorption behaviour is considered together with the complementary Raman, FTIR, surface-area, and adsorption results.

#### 3.1.2. Raman analysis

Raman spectroscopy provides further insight into structural rearrangements during pyrolysis (Fig. 2b, and Table 1). The spectra reveal the characteristic D (1331–1360 cm^−1^), D″ (1427–1480 cm^−1^), and G (1570–1593 cm^−1^) bands across all samples, indicating coexistence of disordered and graphitic sp^2^ carbon domains.

**Table 1:** The Raman parameters of BP samples

Sample	BP3001	BP4501	BP6001	BP7001	BP7003	BP7005
*ν*_D_ (cm^−1^)	1331	1360.7	1343	1344	1340.8	1339
*I*_D_	153.40	133.20	93.70	63.10	47.20	39.40
*ν*_D″_ (cm^−1^)	1446.0	1456.5	1451.0	1451.0	1460.1	1427.6
*I*_D″_	224.70	63.15	56.25	19.97	22.43	12.80
*ν*_G_ (cm^−1^)	1570.0	1589.4	1591.3	1589.5	1590.2	1592.6
*I*_G_	492.49	258.11	153.97	82.23	59.88	50.14
*I*_D_/*I*_G_	0.31	0.52	0.61	0.77	0.79	0.79
*I*_D″_/*I*_G_	0.31	0.18	0.27	0.23	0.25	0.21

With increasing temperature and residence time, the overall Raman intensity declines. At the same time, the D″ band weakens markedly. This band is commonly associated with amorphous or aliphatic carbon, particularly CH_2_-related structures. Its attenuation suggests progressive removal of thermally unstable components originating from cellulose and hemicellulose.

Meanwhile, the D and G bands become more distinct. This shift reflects condensation of smaller aromatic fragments into larger sp^2^ clusters. The fluorescence background also decreases at higher temperatures, consistent with the loss of oxygenated and conjugated surface species that contribute to background scattering under visible excitation. As carbonization proceeds, these species transform into more condensed aromatic domains, in line with the XRD observations.^47,48^

The Raman spectrum was peak-fitted using a Gaussian function to determine the positions and intensities of the spectral peaks (Fig. S2 (SI) and Table 1). The *I*_D_/*I*_G_ ratio increases from 0.31 (300 °C) to 0.77 (700 °C) and then stabilizes (0.77–0.79) with extended treatment at 700 °C. This trend does not point to graphitic ordering. Instead, it reflects the formation of defect-rich aromatic structures and small sp^2^ domains during carbonization.^49,50^ The minimal change between BP7003 and BP7005 suggests that structural rearrangement approaches a steady state under prolonged heating, a pattern also reported for other biomass-derived carbons.^51^

The D″ band, linked to CH_2_ bending vibrations in less ordered phases,^51^ remains relatively stable in normalized form (*I*_D″_/*I*_G_ ≈ 0.18–0.25, except BP3001). Most labile aliphatic components have already been removed by this stage, leaving a matrix dominated by aromatic domains containing structural defects. Among the high-temperature samples, BP7003 shows the highest *I*_D″_/*I*_G_ ratio, pointing to a higher density of edge sites and imperfect carbon structures.

When considered together with the XRD results, BP7003 reflects a balance between aromatic condensation and defect density. Such a combination is often favorable for adsorption, where π–π interactions and surface coordination may occur simultaneously.

#### 3.1.3. FTIR analysis

FTIR spectra (Fig. 2c) capture the transformation of functional groups during pyrolysis. The raw BP shows a broad band near ∼3400 cm^−1^ (O–H stretching), distinct C–H bands at ∼2920–2850 cm^−1^, a shoulder around ∼1730 cm^−1^ (C=O), and peaks near 1627 and 1037 cm^−1^ associated with aromatic C=C and C–O–C/C–O vibrations from lignocellulosic components.^52^

Thermal treatment progressively weakens these features. The C–H stretching bands diminish and nearly disappear. C–O-related bands shift and fade, while the signal near 1627 cm^−1^ broadens. At the same time, a band around ∼1400 cm^−1^ becomes more pronounced, consistent with increasing aromatic character. The decline of O–H and C–O–C functionalities reflects dehydration and decomposition of oxygenated aliphatic structures. What remains is a carbon matrix enriched in condensed sp^2^ domains.

This transformation involves a trade-off. Loss of oxygen-containing groups may reduce the number of coordination sites for metal ions, while the increase in aromatic domains favors interactions with organic molecules such as DR79 through π–π stacking.

#### 3.1.4. SEM-EDS analysis

##### 3.1.4.1. SEM analysis

Changes in the surface morphology and elemental distribution of the pyrochar were examined using SEM and EDS. SEM micrographs (Fig. 3 and S3 (SI)) reveal the progressive transformation of the bean pod biomass from a compact lignocellulosic structure into a porous carbonaceous framework with increasing pyrolysis temperature and residence time.

**Fig. 3:**
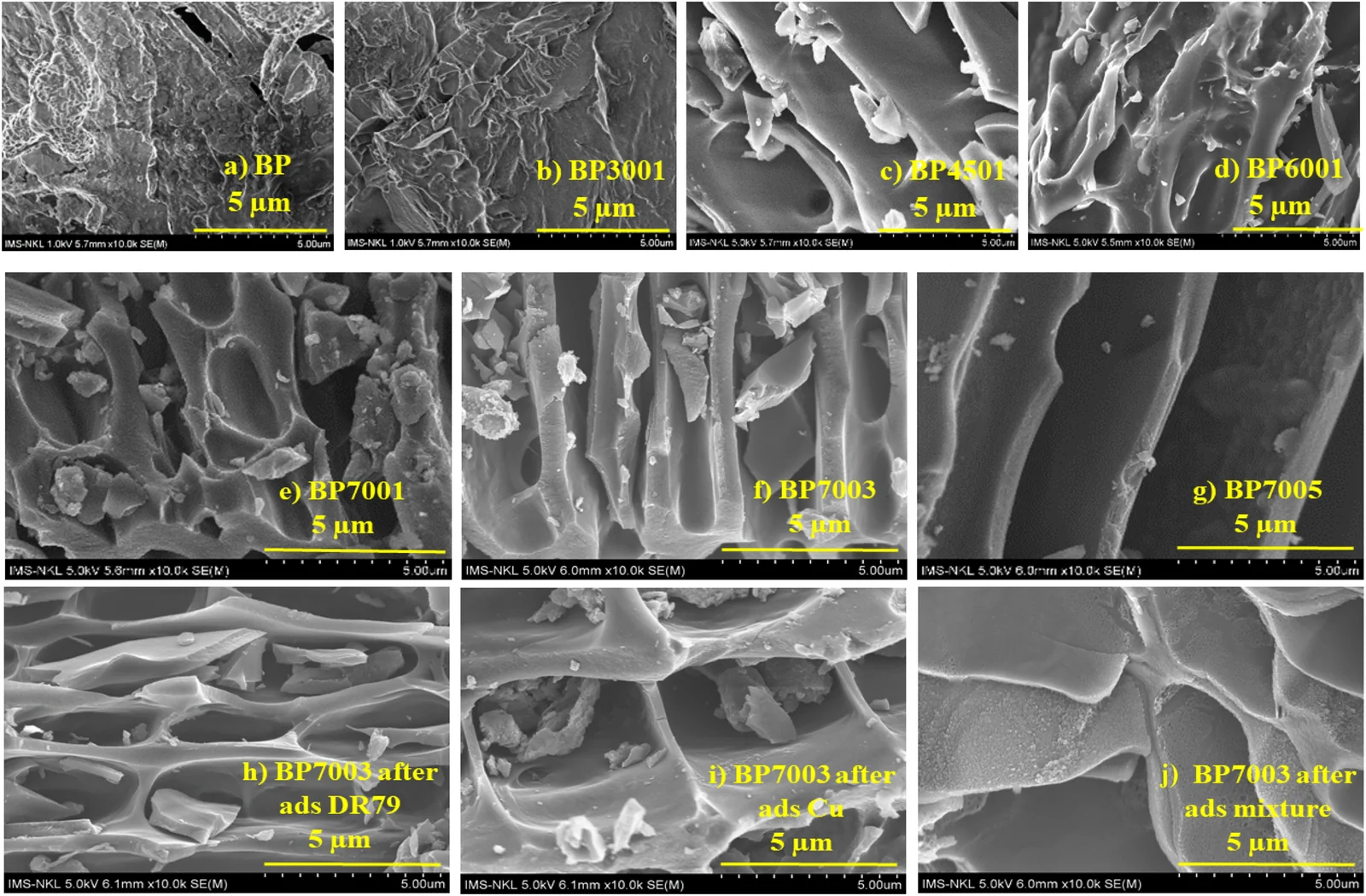
SEM images of BP and BP-derived pyrochars prepared at different pyrolysis temperatures, before and after adsorption: (a) BP; (b) BP3001; (c) BP4501; (d) BP6001; (e) BP7001; (f) BP7003; (g) BP7005; (h) BP7003 after DR 79 adsorption; (i) BP7003 after Cu adsorption; and (j) BP7003 after adsorption from the mixture. All images were recorded at a scale of 5 µm.

The raw BP exhibits the characteristic fibrous and lamellar morphology of lignocellulosic biomass. After pyrolysis at 300 °C (BP3001), the overall structure remains largely preserved, although partial shrinkage and the appearance of small fissures indicate the onset of hemicellulose decomposition and initial cellulose degradation. At 450 °C (BP4501), fragmentation becomes more pronounced, accompanied by the formation of slit-like openings and surface cavities associated with volatile release during biomass decomposition.

At 600 °C (BP6001), a more developed porous network appears. Carbon walls become thinner, and slit-like pores are visible. Most original plant structures are no longer distinguishable, indicating extensive structural rearrangement. The carbon matrix appears more rigid and layered, consistent with the development of turbostratic aromatic domains during advanced carbonization. At 700 °C (BP7001), larger pore domains show signs of partial fracture. However, the pore walls remain relatively thick and partially covered by irregular carbon fragments or residual condensed phases. Several pores appear isolated rather than fully interconnected, indicating that devolatilization and structural rearrangement are still incomplete at this stage. Extending the residence time to 3 h (BP7003) leads to the formation of larger carbon blocks with honeycomb-like internal structures. BP7003 exhibits the most optimized porous morphology. The SEM images reveal a highly interconnected three-dimensional pore network with open cavities, thinner carbon walls, and more uniform pore distribution. At higher magnification (Fig. S3 (SI)), the surface appears rough and highly corrugated, suggesting the coexistence of macro-, meso-, and possibly newly generated microporous domains formed during secondary carbonization. The absence of excessive tar residues together with the preservation of structural integrity indicates a balance between pore generation and framework stability. Such hierarchical porosity is highly favorable for maximizing *S*_BET_ because it simultaneously provides abundant adsorption sites and efficient diffusion channels. External surfaces become smoother, while small pores appear along the walls. Prolonged treatment (5 h, BP7005) introduces signs of mild sintering and partial pore coalescence, evidence of structural over-carbonization. Although large cavities remain visible, the pore walls become denser and smoother, while some smaller pores appear merged or partially collapsed. Localized wall thickening and structural shrinkage suggest mild thermal sintering and aromatically condensed carbon stacking during prolonged heating. These processes can reduce micropore volume and partially block accessible adsorption sites, leading to a decrease or plateau in *S*_BET_ despite the higher degree of carbonization.

Examination of SEM images (5 µm scale) reveals that the overall structure of BP7003 remained stable following the adsorption process. Initially, the capillary wall is very smooth, and the lumen is fully open (Fig. 3). After adsorption, numerous small bright spots appear on the capillary wall; the wall edges become rougher, and small particles appear adhering along the wall. These morphological changes indicate surface modification following the adsorption process; however, the bright regions were not assigned to specific adsorbates based on SEM morphology alone. Nevertheless, no significant agglomeration or fracturing of the carbon framework was observed, suggesting that the overall structural integrity of the biochar was maintained after adsorption.

SEM imaging provides qualitative information on surface morphology; however, it does not permit quantitative assessment of pore size distribution or specific surface area. These textural properties were therefore evaluated using N_2_ adsorption–desorption measurements, followed by BET analysis.

##### 3.1.4.2. EDS analysis

Complementary EDS analysis was performed to obtain semi-quantitative information on the elemental composition of the pyrochar materials and how it varies with pyrolysis temperature and residence time. The EDS results (Fig. 4 and Table 2) show an increasing relative contribution of carbon accompanied by a decreasing relative contribution of oxygen in the EDS-detected region with increasing pyrolysis severity. The raw BP contained approximately 60 at% C and 39 at% O, whereas BP3001 contained 76.72 at% C and 22.31 at% O. Further heating to 450–600 °C increased the relative C contribution to approximately 78–80 at% while reducing O to approximately 21–19 at%. At 700 °C, extending the residence time from 1 to 5 h increased the relative C contribution from 81.86 to 84.91 at%, whereas O decreased from 17.73 to 13.59 at%. These changes indicate a progressive shift toward a relatively carbon-enriched elemental composition in the EDS-detected region with increasing thermal severity.

**Fig. 4:**
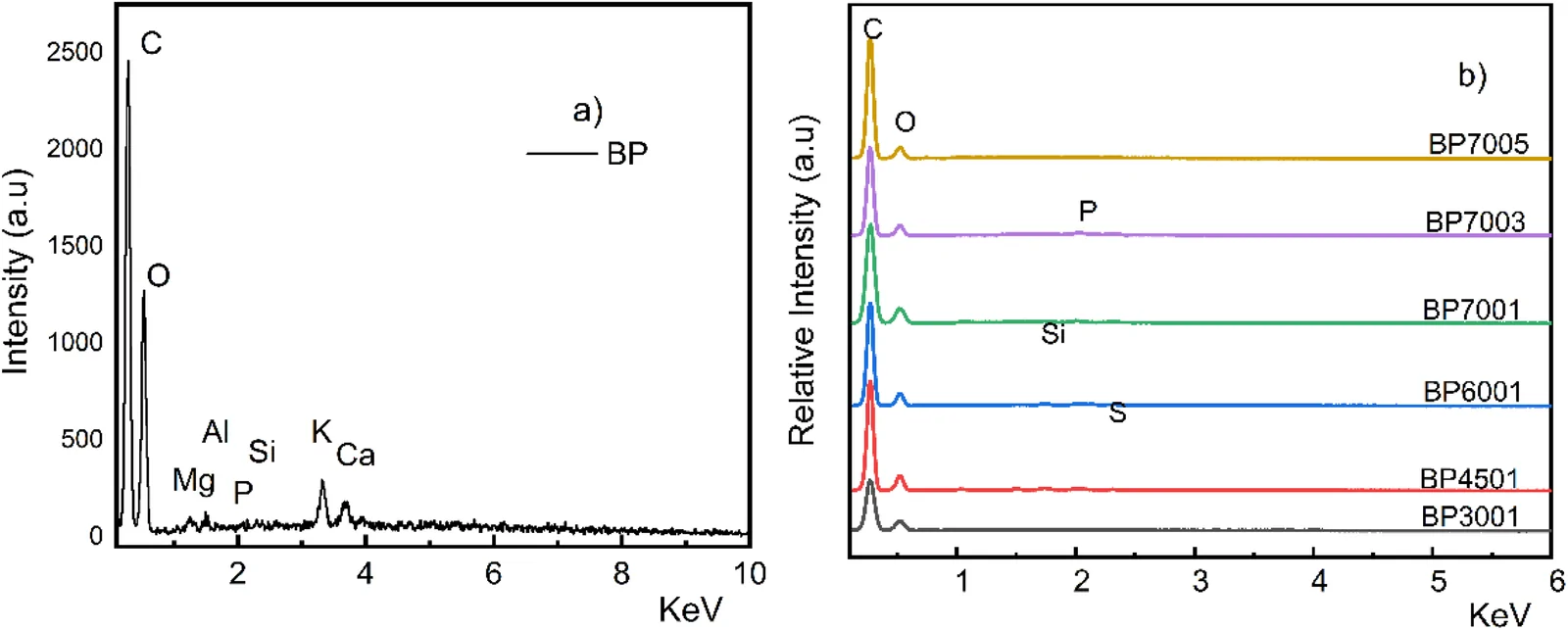
EDS spectra of BP (a) and BP-derived pyrochars (b) prepared at different pyrolysis temperatures and residence times.

**Table 2:** Elemental composition of BP-derived samples determined by EDS

Element	Atomic%									
	BP	BP3001	BP4501	BP6001	BP7001	BP7003	BP7005	BP7003 after adsorption		
								Cu(ii)	DR79	Cu(ii) + DR79
C	64.68	76.72	78.40	80.23	81.86	82.81	84.91	82.54	85.65	85.49
O	34.40	22.31	20.46	19.16	17.73	16.74	13.59	16.54	13.85	13.84
Mg	0.15	0.67	0.58	—	—	—	0.57	—	—	—
Al	0.13	—	—	—	—	—	—	—	—	—
Si	0.04	—	0.07	0.10	0.04	0.03	0.29	0.04	0.08	0.06
S	0.03	0.03	0.03	0.07	0.07	0.05	0.06	0.06	0.19	0.14
P	0.04	0.06	0.09	0.1	0.09	0.05	0.06	0.07	0.09	0.06
K	0.22	—	—	—	—	—	—	—	—	—
Ca	0.21	0.21	0.37	0.34	0.21	0.32	0.46	0.42	0.14	0.22
Cu	—	—	—	—	—	—	—	0.33	—	0.19

EDS alone cannot resolve the chemical pathways underlying these compositional changes. The observed evolution is therefore interpreted primarily as compositional evidence of pyrolysis-induced changes in the elemental composition of the carbonaceous material, while possible contributions from dehydration, devolatilization, and structural rearrangement are considered in conjunction with the complementary FTIR, Raman, and SEM results. The decreasing relative O/C ratio is consequently regarded as evidence of changing elemental composition rather than direct evidence of specific bonding environments or surface functional groups. Although the overall oxygen content decreased with increasing pyrolysis severity, FTIR analysis indicated that oxygen-containing functional groups were still retained in BP7003. These residual surface functionalities, together with its developed porous structure and large accessible surface area, may contribute to adsorbate–surface interactions.

At 300 °C (BP3001), carbon increases to ∼72%, while oxygen drops to ∼27%. This stage corresponds to partial carbonization, where dehydration and initial devolatilization occur but oxygen-containing groups are still abundant, indicating the initial decomposition of hemicellulose and partial devolatilization. This moderate chemical transformation is consistent with the SEM images showing only limited pore formation and partial preservation of the original biomass framework.

EDS mapping of BP7003 after adsorption is shown in Fig. S4 (SI), with the corresponding elemental compositions summarized in Table 2. Following Cu(ii) adsorption, Cu was detected in the EDS analysis, whereas no appreciable Cu signal was observed for pristine BP7003. Sulfur was also detected in the DR79-containing samples, consistent with the presence of S-containing species in the DR79-containing adsorption system. These results provide qualitative elemental evidence for the presence of Cu- and S-containing species after adsorption. However, the available spatial resolution and image quality do not allow the detected elemental signals to be reliably assigned to individual bright regions in the SEM images. Because EDS is semi-quantitative and the detected signal originates from an interaction volume rather than an atomically resolved surface layer, the EDS results are therefore interpreted as qualitative elemental evidence rather than direct evidence of specific molecular configurations or adsorption locations.

Overall, the EDS results complement the FTIR, Raman, XPS, zeta-potential, and textural analyses by providing semi-quantitative elemental and spatial information. They provide complementary evidence for changes in elemental composition during pyrolysis and for the presence of Cu- and S-containing species after adsorption, while chemical-state and bonding interpretations are appropriately constrained by the complementary spectroscopic evidence.

#### 3.1.5. BET analysis

The specific surface area and porosity of the pyrochars were evaluated using N_2_ adsorption–desorption measurements (Fig. 5a), while the pore-size distribution was determined using the BJH method based on the desorption branch of the nitrogen adsorption–desorption isotherm (Fig. 5b).

**Fig. 5:**
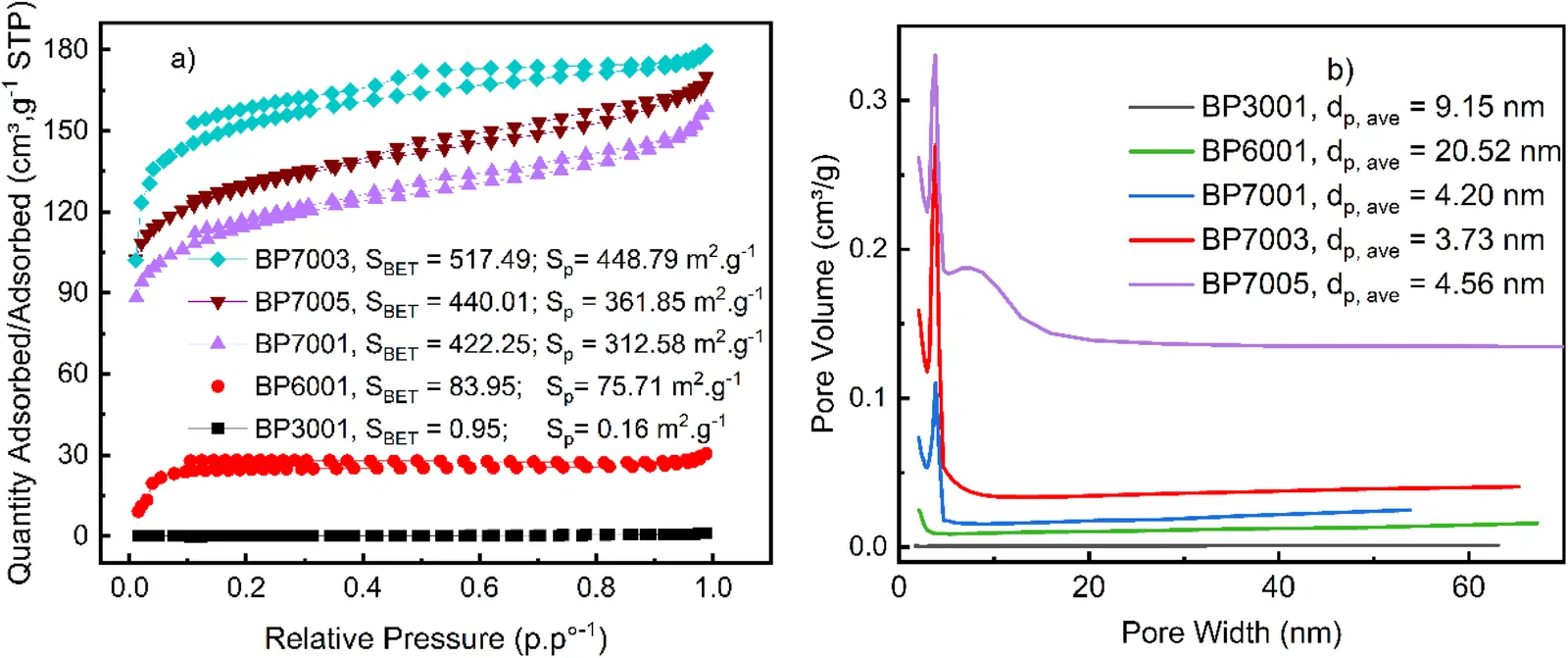
N_2_ adsorption–desorption isotherms (a) and pore-size distributions (b) of selected BP-derived pyrochars.

BP3001 exhibited a very low surface area (*S*_BET_ = 0.95 m^2^ g^−1^) with an average pore diameter of ∼9.15 nm. The t-plot-derived micropore surface area (*S*_p_) was only 0.15 m^2^ g^−1^, indicating a very limited microporous contribution. The SEM image also showed a relatively compact surface morphology. As the pyrolysis temperature increased, *S*_BET_ increased to 83.95 m^2^ g^−1^ for BP6001, accompanied by a relatively high *S*_p_ of 75.71 m^2^ g^−1^. The N_2_ adsorption increased rapidly from 9.2 to 19.6 cm^3^ g^−1^ as *P*/*P*_0_ increased from 0.015 to 0.039, followed by a more gradual increase at higher relative pressures. The hysteresis loop at intermediate relative pressures (*P*/*P*_0_ = 0.3–0.9) is consistent with capillary condensation associated with mesoporous domains. The isotherm exhibited features associated with both microporous and mesoporous adsorption, although the relatively limited N_2_ uptake at low relative pressure suggests that the microporous contribution remained modest. The BJH-derived average pore diameter of ∼24.76 nm falls within the mesopore range, supporting the presence of mesoporous features in BP6001. The H4 hysteresis is consistent with slit-like pore features.

A pronounced textural transition occurred at 700 °C. The BP700 samples exhibited increased N_2_ uptake at low relative pressure, with BP7003 showing the most pronounced increase, particularly over *P*/*P*_0_ ≈ 0.015–0.04. This behaviour is consistent with micropore filling at low relative pressure, whereas the hysteresis observed at intermediate relative pressures is consistent with capillary condensation associated with mesoporous domains. The t-plot-derived micropore surface area of BP7003 was 448.79 m^2^ g^−1^, corresponding to approximately 86.7% of its BET surface area (*S*_BET_ = 517.49 m^2^ g^−1^), providing quantitative support for a substantial microporous contribution under the applied N_2_ adsorption conditions.

The BJH analysis of the desorption branch indicated pore-size features mainly within the mesopore range (approximately 3.7–4.6 nm). These values are interpreted as indicative of mesopore-range features rather than as a quantitative description of microporosity. Accordingly, the microporous contribution is supported primarily by the low-pressure N_2_ adsorption behaviour and t-plot analysis, whereas BJH provides complementary information on the mesopore-range features. The SEM observations are interpreted as complementary evidence of the larger-scale surface morphology and are not used as direct evidence of microporosity. Together, the results support the coexistence of a substantial microporous contribution and complementary mesopore-range features in BP7003.

The marked increase in surface area at 700 °C is consistent with further development of accessible pore domains during pyrolysis, while SEM observations showed a more open surface morphology for BP7001–BP7003 than for BP6001.

Prolonging the residence time to 3 h (BP7003) further increased (*S*_BET_) to 517.49 m^2^ g^−1^ and the t-plot-derived micropore surface area (*S*_p_) to 448.79 m^2^ g^−1^, corresponding to approximately 86.7% of the BET surface area. This increase is consistent with a greater contribution from microporous domains to the accessible surface area under the applied characterization conditions. However, extending the residence time to 5 h reduced (*S*_BET_) to 440.01 m^2^ g^−1^ and (*S*_p_) to 361.85 m^2^ g^−1^, corresponding to approximately 82.2% of the BET surface area. The SEM images showed a relatively homogeneous and interconnected surface morphology for BP7003, whereas BP7005 exhibited partial pore coalescence, smoother carbon walls, and localized densification. These SEM observations are interpreted as complementary morphological evidence and are not used as direct evidence of microporosity. BP7003 showed the most pronounced low-pressure adsorption together with the highest t-plot-derived micropore surface area among the BP700 samples, supporting its relatively larger contribution from microporous domains to the accessible surface area.

The BJH analysis of the desorption branch indicated pore-size features mainly within the mesopore range (approximately 3.7–4.6 nm) for the BP700 samples. These values are interpreted as indicative of mesopore-range features rather than as a definitive quantitative description of the complete pore-size distribution. Accordingly, the BJH results are used as complementary information on the mesopore-range features, whereas the microporous contribution is supported primarily by the low-pressure N_2_ adsorption behaviour and t-plot analysis. SEM images revealed large pore openings and channel-like structures at the microscale, approximately 500 nm, which provide complementary evidence of larger-scale surface and pore morphology. These observations do not directly establish the presence or extent of microporosity. The relatively high (*S*_p_/*S*_BET_) ratio provides quantitative support for a substantial microporous contribution to the N_2_-accessible surface area, whereas larger pore features may provide additional pathways for mass transport. The increase in surface area at 700 °C was accompanied by changes in the pore characteristics, while extending the residence time from 1 to 3 h further increased (*S*_BET_) and (*S*_p_). Prolonged heating for 5 h, in contrast, reduced both parameters and increased the average pore diameter from 3.73 to 4.56 nm, consistent with partial pore widening and coalescence during extended thermal treatment.

Collectively, the N_2_ adsorption–desorption results indicate that BP7003 possessed the highest BET surface area among the investigated pyrochars, together with a substantial t-plot-derived microporous contribution and complementary mesopore-range features identified by BJH analysis. The larger-scale surface morphology observed by SEM provides complementary morphological information. Together, these results support the coexistence of a substantial microporous contribution and complementary mesopore-range features in BP7003.

### 3.2. Single adsorption of DR79 dye and heavy metal Cu(ii) ion

#### 3.2.1. Single adsorption of DR79

DR79 possesses a large molecular framework with multiple aromatic rings and distributed anionic groups, which impose steric constraints during adsorption. For this reason, DR79 was used as a primary probe to screen the prepared pyrochars. The initial comparison was conducted at 20 mg L^−1^, pH 4, and 180 min (Fig. 6a).

**Fig. 6:**
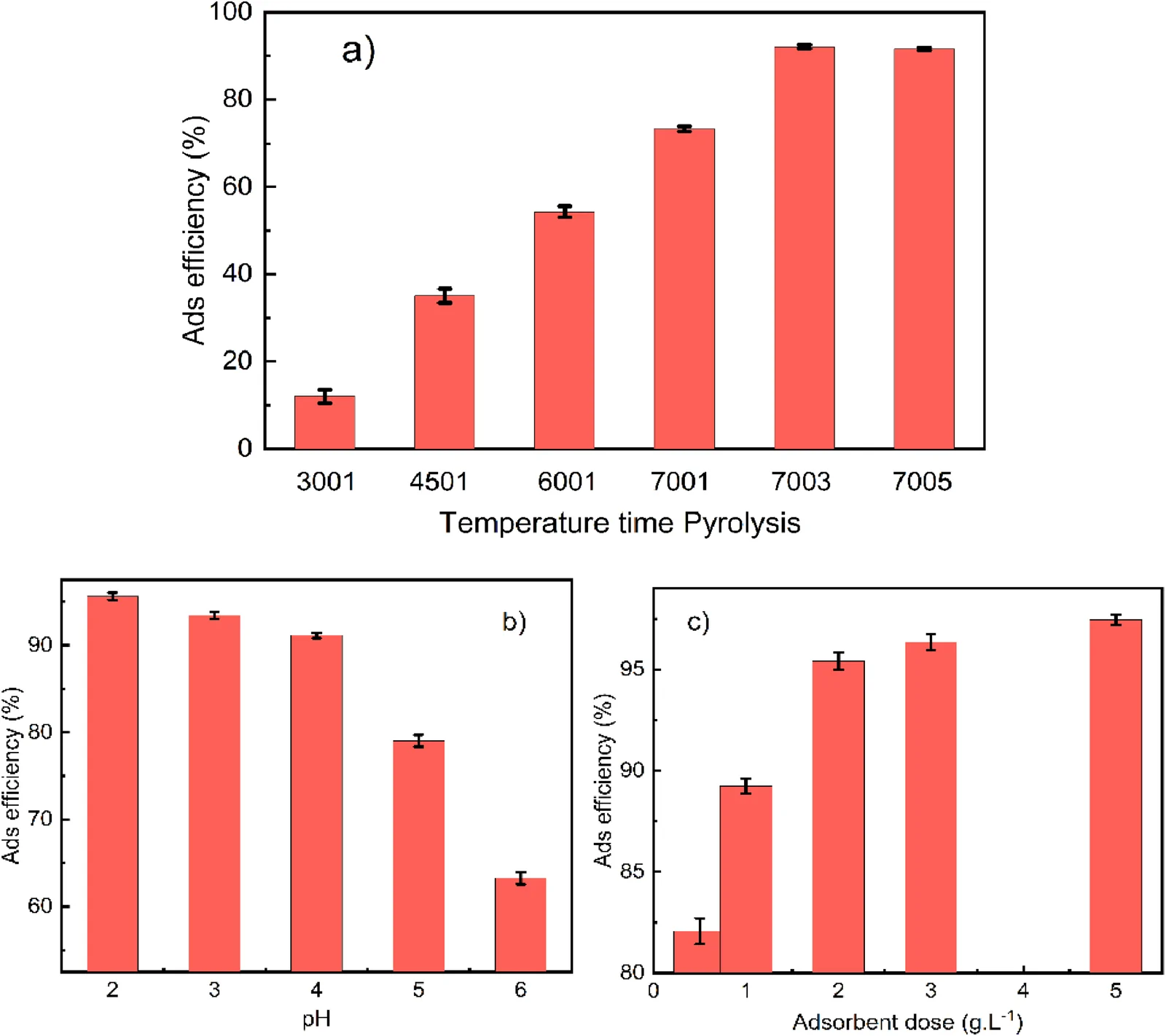
Effects of pyrolysis temperature and residence time (a), pH (b), and BP7003 dosage (c) on DR79 adsorption. The pH experiment used 20 mg per L DR79 and 1 g per L BP7003 for 180 min; the dosage experiment used 20 mg per L DR79 at pH 2 for 180 min. Error bars represent SD (*n* = 3).

When the pyrolysis temperature was varied at a fixed residence time of 1 h, adsorption efficiency increased progressively from 12.01 ± 1.51% (BP3001) to 73.29 ± 0.57% (BL7001). This trend follows the evolution of pore structure and carbon framework discussed in Section 3.1. The BET results show a substantial increase in specific surface area and pore surface area with increasing pyrolysis severity, particularly for the BP700-series samples. The development of accessible porosity may facilitate diffusion of the relatively large DR79 molecules and increase the accessibility of adsorption sites, contributing to the progressive enhancement in adsorption performance as pyrolysis severity increased. However, porosity alone does not fully explain the observed adsorption behavior. The increasing aromatic character of the carbon framework, evidenced by Raman analysis, may also contribute to interactions with the aromatic structure of DR79. At lower temperatures (300–450 °C), limited porosity and residual aliphatic components restrict access of bulky dye molecules. At 600–700 °C, the development of a more accessible porous structure together with defect-rich aromatic domains (Raman) may create more favorable adsorption environments for DR79 molecules. The adsorption efficiencies of BP7003 and BP7005 were 92.12 ± 0.41% and 91.63 ± 0.36%, respectively. Although the difference is relatively small, the slightly lower performance of BP7005 is consistent with the textural changes observed by BET analysis, including a decrease in specific surface area and an increase in average pore diameter. In contrast, Raman, FTIR, and XRD analyses indicate only minor differences in the overall chemical structure between the two samples. Therefore, the observed decrease in adsorption efficiency should be interpreted cautiously and may reflect modest changes in textural properties rather than substantial differences in surface chemistry. Among the investigated materials, BP7003 exhibited the highest adsorption efficiency and was therefore selected for subsequent characterization and adsorption studies. The favorable performance is consistent with its developed porosity and the heterogeneous nature of the carbon framework inferred from the physicochemical characterization results.

The effect of pH on DR79 adsorption was subsequently investigated using 20 mg per L DR79 at pH 2–6 for 180 min with 1 g per L BP7003 (Fig. 6b). The investigated pH range was limited to pH ≥ 2 because lower pH values would represent highly acidic conditions that are less representative of typical textile wastewater systems.

DR79 uptake decreased with increasing pH (Fig. 6b), consistent with a possible contribution from pH-dependent electrostatic interactions at the BP7003–solution interface. At lower pH, protonation of oxygen-containing surface functionalities may reduce the negative character of the surface, which may favour electrostatic attraction toward anionic DR79. With increasing pH, greater deprotonation of such functionalities could increase electrostatic repulsion and thereby contribute to the observed decrease in DR79 uptake. However, the zeta potential of BP7003 was measured only at pH 4 and therefore does not establish how the zeta potential varies across the investigated pH range. The pH-dependent adsorption trend should consequently be interpreted as evidence for a possible electrostatic contribution rather than direct evidence of a progressive change in surface charge. The persistence of measurable DR79 uptake at higher pH further indicates that electrostatic interactions alone cannot account for the overall adsorption behaviour, and additional short-range interactions with the carbon surface may contribute. Comparable pH-dependent behaviour has been reported for DR79 adsorption on carbon-based adsorbents.^53,54^

The effect of adsorbent dosage was then evaluated using 20 mg per L DR79 at pH 2 for 180 min, with BP7003 dosages ranging from 0.5 to 5.0 g L^−1^ (Fig. 6c). Removal efficiency increased rapidly between 0.5 and 2.0 g L^−1^, which can be attributed primarily to the greater availability of adsorption sites. Beyond this range, the removal efficiency approached a plateau. Under a fixed initial DR79 concentration, further increases in adsorbent dosage provide progressively less additional benefit, while diffusion and pore-accessibility constraints for the relatively large dye molecules may limit further uptake. Even at the highest dosage, complete removal was not achieved, consistent with incomplete accessibility of the available adsorption domains.

#### 3.2.2. Kinetics and isotherm behavior of DR79 on BP7003

Time-dependent uptake (5–180 min) and concentration–dependent adsorption (10–500 mg L^−1^) by 2 g per L BP7003 were evaluated to probe the adsorption process (Fig. 7).

**Fig. 7:**
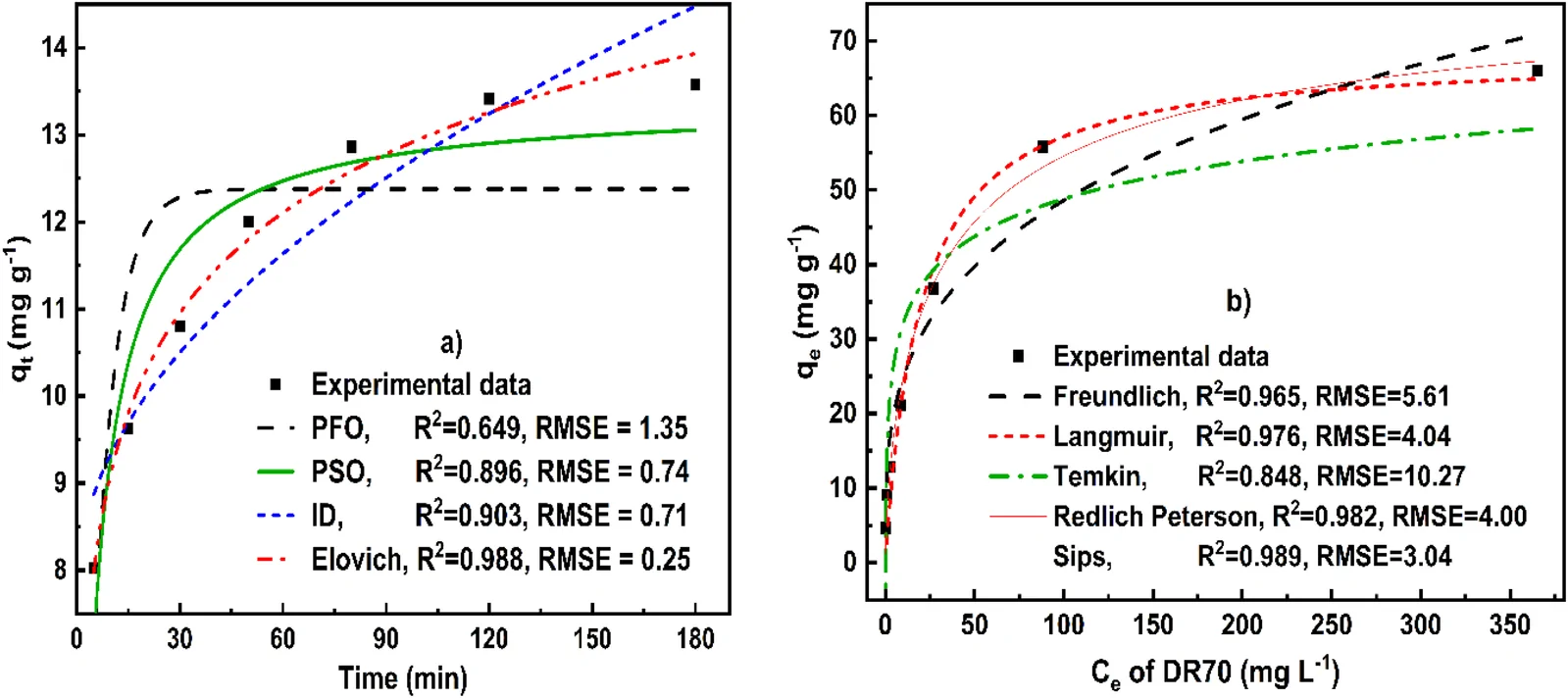
Effects of contact time (a) and initial DR79 concentration (b) on BP7003 adsorption at pH 2 using 2 g per L BP7003. Contact-time and equilibrium experiments covered 5–180 min and 10–500 mg per L DR79, respectively. Error bars represent SD (*n* = 3); curves show nonlinear model fits.

The kinetic curve shows that DR79 was adsorbed rapidly during the first 30 min (∼80%), followed by a slower uptake phase until approximately 120 min, when equilibrium was nearly reached. The numerical kinetic datasets used for model fitting are provided in Table S4 (SI). Among the tested kinetic models, the Elovich equation provided the closest description of the experimental data (*R*^2^ = 0.988 and RMSE = 0.25). This result suggests heterogeneous adsorption behaviour associated with a distribution of adsorption sites and/or adsorption energies on BP7003. However, the Elovich model does not by itself identify the underlying adsorption mechanism. The mechanistic discussion was therefore informed by the combined evidence from the kinetic behaviour and the physicochemical characteristics of BP7003, including its porous structure, surface functional groups, and electrokinetic properties. The intraparticle diffusion model also indicated a contribution of intraparticle diffusion to the adsorption process; however, the non-zero intercept suggests that intraparticle diffusion was not the sole rate-limiting step (Table 3).

**Table 3:** Kinetic parameters for single-solute adsorption of DR79 and Cu(ii) onto BP7003 at pH 2 and 5, respectively^a^

Adsorption isothermal models and parameters		DR79 (pH 2)	Cu(ii) (pH 5)
*C*_o_ (mg L^−1^)		28.703	19.328
*q*_e,exp_(mg g^−1^)		13.5783 ± 0.1371	9.5300 ± 0.0191
PFO	*R* ^2^	0.649	0.980
	RMSE	1.35	0.10
	*k*_1_ (min^−1^)	0.850 ± 0.040	0.098 ± 0.008
	*q*_e,cal_ (mg g^−1^)	12.38 ± 0.60	9.31 ± 0.15
PSO	*R* ^2^	0.896	0.991
	RMSE	0.74	0.04
	*k*_2_ (g mg^−1^ min^−1^)	0.017 ± 0.004	0.014 ± 0.001
	*q*_e,cal_ (mg g^−1^)	13.37 ± 0.44	10.13 ± 0.14
ID	*R* ^2^	0.904	0.673
	RMSE	0.71	1.58
	*k*_d_ (mg g^−1^ min^−0.5^)	0.501 ± 0.074	0.416 ± 0.130
	*C*_ID_ (mg g^−1^)	7.75 ± 0.61	5.05 ± 1.07
Elovich	*R* ^2^	0.988	0.878
	RMSE	0.25	0.59
	*k*_E_ (g mg^−1^)	0.60 ± 0.03	0.66 ± 0.12
	*a* (mg g^−1^ min^−1^)	39.91 ± 12.23	8.069 ± 6.566

a Initial DR79 and Cu(ii) concentrations were 28.703 and 19.328 mg L^−1^, respectively, with an adsorbent dosage of 2 g L^−1^. Experimental data were fitted using the pseudo-first-order (PFO), pseudo-second-order (PSO), intraparticle-diffusion (ID), and Elovich models. Values of experimental and fitted parameters are reported as mean ± standard deviation (SD) from three independent adsorption experiments (*n* = 3), whereas *R*^2^ and RMSE represent model-fitting statistics.

The equilibrium data were best described by the Sips model (*R*^2^ = 0.989, RMSE = 2.30), followed closely by the Redlich–Peterson model (*R*^2^ = 0.982, RMSE = 3.03) and the Langmuir model (*R*^2^ = 0.976, RMSE = 3.42). The better fit of the Sips model indicates that equilibrium adsorption is better represented by a heterogeneous surface with a finite distribution of adsorption sites. In contrast, the Langmuir model assumes energetically equivalent adsorption sites and therefore provides a simplified description of the adsorption behaviour. The Langmuir-derived *q*_m,cal_ was lower than the Sips-derived *q*_m,cal_ but closer to the maximum experimentally determined equilibrium adsorption capacity (*q*_e,exp_). This difference reflects the distinct assumptions of the two models rather than evidence for a specific adsorption mechanism. The Redlich–Peterson model also provided a good description of the equilibrium data, with *n*_RP_ = 0.894 indicating behaviour approaching the Langmuir limit while retaining deviation from ideal homogeneous adsorption.

Together, the kinetic and equilibrium modelling results consistently indicate that DR79 adsorption occurs on a heterogeneous adsorption landscape. However, these models do not by themselves establish whether the adsorption is predominantly chemisorptive or physisorptive, nor do they uniquely identify the molecular interactions involved. The contribution of pore accessibility, π–π interactions with aromatic domains, electrostatic interactions, and surface functional groups is therefore discussed separately based on the physicochemical and spectroscopic evidence.

The maximum experimentally determined equilibrium adsorption capacity of BP7003 for DR79 (*q*_e,exp_ ≈ 66 mg g^−1^) places it between highly engineered adsorbents and several low-cost adsorbents reported for Direct Red 79 removal. The adsorption performance of the present pyrochar is compared with representative adsorbents previously reported for DR79 removal, as summarized in Table 4.

**Table 4:** Comparison of the maximum adsorption capacities of representative adsorbents reported for Direct Red 79 removal

Adsorbent	*q*_max_ (mg g^−1^)	Reference
Fe_2_Fe_1−*x*_Mn_*x*_O_4_ spinels	183.99	55
Fe_1−*x*_Zn_*x*_Fe_2_O_4_ nanoparticles	95.30	54
**Black bean pod pyrochar**	**66.05**	**This work**
CTAB-modified natural laterite	54.95	53
Fe_1−*x*_Co_*x*_Fe_2_O_4_ nanoparticles	44.07	56
EDTA-modified Bellamya shell	10.78	57

Although the adsorption capacity of the present pyrochar is lower than that of engineered magnetic adsorbents, it is comparable to or higher than several low-cost adsorbents investigated for Direct Red 79 adsorption. Considering that the material was produced directly from black bean pods without chemical activation or post-synthesis surface modification, the observed performance highlights the potential of this biomass-derived pyrochar as a low-cost adsorbent for dye-removal applications.

#### 3.2.3. Single adsorption of Cu(ii) ion

According to the characterization results in Section 3.1, BP7003 exhibited the highest *S*_BET_ and a well-developed porous structure among the investigated pyrochars, together with residual oxygen-containing surface functionalities. These combined textural and surface-chemical characteristics may contribute to Cu(ii) adsorption. Although the adsorption behaviour of Cu(ii) differs from that of DR79 because of differences in molecular size, charge, and physicochemical interactions with the carbon surface, BP7003 was therefore selected for subsequent Cu(ii) adsorption experiments and evaluation of the Cu(ii)–DR79 binary system because it showed the highest DR79 adsorption efficiency under the initial screening conditions.

The effect of pH on Cu(ii) adsorption was investigated using 20 mg per L Cu(ii) and 2 g per L BP7003 for 180 min (Fig. 8a). The investigated pH range was restricted to pH ≤ 5 to minimize the potential contribution of solution-phase hydrolysis and precipitation to the measured Cu(ii) removal. Cu(ii) uptake increased with increasing pH, which may be attributed to progressive deprotonation of oxygen-containing surface functionalities and the resulting increase in the availability of surface sites for Cu(ii) interaction. At lower pH, protonation of these functionalities and competition from H^+^ may limit Cu(ii) uptake. No visible precipitation was observed under the investigated conditions. A pH of 5 was therefore selected for the subsequent Cu(ii) adsorption experiments. This trend is consistent with the oxygen-containing surface functionalities identified by FTIR, which may contribute to Cu(ii) uptake. The results are presented in Fig. 8a and Table 3.

**Fig. 8:**
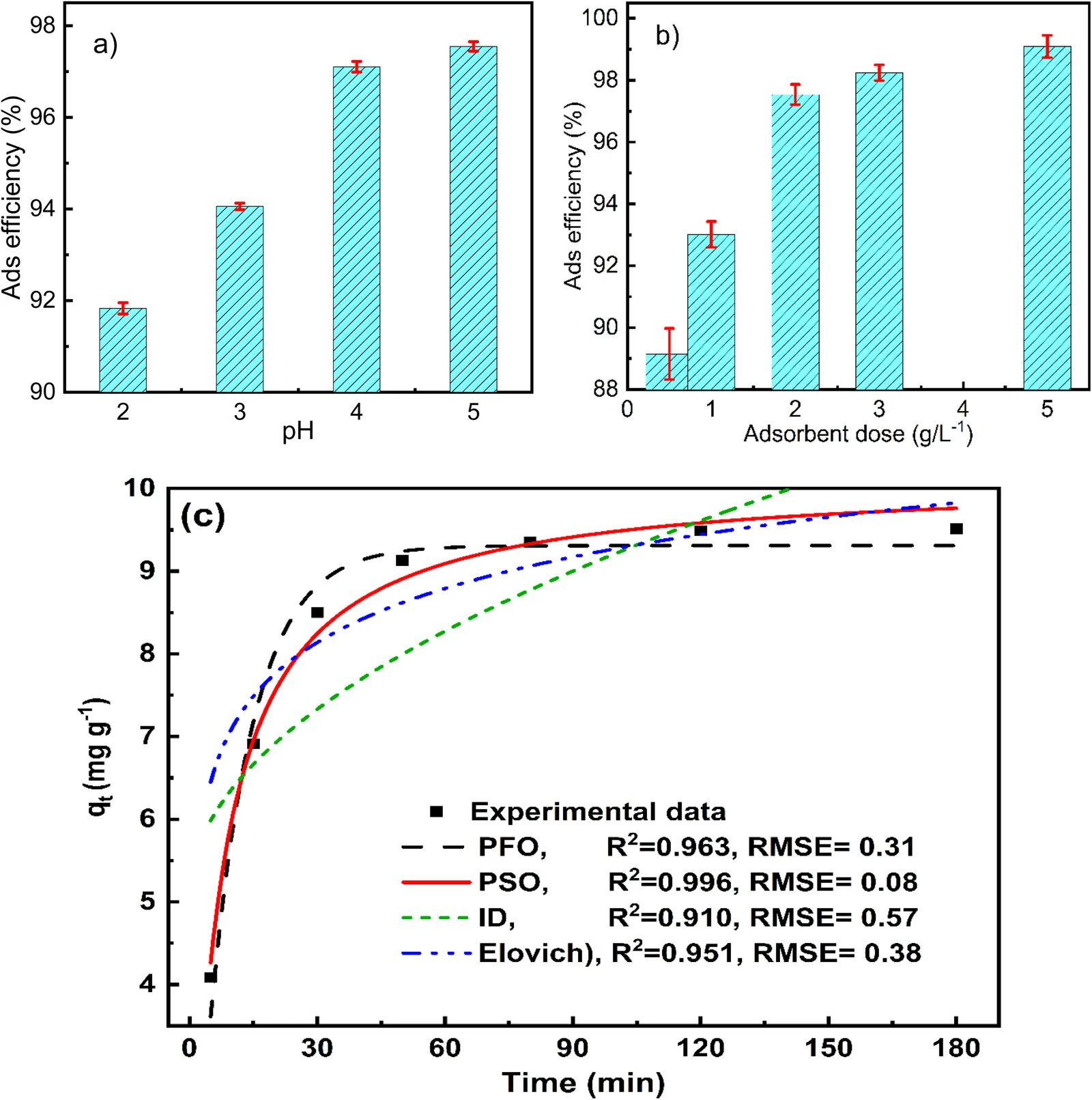
Effects of (a) pH and (b) BP7003 dosage on Cu(ii) adsorption, and (c) adsorption kinetics with nonlinear model fitting using the pseudo-first-order (PFO), pseudo-second-order (PSO), intraparticle diffusion (ID), and Elovich models. The initial Cu(ii) concentration was 20 mg L^−1^. The pH and dosage experiments were conducted for 180 min using 2 g L^−1^ BP7003 at pH 5, respectively. Error bars represent standard deviations (SD, *n* = 3); curves show nonlinear model fits.

Effect of BP7003 dosage on Cu(ii) removal was subsequently investigated over the range of 0.5–5.0 g L^−1^ (Fig. 8b). Increasing the adsorbent dosage from 0.5 to 3.0 g L^−1^ progressively increased the Cu(ii) removal efficiency from approximately 90.5% to 99.0%. A further increase to 5.0 g L^−1^ resulted in only a marginal improvement, with the removal efficiency reaching approximately 99.2%. The increase in removal efficiency is consistent with the greater amount of adsorbent available for Cu(ii) uptake at higher dosages. The relatively small improvement above 3.0 g L^−1^ indicates that Cu(ii) removal approached a plateau under the investigated conditions.

The adsorption kinetics of Cu(ii) were investigated using 20 mg per L Cu(ii) and 2 g per L BP7003 at pH 5. Cu(ii) uptake increased rapidly during the first 30 min (∼89%) and subsequently slowed, approaching equilibrium at approximately 80 min (Fig. 8c). The kinetic data were fitted using the pseudo-first-order (PFO), pseudo-second-order (PSO), Elovich, and intraparticle-diffusion (ID) models (Table 3). The PSO model provided a better empirical fit (*R*^2^ = 0.991; RMSE = 0.04 mg g^−1^) than the PFO model (*R*^2^ = 0.980; RMSE = 0.10 mg g^−1^), whereas the Elovich and ID models showed lower fitting performance (*R*^2^ = 0.878 and 0.673, respectively). These differences describe how well the respective equations reproduce the experimental uptake curve and should not, by themselves, be interpreted as definitive evidence for a particular molecular adsorption mechanism or rate-controlling step. In particular, the better PSO fit was not taken as standalone evidence of chemisorption, electron sharing, or electron exchange. Likewise, the Elovich and ID models were used as empirical descriptors of the kinetic behaviour rather than as standalone evidence of surface heterogeneity or intraparticle diffusion as the rate-controlling process.

The equilibrium data were fitted using the Langmuir, Freundlich, Temkin, Redlich–Peterson, and Sips models, as shown in Fig. 9 and summarized in Table 5. The numerical equilibrium dataset used for model fitting is provided in Table S5A (SI). The Sips model provided the best empirical fit to the Cu(ii) equilibrium data (*R*^2^ = 0.996, RMSE = 2.53 mg g^−1^), followed by the Redlich–Peterson model (*R*^2^ = 0.992, RMSE = 3.80 mg g^−1^). The Freundlich and Langmuir models showed comparable fits (*R*^2^ = 0.986 and 0.985, respectively), whereas the Temkin model showed a weaker fit (*R*^2^ = 0.891, RMSE = 13.81 mg g^−1^).

**Fig. 9:**
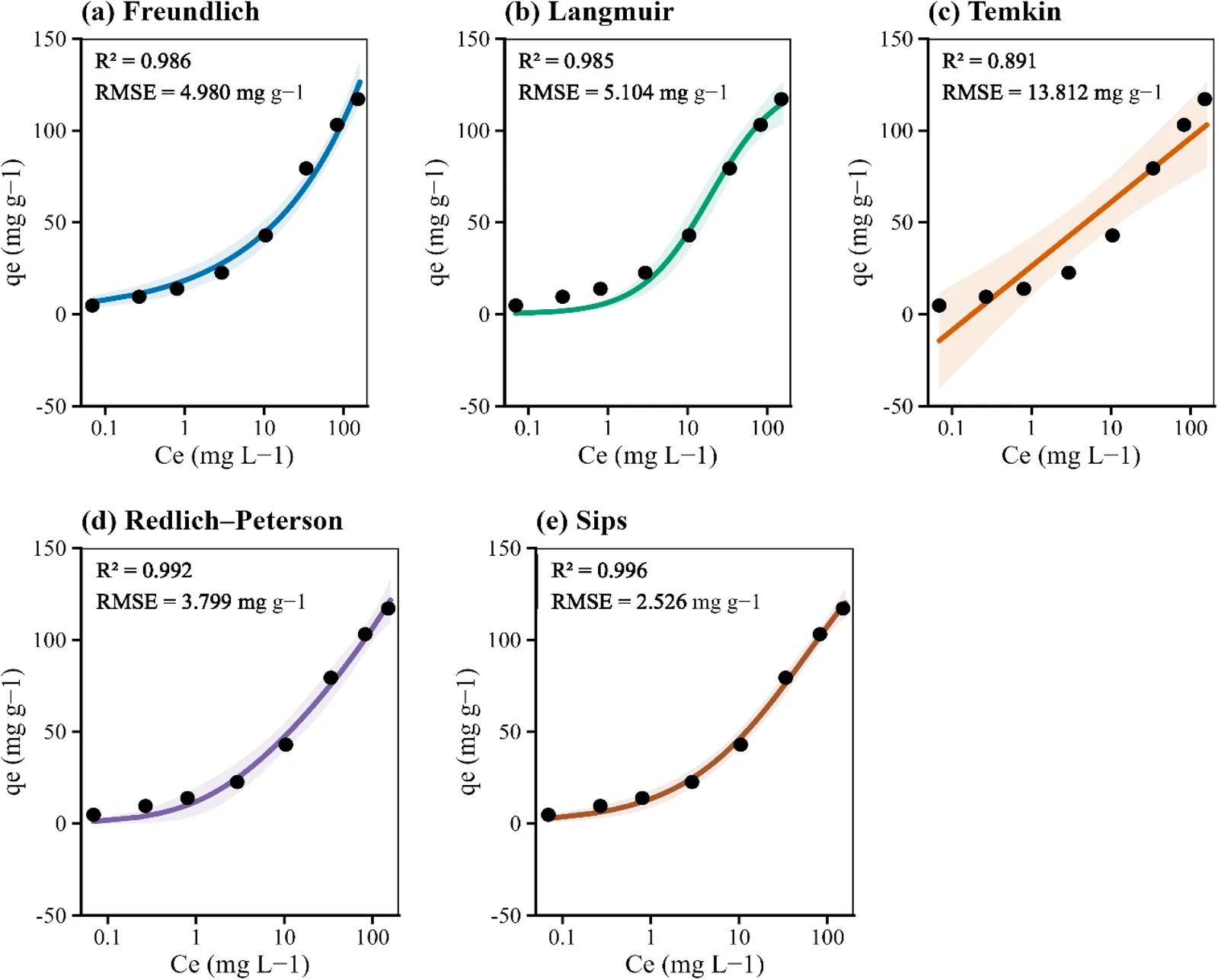
Nonlinear fitting of the equilibrium adsorption isotherms of Cu(ii) onto BP7003 at pH 5 using a BP7003 dosage of 2 g L^−1^ and initial Cu(II) concentrations of 10–400 mg L−1: (a) Freundlich, (b) Langmuir, (c) Temkin, (d) Redlich–Peterson, and (e) sips models. Black symbols represent the experimental data, solid curves represent the fitted models, and shaded regions indicate the associated uncertainty bands.

**Table 5:** Equilibrium isotherm parameters for DR79 and Cu(ii) adsorption onto BP7003 at pH 2 and 5, respectively. Initial adsorbate concentrations were 10–500 mg L^−1^ for DR79 and 10–400 mg L^−1^ for Cu(ii). Experimental data were fitted using the indicated isotherm models

Adsorption isothermal models and parameters		DR79 (pH 2)	Cu(ii) (pH 5)
*q*_e,exp_ (mg g^−1^)		66.05 ± 1.15	117.13 ± 0.95
Freundlich	*R* ^2^	0.955	0.986
	RMSE (mg g^−1^)	4.74	4.98
	*K*_F_ (mg^1−1/*n*_F_^ L^1/*n*_F_^ g^−1^)	12.72 ± 2. 39	18.39 ± 2.38
	*n*_F_ (dimensionless)	0.291 ± 0 .033	0.380 ± 0.029
Langmuir	*R* ^2^	0.976	0.985
	RMSE (mg g^−1^)	3.41	5.10
	*K*_L_ (L mg^−1^)	0.051 ± 0.011	0.052 ± 0.011
	*q*_m,cal_ (mg g^−1^)	68.46 ± 4.14	128.92 ± 7.42
Temkin	*R* ^2^	0.848	0.891
	RMSE (mg g^−1^)	8.68	13.81
	*K*_*T*_ (L g^−1^)	8.19 ± 7.65	5.59 ± 3.32
	*B*_*T*_ (mg g^−1^)	7.28 ± 1.38	15.17 ± 2.17
Redlich–Peterson	*R* ^2^	0.982	0.992
	RMSE (mg g^−1^)	3.03	3.80
	*K*_RP_ (L g^−1^)	5.26 ± 2.15	17.75 ± 8.91
	*B*_RP_ (mg^−*n*_RP_^ L^*n*_RP_^)	0.142 ± 0.117	0.488 ± 0.401
	*n*_RP_ (dimensionless)	0.894 ± 0.077	0.753 ± 0.069
Sips	*R* ^2^	0.989	0.996
	RMSE (mg g^−1^)	2.30	2.53
	*q*_m,cal_ (mg g^−1^)	80.84 ± 10.24	185.38 ± 30.80
	*K*_S_ (L g^−1^)	0.030 ± 0.015	0.017 ± 0.010
	*n*_S_ (dimensionless)	0.674 ± 0.119	0.622 ± 0.072

The Langmuir fit yielded *q*_m_,_cal_ = 128.82 ± 7.42 mg g^−1^, which is of similar magnitude to the experimentally determined equilibrium uptake (*q*_e_,_exp_ = 117.13 ± 0.95 mg g^−1^). The Langmuir-derived *q*_m_ is therefore regarded as a model-derived parameter rather than an independently measured saturation capacity. The relatively good empirical fits obtained with the Sips and Redlich–Peterson models suggest that the equilibrium behaviour is not fully described by the assumptions of the Langmuir model and may be consistent with heterogeneous adsorption behaviour. However, these models alone do not establish the molecular origin of this heterogeneity.

BP7003 exhibited faster Cu(ii) uptake and a higher equilibrium adsorption capacity (∼117.1 mg g^−1^) than DR79. The faster uptake may be related to the smaller hydrated size of Cu(ii), which can facilitate access to porous domains and interaction with oxygen-containing surface functionalities. The observed adsorption capacity is comparable to those reported for several pristine biochars and composite adsorbents,^58–60^ although chemically modified systems may exhibit higher capacities.^61^

The performance observed here can be attributed to the combined effects of accessible porosity and residual oxygen-containing functional groups, which together support both electrostatic attraction and surface complexation.

### 3.3. Simultaneous adsorption of DR79 and Cu(ii)

#### 3.3.1. Simultaneous adsorption conditions

To explore co-adsorption behaviour, experiments were conducted at pH 4, between the selected single-solute conditions of pH 2 for DR79 and pH 5 for Cu(ii). This common pH was selected to evaluate the simultaneous adsorption of both species without favouring the optimum condition of either single-solute system. Before adsorption, possible interactions between Cu(ii) and DR79 in solution at pH 4 were examined by UV-Vis spectroscopy (Fig. 10a). Mixing Cu(ii) with DR79 produced only minor spectral changes. The main absorption maximum of DR79 remained near 500 nm, with only a slight shift in peak position, while the overall spectral profiles remained highly similar and no distinct new absorption band appeared. These observations provide no clear UV-Vis evidence for the formation of a strongly absorbing Cu(ii)–DR79 species under the investigated conditions. The minor spectral differences may reflect changes in the local solution environment rather than formation of a distinct bulk–solution complex.

**Fig. 10:**
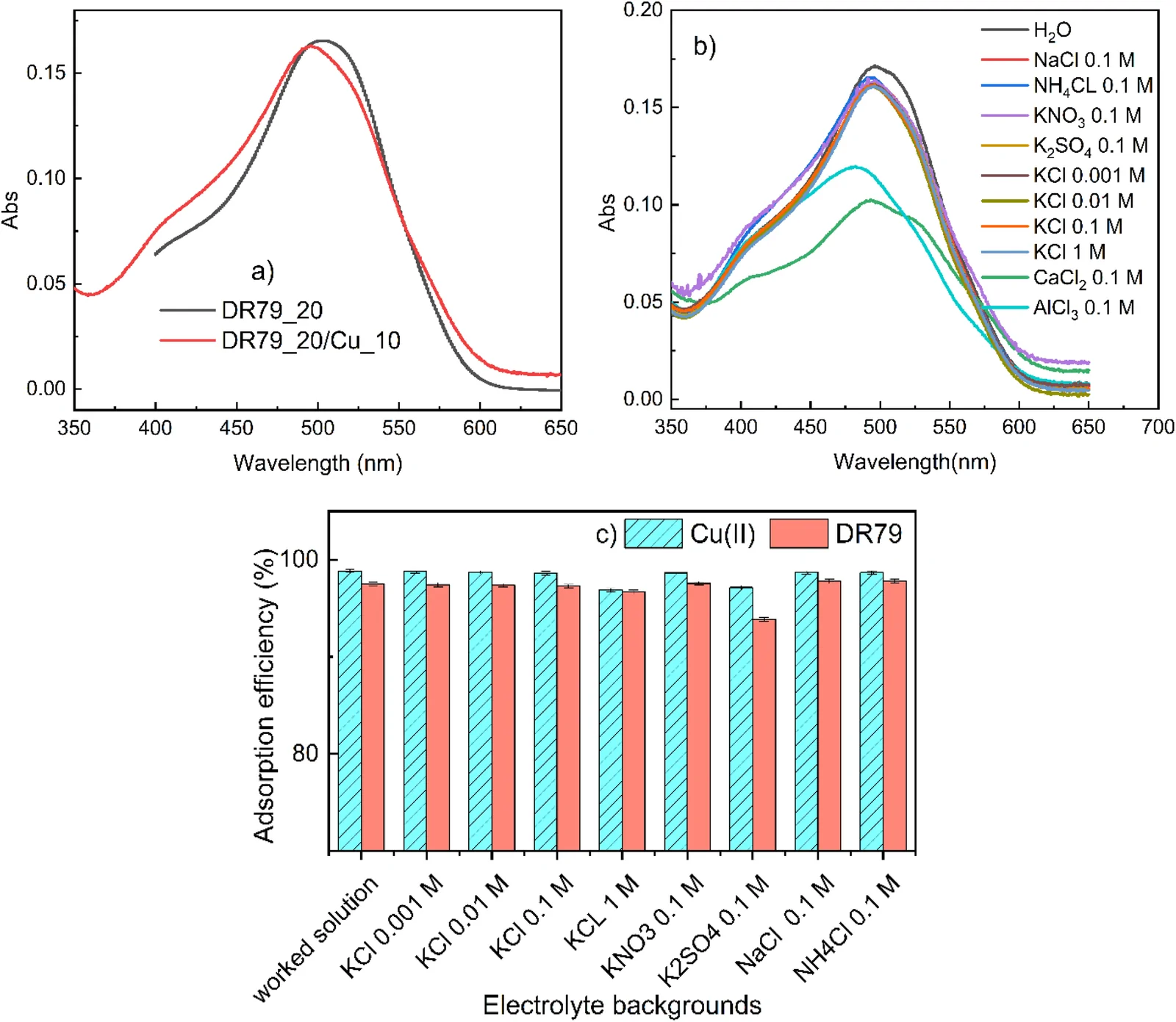
UV-Vis spectra of 20 mg per L DR79 and mixed DR79/Cu(ii) solutions (20 and 10 mg L^−1^, respectively) in different electrolyte backgrounds (a and b); (c) simultaneous adsorption efficiencies of Cu(ii) and DR79 by BP7003 in the corresponding electrolyte solutions. Error bars represent SD (*n* = 3).

The UV-Vis spectra of this mixed system in various electrolyte media were also investigated (Fig. 10b). In NaCl, NH_4_Cl, KCl, KNO_3_, and K_2_SO_4_ electrolyte matrices, the spectral trace almost completely overlaps with the spectrum obtained in a deionized water matrix at pH 4, however, the maximum absorption peak intensity decreases slightly, ranging from 99.21% to 98.57% and down to 97.84% as KCl concentration increased to 1 M. In contrast, multivalent cations (Ca^2+^, Al^3+^) induced noticeable spectral shifts and intensity changes, consistent with possible dye aggregation and/or stronger coordination interactions. These observations establish that, in the absence of competing multivalent ions, the Cu(ii)–DR79 interaction in bulk solution remains limited, and any coupling observed during adsorption is more likely surface-mediated.

Simultaneous adsorption of 20 mg per L Cu(ii) and 10 mg per L DR79 by 2 g per L BP7003 at pH 4 for 3 h was also investigated in these electrolyte media, excluding CaCl_2_ and AlCl_3_; the results are shown in Fig. 10c. The DR79 adsorption efficiency was estimated from the UV-Vis spectra using the ratio of the absorbance before and after adsorption at the corresponding *λ*_max_ for each electrolyte medium. Electrolyte effects (Fig. 10c) provide further insight into the possible contributions of ionic strength and ion-specific interactions to the adsorption process. Increasing KCl concentration slightly decreased the adsorption efficiency of both Cu(ii) and DR79, consistent with electrical-double-layer compression and electrostatic screening with increasing ionic strength. The limited effects of the tested monovalent electrolytes (NaCl, KNO_3_ and NH_4_Cl) suggest that ionic strength alone may not fully account for the observed electrolyte dependence.

Sulfate-containing electrolytes produced a more pronounced decrease in adsorption efficiency. At the same nominal concentration (0.1 M), K_2_SO_4_ has a higher ionic strength (*I* ≈ 0.30 M) than 1 : 1 electrolytes such as KCl or NaCl (*I* ≈ 0.10 M), and therefore can induce stronger electrostatic screening. Sulfate may also exert ion-specific effects, potentially influencing surface interactions relevant to DR79 adsorption and the aqueous chemical environment of Cu(ii). However, because ionic strength and electrolyte identity were not independently controlled, their relative contributions cannot be quantitatively separated. The stronger sulfate effect should therefore be interpreted as reflecting a combination of its greater ionic-strength contribution and possible ion-specific interactions, rather than being attributed to competition alone.

Accordingly, subsequent binary adsorption experiments were conducted in aqueous medium without added electrolyte at pH 4 to avoid introducing an additional electrolyte-matrix variable.

#### 3.3.2. Binary adsorption kinetics

The simultaneous uptake of Cu(ii) (20 mg L^−1^) and DR79 (10 mg L^−1^) at pH 4 is shown in Fig. 11. Both species exhibited rapid initial uptake during the first 20 min, followed by a slower approach to equilibrium at approximately 60–90 min. This biphasic uptake behaviour is consistent with rapid occupation of accessible adsorption domains followed by slower uptake as surface coverage increased. The kinetic parameters are summarized in Table 6.

**Fig. 11:**
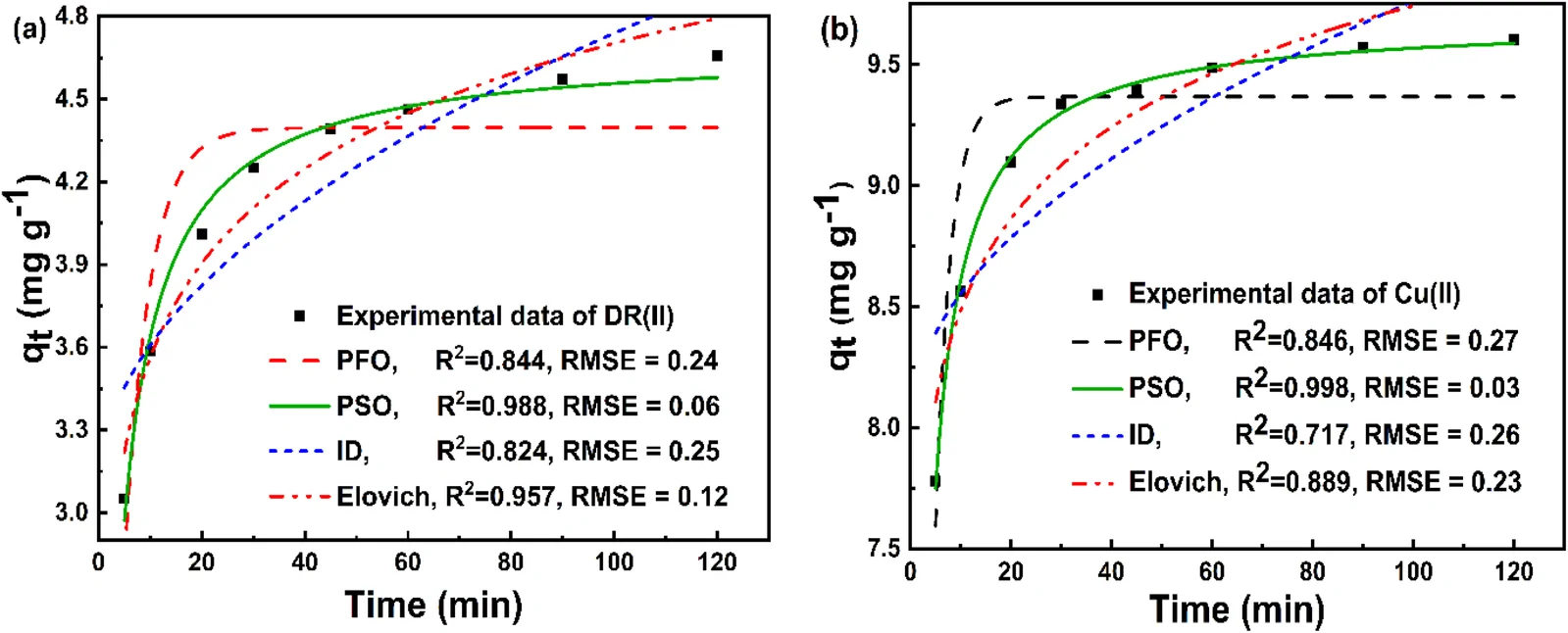
Adsorption kinetics of DR79 (a) and Cu(ii) (b) in the binary system using 2 g per L BP7003 at pH 4, with initial DR79 and Cu(ii) concentrations of 10 and 20 mg L^−1^, respectively. Experimental data were fitted using the indicated kinetic models; error bars represent SD (*n* = 3).

**Table 6:** Kinetic parameters for binary Cu(ii)–DR79 adsorption onto BP7003 under the investigated conditions

Kinetic models and parameters		DR79, *C*_o_ = 10 mg L^−1^	Cu(ii), *C*_o_ = 20 mg L^−1^
*q*_e,exp_ (mg g^−1^)		4.66 ± 0.003	9.60 ± 0.003
PFO	*R* ^2^	0.844	0.846
	RMSE	0.24	0.27
	*k*_1_ (min^−1^)	0.204 ± 0.027	0.333 ± 0.031
	*q*_e,cal_ (mg g^−1^)	4.40 ± 0.10	9.37 ± 0.11
PSO	*R* ^2^	0.988	0.998
	RMSE	0.06	0.03
	*k*_2_ (g mg^−1^ min^−1^)	0.074 ± 0.005	0.083 ± 0.002
	*q*_e,cal_ (mg g^−1^)	4.69 ± 0.04	9.69 ± 0.02
ID	*R* ^2^	0.824	0.233
	RMSE	0.25	0.36
	*k*_ID_ (mg g^−1^ min^−0.5^)	0.166 ± 0.031	0.176 ± 0.045
	*C*_ID_ (mg g^−1^)	3.084 ± 0.215	7.997 ± 0.312
Elovich	*R* ^2^	0.957	0.369
	RMSE	0.12	0.23
	*k*_E_ (g mg^−1^)	2.017 ± 0.176	1.832 ± 0.266
	*a* (mg g^−1^ min)	65.14 ± 42.03	307 691 ± 70 050

The adsorption kinetics of DR79 and Cu(ii) in the binary system were evaluated using the pseudo-first-order (PFO), pseudo-second-order (PSO), intraparticle diffusion (ID), and Elovich models (Table 6). For DR79, the PSO model provided the best empirical fit to the observed uptake kinetics (*R*^2^ = 0.988; RMSE = 0.06), while the Elovich model also provided a good fit (*R*^2^ = 0.957; RMSE = 0.12). For Cu(ii), the PSO model provided the best empirical fit (*R*^2^ = 0.998; RMSE = 0.03). The ID model showed weaker agreement, particularly for Cu(ii) (*R*^2^ = 0.233), while the non-zero intercepts indicate that intraparticle diffusion was not the sole process governing the observed uptake. These results characterize the kinetic behaviour of the binary system. Because the corresponding single-solute experiments were conducted at different pH values and, for DR79, a different initial concentration (Table S3 (SI)), direct comparison of kinetic parameters between the single-solute and binary systems was not attempted. The PSO fits therefore provide an empirical description of the observed uptake kinetics and do not, by themselves, establish a specific adsorption mechanism.

However, because the number of available adsorption sites remains finite, the equilibrium adsorption capacity increases only slightly. This result is consistent with the DR79 adsorption kinetics experiment in the mixture, the process occurring very rapidly in the initial period, and is consistent with the change in material zeta potential presented in 3.4.

#### 3.3.3. The simultaneous adsorption of DR79 and Cu(ii) by BP7003

The adsorption capacities for individual and mixed systems are presented in Fig. 12a, b, Tables 7 and S6A (SI).

**Fig. 12:**
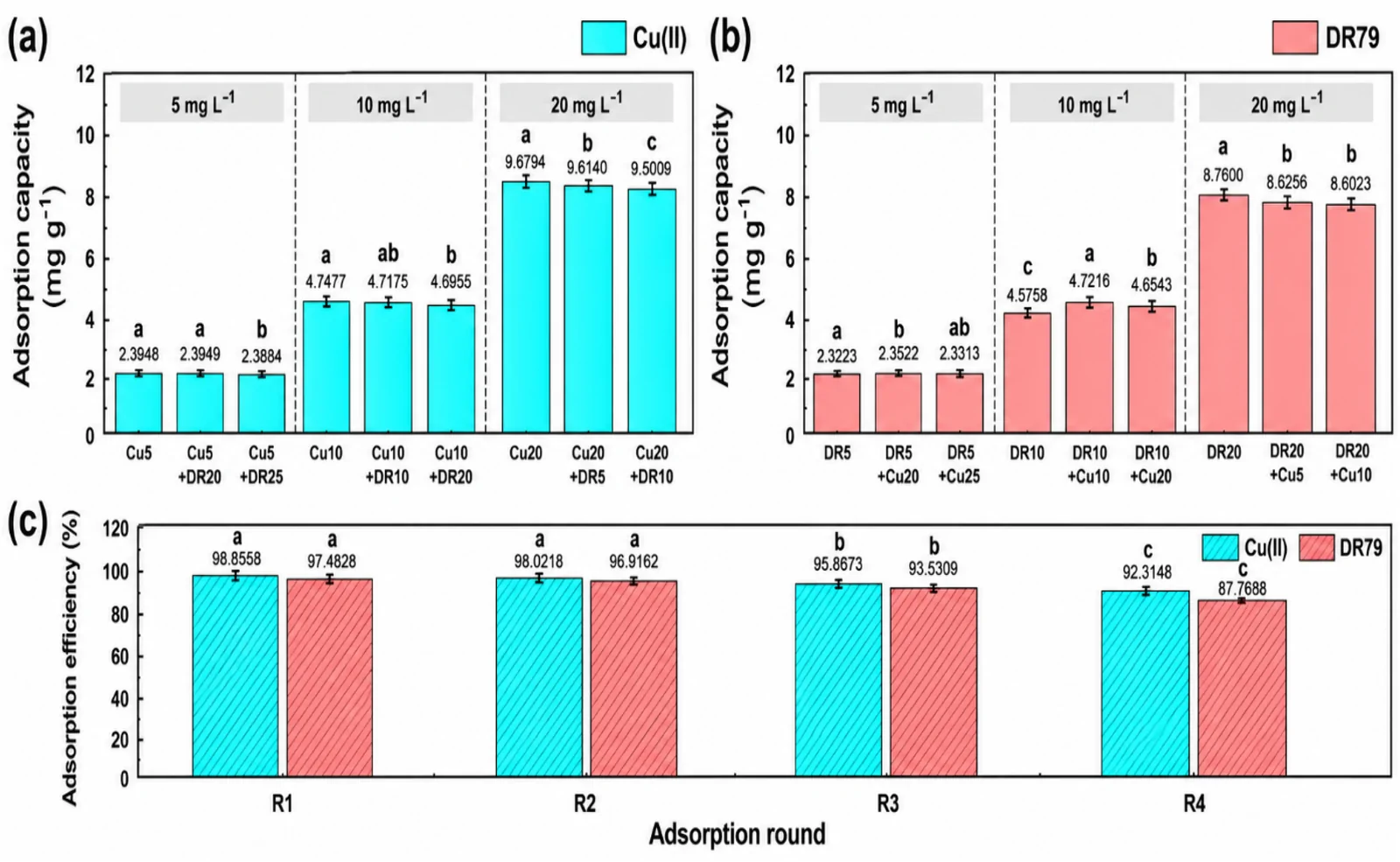
Adsorption of Cu(ii) and DR79 onto BP7003 in single- and binary-solute systems under their respective experimental conditions: (a) Cu(ii) adsorption, (b) DR79 adsorption, and (c) regeneration performance. Binary adsorption experiments were conducted at pH 4. Error bars represent standard deviation (SD) from three independent adsorption experiments (*n* = 3).

**Table 7:** Descriptive comparison of single- and binary-solute adsorption of Cu(ii) and DR79 onto BP7003 under matched initial-concentration conditions^a^

Adsorbate	Single-solute system	Binary-solute system	*q*_single_ (mg g^−1^)	*q*_binary_ (mg g^−1^)	*q*_binary_/*q*_single_	Change (%)
Cu(ii)	Cu5	Cu5/DR20	2.3948	2.3949	1.0000	+0.00
Cu(ii)	Cu5	Cu5/DR25	2.3948	2.3884	0.9973	−0.27
Cu(ii)	Cu10	Cu10/DR10	4.7477	4.7175	0.9936	−0.64
Cu(ii)	Cu10	Cu10/DR20	4.7477	4.6955	0.9890	−1.10
Cu(ii)	Cu20	Cu20/DR5	9.6794	9.6140	0.9932	−0.68
Cu(ii)	Cu20	Cu20/DR10	9.6794	9.5009	0.9816	−1.84
DR79	DR5	DR5/Cu20	2.3223	2.3522	1.0129	+1.29
DR79	DR5	DR5/Cu25	2.3223	2.3313	1.0039	+0.39
DR79	DR10	DR10/Cu10	4.5758	4.7216	1.0319	+3.19
DR79	DR10	DR10/Cu20	4.5758	4.6543	1.0172	+1.72
DR79	DR20	DR20/Cu5	8.7600	8.6256	0.9847	−1.53
DR79	DR20	DR20/Cu10	8.7600	8.6023	0.9820	−1.80

a Values are presented as mean ± SE from three independent adsorption experiments (*n* = 3). The *q*_binary_/*q*_single_ ratio was calculated from the corresponding mean adsorption capacities. Positive and negative changes represent enhancement and inhibition, respectively. Although the initial concentration of the adsorbate of interest was matched between the corresponding single- and binary-solute experiments, the experimental pH was not identical (pH 2 for DR79 single-solute adsorption, pH 5 for Cu(ii) single-solute adsorption, and pH 4 for binary adsorption). Therefore, *q*_binary_/*q*_single_ values should be interpreted as descriptive comparisons rather than isolated quantitative effects of the co-solute.

At the low initial concentration level (5 mg L^−1^), only minor differences were observed between the single- and binary-solute systems. For Cu(ii), the single-solute adsorption capacity (*q*_e,s_) was 2.3948 mg g^−1^, compared with 2.3949 and 2.3884 mg g^−1^ in Cu5/DR20 and Cu5/DR25, corresponding to *q*_binary_/*q*_single_ ratios of 1.0000 and 0.9973, respectively. For DR79, *q*_e,s_ was 2.3223 mg g^−1^, compared with 2.3522 and 2.3313 mg g^−1^ in DR5/Cu20 and DR5/Cu25, corresponding to ratios of 1.0129 and 1.0039, respectively. Thus, only minor deviations from the corresponding single-solute systems were observed at 5 mg L^−1^.

At an intermediate initial concentration of 10 mg L^−1^, the magnitude and direction of the differences varied with the concentration combination. For Cu(ii), the *q*_binary_/*q*_single_ ratio decreased from 0.9936 in Cu10/DR10 to 0.9890 in Cu10/DR20, corresponding to decreases of 0.64% and 1.10%, respectively. In contrast, for DR79, the corresponding ratios were 1.0319 and 1.0172 in DR10/Cu10 and DR10/Cu20, representing increases of 3.19% and 1.72%, respectively. These results indicate that the differences between the single- and binary-solute systems depended on the initial concentration combination rather than following a uniform trend.

At an initial concentration of 20 mg L^−1^, deviations from the corresponding single-solute systems were more pronounced. For Cu(ii), increasing the DR79 concentration from 5 to 10 mg L^−1^ decreased the *q*_binary_/*q*_single_ ratio from 0.9932 to 0.9816, corresponding to decreases of 0.68% and 1.84%, respectively. For DR79, the corresponding *q*_binary_/*q*_single_ ratios were 0.9847 and 0.9820 in the presence of 5 and 10 mg L^−1^ Cu(ii), respectively, corresponding to decreases of 1.53% and 1.80%.

Descriptive comparison of the binary and corresponding single-solute systems showed that the magnitude and direction of the observed differences varied with the initial concentration combination. However, these comparisons should be interpreted cautiously because the experiments were not conducted under fully identical conditions: the single-solute adsorption experiments were performed at pH 2 for DR79 and pH 5 for Cu(ii), whereas the binary experiments were conducted at pH 4. Thus, differences between (*q*_binary_) and (*q*_single_) cannot be attributed solely to the presence of the co-solute, as pH-dependent changes in surface charge, adsorbate speciation, and adsorption-site availability may also contribute. For Cu(ii), the *q*_binary_/*q*_single_ ratios ranged from 0.9816 to 1.0000, corresponding to changes of approximately −1.84% to 0.00%, whereas those for DR79 ranged from 0.9820 to 1.0319, corresponding to changes of approximately −1.80% to +3.19% (Table S6A). One-way ANOVA followed by Tukey's multiple-comparison test showed that the statistical significance of these differences depended on the matched initial-concentration condition, with some binary systems not significantly different from the corresponding single-solute systems and others showing significant differences (Table S6B (SI)). Overall, these results indicate concentration-dependent differences between the single- and binary-solute systems, consistent with non-independent adsorption behaviour rather than a uniform cooperative or competitive effect. Any specific Cu(ii)–DR79 interaction is therefore considered a plausible interpretation rather than a demonstrated molecular mechanism. Within the binary system, where the pH was maintained at 4, the adsorption response varied with the initial concentration combination, indicating concentration-dependent binary adsorption behaviour. These trends can therefore be discussed within the binary system, although they do not by themselves establish a specific Cu(ii)–DR79 interaction mechanism, which remains a plausible but unconfirmed interpretation.

#### 3.3.4. Reusability of the studied material

The results regarding the reusability of BP7003 for the adsorption of the Cu(ii) and DR79 mixture are presented in Fig. 12c. It shows a gradual decline in adsorption performance with repeated use. Cu(ii) removal decreases from 98.86 ± 0.16% in the first cycle to 92.31 ± 0.35% after the fourth cycle. A more pronounced reduction was observed for DR79, for which the removal efficiency declined from 97.48 ± 0.20% to 87.77 ± 0.32% over the same period. The faster decrease observed for DR79 may be related to several factors. Residual dye molecules remaining on the surface after regeneration, changes in adsorption-site accessibility, and other surface alterations accumulated during successive adsorption–desorption cycles could all influence the adsorption behavior. The available data do not allow these possibilities to be distinguished because no post-regeneration characterization was conducted.

After four regeneration cycles, BP7003 still preserved most of its original adsorption performance. Within the scope of the present experiments, the material exhibited satisfactory short-term reusability under the investigated operating conditions.

### 3.4. Proposed adsorption mechanism

The adsorption behaviour of Cu(ii), DR79, and their binary mixture reflects interactions between two chemically distinct solutes and a heterogeneous carbon surface. BP7003 combines a carbonized framework, residual oxygen-containing functionalities, and a heterogeneous pore network that can influence adsorption-domain accessibility. Cu(ii) and DR79 differ substantially in charge, molecular dimensions, and functional groups; their adsorption responses therefore reflect different combinations of electrostatic, surface-chemical, and accessibility effects.

The proposed mechanism integrates zeta-potential and pH-dependent adsorption data with FTIR and XPS analyses BP7003 samples before and after single and mixed adsorption at pH 4 (Fig. 13 and 14), Raman characterization, and the textural properties of BP7003. Collectively, these results constrain changes in electrokinetic behaviour, surface functionality, carbon structure, and the retention of Cu- and S-containing species within the XPS sampling depth. The interpretation is therefore formulated at the surface-chemical level, whereas specific molecular orientations, coordination geometries, and atomically resolved interfacial structures remain unresolved.

**Fig. 13:**
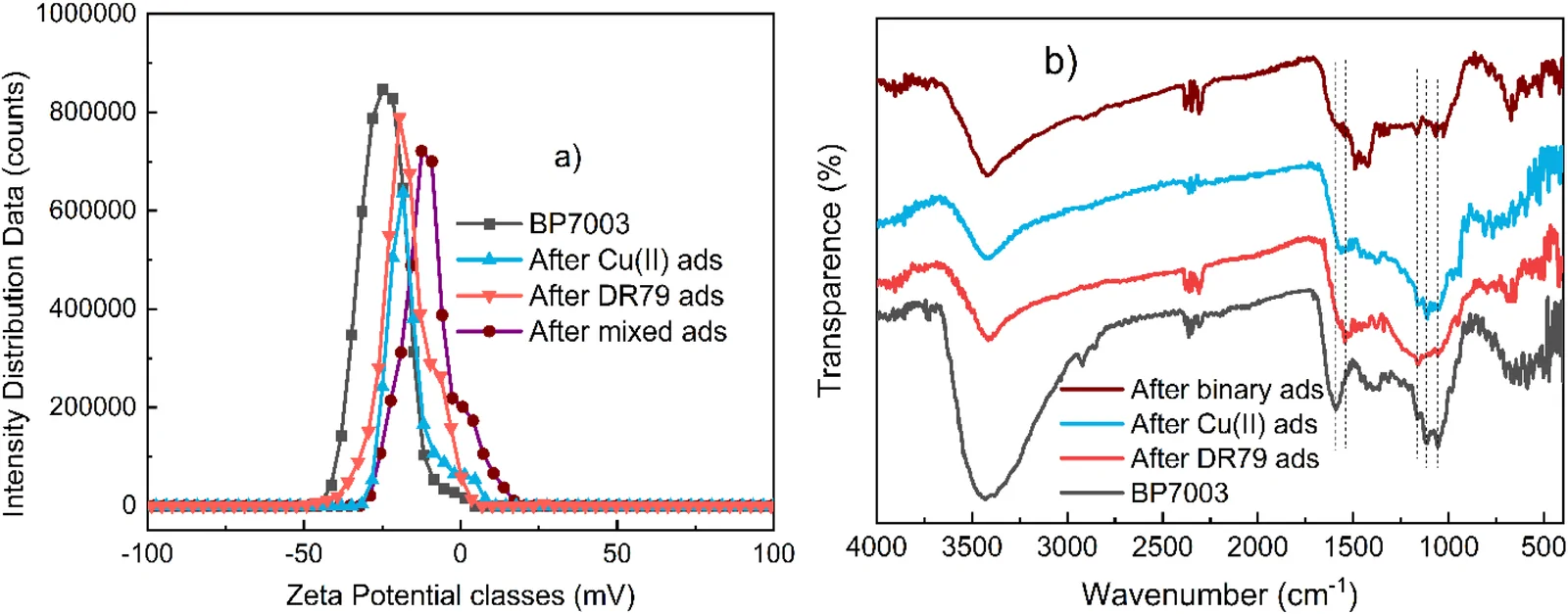
Zeta potential (a) and FTIR spectrum (b) of BP7003 before and after adsorption.

**Fig. 14:**
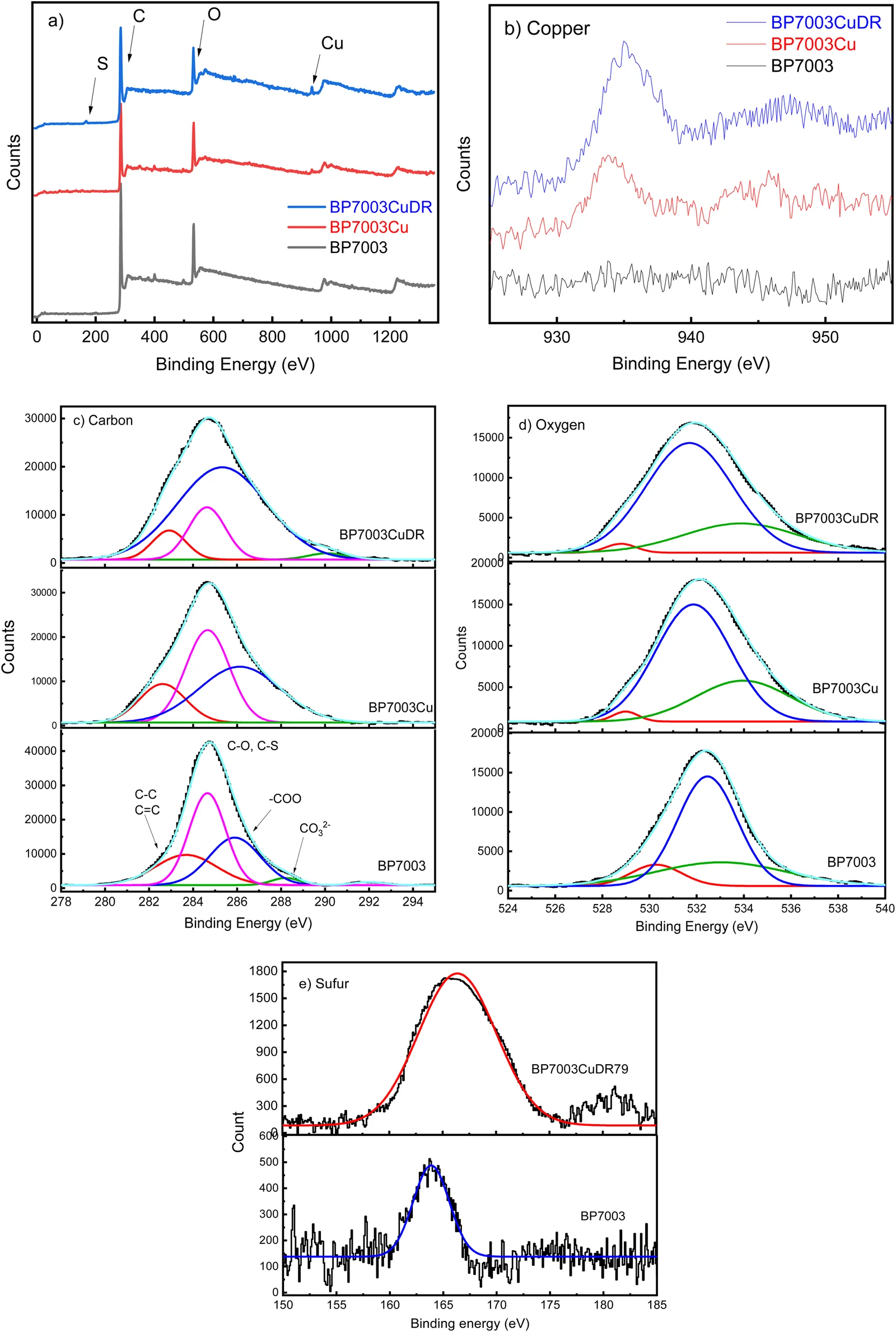
XPS spectra of BP7003 before and after adsorption: (a) survey, (b) Cu 2p, (c) C 1s, (d) O 1s, and (e) S 2p.

#### 3.4.1. Cu(ii) adsorption

Cu(ii) uptake is influenced by both the interfacial electrokinetic environment and surface chemistry of BP7003. At pH 4, pristine BP7003 exhibits a negative zeta potential (Fig. 13a), indicating a negative electrokinetic potential at the slipping plane that is consistent with electrostatic attraction toward cationic Cu species. Following Cu(ii) adsorption under the same pH condition, the zeta potential becomes less negative, indicating a modification of the interfacial electrokinetic environment accompanying Cu retention. This shift is consistent with partial compensation and/or screening of the negative electrokinetic potential following Cu(ii) retention.

Separately, Cu(ii) adsorption increases with increasing pH over the investigated range (Fig. 8). This pH dependence is consistent with possible changes in the protonation state of oxygen-containing surface functionalities and their interaction with dissolved Cu species. However, because zeta potential was measured only at pH 4, the evolution of the electrokinetic potential with pH was not experimentally determined and is therefore not inferred directly from the zeta-potential data. Taken together, the pH-dependent adsorption response and the zeta-potential change observed at pH 4 are consistent with possible contributions from the interfacial electrokinetic environment and oxygen-containing surface functionalities. These observations do not, however, distinguish nonspecific electrostatic association from specific inner-sphere coordination.

The kinetic data describe the rate of Cu(ii) transfer from solution to the solid phase. Although the pseudo-second-order model provides a good empirical description of the uptake profile, the fit does not by itself establish chemisorption or Cu–O bond formation. The kinetic analysis is therefore used to characterize uptake-rate behaviour rather than to assign a specific chemical mechanism.

FTIR provides further evidence for modification of oxygen-containing surface environments. Changes in the O–H, C=O, and C–O-related regions following Cu(ii) adsorption (Fig. 13b) indicate perturbation of surface functionalities during uptake. Hydroxyl-, carbonyl-, carboxyl-, and related oxygen-containing groups provide chemically plausible environments for interaction with dissolved Cu species.^58,62^ In combination with the pH and zeta-potential responses, these spectral changes support their involvement in Cu(ii) uptake, although FTIR does not uniquely identify the individual functional group or coordination mode responsible.

The equilibrium data provide an additional constraint. The Redlich–Peterson and Temkin models describe the adsorption data well, consistent with a distribution of energetically non-equivalent adsorption environments. Such heterogeneity is reasonable for pyrolyzed biomass-derived carbon containing chemically distinct oxygenated sites, carbon domains, structural disorder, and pores with different accessibility. The isotherm models support heterogeneous adsorption environments but do not establish their molecular origin.

Colletively, the Cu(ii) results support a heterogeneous surface-association process involving the electrokinetic environment and oxygen-containing surface functionalities, with the equilibrium data indicating a distribution of adsorption environments. The present evidence does not permit quantitative separation of electrostatic association from specific surface coordination.

#### 3.4.2. DR79 adsorption

DR79 interacts with BP7003 through a different combination of molecular and interfacial properties. The dye contains a sulfonate functionality together with extended aromatic and conjugated domains, whereas BP7003 provides carbon-rich domains, residual polar functionalities, and a heterogeneous pore network. Its adsorption behaviour may therefore reflects contributions from both interfacial chemistry and molecular accessibility.

DR79 uptake decreases with increasing pH (Fig. 8), which is consistent with a possible contribution from pH-dependent interfacial electrostatic interactions. However, the zeta-potential measurements at pH 4 do not establish how the electrokinetic potential of BP7003 varies over the investigated pH range. The observed pH dependence should therefore not be interpreted as direct evidence of a progressive change in interfacial electrokinetic potential. Moreover, measurable uptake persists at higher pH, indicating that electrostatic effects alone cannot account for the overall adsorption behaviour. Additional short-range interactions with the carbon surface therefore remain plausible.

The molecular structure of DR79 provides several chemically plausible additional interaction environments. Its aromatic and conjugated domains may participate in short-range interactions with carbon-rich surface domains, while its sulfonate and other polar functionalities may interact with oxygen-containing groups on BP7003.^17,20^ These possibilities remain mechanistically plausible, but the present measurements do not establish the orientation or specific interaction geometry of DR79 at the surface.

The FTIR spectra show changes in the aromatic C=C region and in bands associated with oxygen-containing functionalities following DR79 adsorption (Fig. 13b). These changes indicate perturbation of the interfacial chemical environment and are consistent with involvement of the conjugated dye structure and polar surface functionalities. They do not, however, uniquely assign the changes to π-associated interactions or hydrogen bonding.

The Raman results provide structural context for the carbon framework. The *I*_D_/*I*_G_ ratio increases from 0.31 for BP3001 to 0.79 for BP7003, accompanied by progressive loss of aliphatic functionalities during pyrolysis. This evolution indicates substantial restructuring of the biomass-derived carbon matrix and changes in defect-associated carbon environments. The BP3001–BP7003 comparison therefore establishes a clear pyrolysis-induced transformation of the carbon structure. Importantly, the increase in *I*_D_/*I*_G_ is interpreted here as evidence of carbon-structural evolution, rather than as direct evidence for π-associated adsorption of DR79.

The textural properties impose an additional physical constraint on solute accessibility. BP7003 exhibits mesoporous characteristics and a heterogeneous pore network that can influence solute accessibility. Cu(ii), as a small aqueous species, is less susceptible to steric exclusion than the substantially larger DR79 molecule. DR79 adsorption may therefore be more sensitive to the fraction of the pore network accessible to the dye and to associated mass-transfer limitations. The BET-derived properties provide the principal evidence for this interpretation, while SEM observations provide morphological support for the heterogeneous porous structure. Neither technique establishes the location of individual DR79 molecules within specific pores.

Overall, DR79 adsorption is consistent with a combined contribution from electrostatic effects, short-range surface interactions, and molecular accessibility within the heterogeneous carbon structure. The present dataset does not permit quantitative separation of these contributions.

#### 3.4.3. Binary Cu(ii)–DR79 adsorption

The binary system introduces additional complexity because Cu(ii) and DR79 interact simultaneously with the same heterogeneous surface. Their coexistence can modify the local interfacial environment and the availability of adsorption domains; consequently, binary adsorption cannot necessarily be represented as the simple sum of the corresponding single-solute processes.

When Cu(ii) and DR79 coexist, the interfacial environment becomes more complex. The zeta potential approaches near-neutral values, consistent with substantial charge compensation under the co-adsorption conditions. The FTIR spectra of the binary system show broader bands and more pronounced attenuation of oxygen-containing functionalities than those observed for the individual systems, consistent with perturbation of the surface chemical environment during co-adsorption. Together, these results indicate changes in the interfacial environment but do not directly resolve the molecular configuration of the co-adsorbed species.

A plausible hypothesis is that Cu(ii) first associates with oxygen-containing surface functionalities that may carry negative charge under the adsorption conditions and that surface-associated Cu(ii) subsequently influences DR79 adsorption. Such an association could involve electrostatic and/or surface–complexation interactions and may contribute to the concentration-dependent adsorption response observed within the binary system. However, the present data do not directly demonstrate the formation of a Cu–DR79 bridge. In particular, the available spectroscopic evidence indicates changes in the interfacial environment but does not provide atom-resolved identification of a Cu–DR79 coordination structure. The available UV-Vis measurements likewise did not provide clear evidence for a distinct Cu–DR79 complex in solution. Therefore, Cu(ii)-mediated bridging should be regarded as a plausible but unconfirmed interfacial hypothesis rather than an established adsorption mechanism. Other surface and transport effects may also contribute to the observed binary adsorption behaviour.

The concentration dependence further constrains this interpretation. Under binary conditions where higher DR79 uptake was observed relative to the corresponding single-solute reference, Cu-associated modification of accessible surface environments may have contributed to DR79 association. With increasing adsorption loading, greater occupation of accessible domains can increase competition for favourable adsorption environments. The larger molecular dimensions of DR79 may further increase its sensitivity to steric and diffusional constraints. The binary response is therefore better described as a concentration-dependent balance among surface modification, adsorbate association, and competition, rather than as a single cooperative mechanism operating uniformly across the investigated range.

FTIR provides complementary evidence for a modified interfacial environment during co-adsorption. Changes in the oxygen-containing and aromatic regions differ from those observed for the corresponding single-solute systems (Fig. 13b), indicating that simultaneous adsorption of Cu(ii) and DR79 alters the surface chemical environment. These changes are compatible with coupling among surface functionalities and the two adsorbates, but they do not resolve a specific ternary molecular configuration.

The enhanced DR79 uptake should therefore not be designated as positive cooperativity solely on the basis of the binary adsorption data. Such a designation would require a defined non-interacting reference and a quantitative criterion demonstrating positive deviation from independently expected uptake. The present results are more appropriately described as interaction-assisted surface association, with competition and accessibility constraints becoming increasingly relevant at higher adsorption loading.

#### 3.4.4. XPS evidence for surface interactions

XPS provides surface-sensitive evidence for changes in elemental composition and chemical environments following adsorption (Fig. 14). Pristine BP7003 showed no appreciable Cu 2p signal, whereas Cu 2p features emerged after Cu(ii) adsorption in both the single-solute and binary systems, confirming the presence of Cu-containing species within the XPS sampling depth. Differences in the Cu 2p response between the single-solute and binary systems may reflect changes in the abundance and/or local chemical environment of surface-associated Cu species. However, because the Cu 2p spectra were not subjected to component-specific fitting, these differences are not used to assign a specific Cu oxidation state, bonding configuration, or coordination geometry.

The C 1s spectrum of pristine BP7003 was dominated by the carbon framework, with additional contributions from oxygen-containing carbon environments. The principal C–C/C=C component is consistent with the carbonized structure generated during pyrolysis, whereas the higher-binding-energy contributions are assigned more generally to oxygenated or oxidized carbon environments. Following Cu(ii) adsorption, changes in these components accompanied the appearance of Cu, indicating modification of the local carbon–oxygen environment during adsorption. Similar changes in oxygen-containing carbon environments have been reported for biochar-based adsorbents following heavy-metal adsorption, where oxygen-containing surface functionalities may contribute to metal retention.^63^ Co-adsorption produced additional changes in the C 1s envelope together with the appearance of sulfur consistent with DR79-derived species. These changes indicate modification of the surface chemical environment under binary adsorption and support the involvement of the carbon framework and oxygen-containing functionalities, but do not provide molecular-level evidence for a specific aromatic stacking geometry, hydrogen-bond arrangement, or Cu–DR79 coordination structure.

The O 1s spectrum provides complementary evidence for modification of the collective oxygen-containing surface environment following Cu(ii) adsorption. Hydroxyl-, carbonyl-, carboxyl-, and related oxygen-containing species may contribute to overlapping regions of the O 1s envelope; consequently, the fitted components were interpreted collectively rather than assigned to a unique molecular species. Comparable changes in O 1s features have been reported after Cu adsorption on oxygen-functionalized carbonaceous materials and interpreted as consistent with the involvement of oxygen-bearing surface functionalities in related adsorbent systems.^64^ Accordingly, the observed O 1s changes are considered consistent with modification of oxygen-containing surface functionalities during Cu(ii) adsorption, but do not by themselves establish a specific Cu–O coordination structure.

The S 2p response provides XPS evidence for the presence of sulfur-containing species following binary adsorption (Fig. 14e). The binding-energy region of this feature is consistent with an oxidized sulfur environment compatible with the sulfonate functionality of DR79.^65^ The appearance of the S 2p signal supports the presence and retention of sulfur-containing DR79-derived species within the XPS sampling depth. However, the S 2p response alone cannot distinguish among electrostatic, polar, or Cu-associated interactions involving the sulfonate group.

Together, the Cu 2p and S 2p responses support the simultaneous presence of Cu-containing and sulfur-containing species within the XPS sampling depth following binary adsorption, while the C 1s and O 1s responses indicate concomitant changes in the carbon and oxygen environments. Similar Cu-associated interactions involving oxygen-containing surface functionalities and anionic adsorbate groups have been proposed in related adsorption systems.^66^ This literature precedent supports the chemical plausibility of Cu-associated surface modification as one possible contributor to the observed binary behaviour. Accordingly, a Cu-associated modification involving oxygen-containing BP7003 sites and the sulfonate-bearing DR79 remains a plausible working hypothesis rather than an experimentally resolved interfacial structure.

The C 1s and O 1s spectra were fitted using a Shirley background and Gaussian line shape, with charge referencing to the C 1s peak at 284.8 eV. The fitted binding energies, component assignments, full widths at half maximum (FWHM), and relative peak areas are summarized in Table S7 (SI). The Cu 2p and S 2p spectra were evaluated qualitatively and were not subjected to component-specific fitting. Accordingly, the XPS results are interpreted as evidence for changes in the surface chemical environment and retention of Cu- and DR79-derived species rather than as direct proof of a specific molecular bonding configuration.

#### 3.4.5. Integrated mechanistic interpretation

The combined evidence indicates that Cu(ii) and DR79 respond to different but partially overlapping features of BP7003. Cu(ii) uptake is associated mainly with the electrokinetic environment and oxygen-containing surface functionalities, whereas DR79 additionally reflects carbon structure, molecular characteristics, and accessibility within the heterogeneous pore network. The BP3001–BP7003 Raman comparison establishes pyrolysis-induced restructuring of the carbon matrix, while the BET-derived properties provide the physical basis for differences in solute accessibility.

Co-adsorption couples these processes through modification of the local interfacial environment and competition for accessible adsorption domains. The resulting non-independent binary behaviour may arise from Cu-associated surface modification, neighbouring surface-site effects, electrostatic redistribution, or a combination of these processes. At higher adsorption loading, increasing occupation of accessible domains and the larger dimensions of DR79 provide plausible constraints on access to favourable adsorption environments.

The resulting surface-chemical model comprises three experimentally constrained features: (i) Cu(ii) uptake involves the electrokinetic environment and oxygen-containing surface functionalities, with equilibrium behaviour indicating heterogeneous adsorption environments; (ii) DR79 uptake reflects the combined influence of surface charge, chemically plausible short-range interactions with carbon-rich and polar surface domains, and molecular accessibility; and (iii) binary adsorption couples these processes through modification of the local interfacial environment and competition for accessible adsorption domains.

The Cu-associated modification involving oxygen-containing BP7003 sites and the sulfonate-bearing DR79, illustrated schematically in Fig. 15, should therefore be presented explicitly as a working mechanistic hypothesis, rather than as an atomically resolved Cu–DR79 structure. The present FTIR, Raman, XPS, zeta-potential, and textural measurements establish changes in surface chemistry and adsorption behaviour but do not directly determine the coordination geometry or molecular arrangement of the interfacial species.

**Fig. 15:**
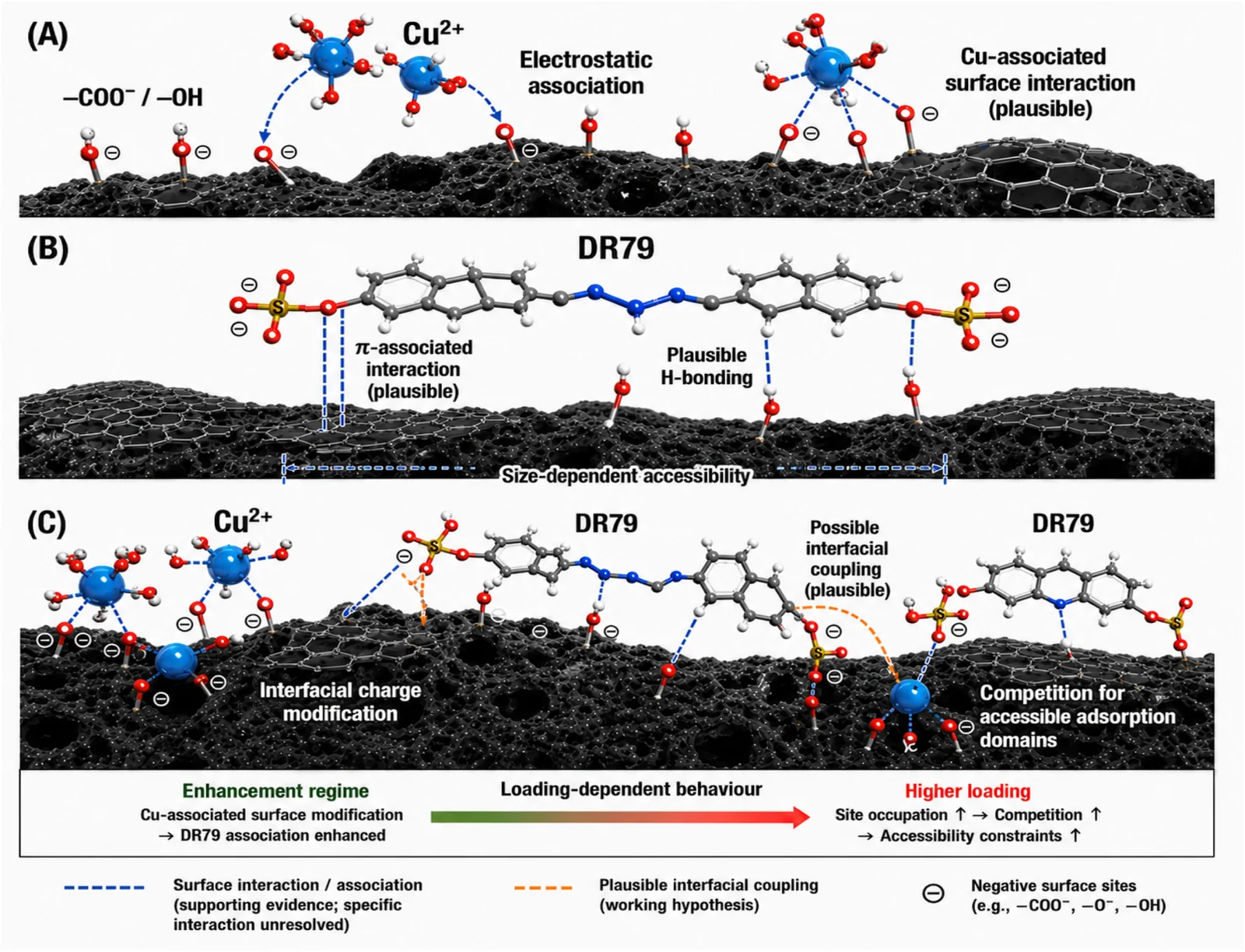
Schematic of the proposed adsorption mechanisms of Cu^2+^ and DR79 on BP7003: (A) Cu^2+^–surface association; (B) DR79–surface interactions; and (C) proposed interfacial effects and competitive adsorption in the binary system. Dashed lines indicate proposed interactions and hypothetical interfacial coupling.

Overall, the dataset supports a surface-chemical model of concentration-dependent binary adsorption, in which electrostatic effects, surface functionality, carbon structure, molecular accessibility, and adsorbate–surface coupling collectively influence the observed behaviour. Definitive identification of Cu coordination geometry or a direct Cu-mediated linkage between BP7003 and DR79 would require coordination-sensitive measurements beyond the present dataset.

### 3.5. Environmental application, limitations, and future perspectives

#### 3.5.1. Environmental application

BP7003 functions as a dual-purpose adsorbent for systems containing both metal ions and organic dyes. Its structure combines accessible pores with chemically active sites, allowing interactions with species of different size and charge. This feature fits mixed effluents such as textile wastewater.

The material originates from black bean pods, a low-value agricultural residue. The conversion route is simple and does not rely on chemical activation. The conversion route avoids chemical activation steps commonly used for some engineered adsorbents; however, the overall environmental and economic performance requires further assessment. Cu(ii) uptake remains competitive among pristine biochars, while DR79 removal is comparable to other unmodified carbons. The pore system includes both micro- and mesopores. Small hydrated ions and bulky dye molecules access different domains, which supports co-removal in mixed solutions. At low concentrations, Cu^2+^ may assist dye uptake through local charge effects or surface association. At higher loadings, site competition becomes dominant. This pattern suggests use under moderate contamination levels or as a polishing step after primary treatment. Reusability was retained over several cycles, although a gradual decline was observed, particularly for DR79. The regeneration results demonstrate short-term recovery of adsorption performance under the investigated laboratory conditions. However, the relatively intensive regeneration protocol and its associated solvent, chemical, washing, and waste-handling requirements limit the extent to which practical reusability can be assessed from the present data. The overall environmental and economic performance therefore requires further assessment.

#### 3.5.2. Limitations and future perspectives

Although BP7003 showed promising performance in binary Cu(ii)–DR79 adsorption, its practical potential remains subject to several operational and mechanistic limitations.

The present experiments were conducted in controlled aqueous solutions and batch mode, whereas real wastewater contains competing ions, organic matter, and variable pH that may affect adsorption behaviour. The adsorption capacity of BP7003 toward DR79 (*q*_m_ ≈ 66.05 mg g^−1^) was lower than that observed for Cu(ii) under the investigated conditions, and a gradual decline in removal efficiency was observed over four adsorption–desorption cycles, particularly for DR79. In addition, the regeneration procedure required repeated washing with 70% ethanol followed by HCl treatment, involving additional solvent and chemical consumption and associated waste-handling requirements. The cumulative eluent volume, number of repeated washing steps, desorption recovery, and sorbent mass recovery were not quantified in the present study. Consequently, the four-cycle experiment demonstrates short-term reusability under the investigated laboratory conditions, but does not establish desorption recovery, regeneration efficiency at the process scale, long-term stability, or practical or economic feasibility. Optimization of eluent consumption and regeneration efficiency was beyond the scope of the present work.

A limitation of the present study is that the particle-size distributions of the ground biomass and pyrochar were not quantitatively determined; therefore, a numerical particle-size range cannot be reported. The same batch of ground material was used for the comparative adsorption experiments under identical experimental conditions to reduce potential particle-size-related variability. The independent effect of particle size on adsorption behaviour was not evaluated.

Mechanistically, XPS, FTIR, and zeta-potential measurements provided complementary evidence of changes in the interfacial environment, but the abundance of surface acidic and basic functionalities and the molecular-level configuration of co-adsorbed species were not quantitatively resolved. Future studies should therefore focus on: (1) optimizing regeneration to reduce solvent and acid consumption; (2) evaluating continuous-flow operation and mass-transfer behaviour; (3) validating co-adsorption using real wastewater and representative background matrices; and (4) combining DFT/molecular dynamics with EXAFS/XANES to further resolve plausible interfacial configurations and the local coordination environment of surface-associated Cu species, while assessing whether mild surface modification can improve DR79 uptake without compromising regeneration performance.

## 4. Conclusion

Pyrochar derived from black bean pods under controlled pyrolysis conditions (300–700 °C; 1–5 h) exhibited progressive carbonization and development of a heterogeneous porous carbon framework. BP7003, prepared at 700 °C for 3 h, exhibited a high specific surface area (∼517 m^2^ g^−1^), a substantial microporous contribution supported by low-pressure N_2_ adsorption and t-plot analysis, and complementary mesopore-range features. Cu(ii) exhibited rapid uptake and a maximum experimentally determined equilibrium uptake of 117.13 ± 0.95 mg g^−1^ at pH 5, with adsorption influenced by the electrokinetic environment and oxygen-containing surface functionalities, whereas DR79 exhibited a lower experimental equilibrium uptake of 66.05 ± 1.15 mg g^−1^ and greater dependence on pore accessibility. The available evidence does not permit quantitative separation of electrostatic association from specific surface coordination or resolution of a specific DR79 interaction geometry.

Binary adsorption exhibited concentration-dependent behaviour under the fixed binary experimental condition of pH 4. The differences from the corresponding single-solute systems were generally small and varied with the initial concentration combination. Because the single-solute reference experiments were conducted at different pH values, these deviations cannot be uniquely attributed to Cu(ii)–DR79 interactions or interpreted as direct evidence of competition or cooperation. The higher-loading suppression is compatible with increased competition for accessible adsorption domains, whereas the higher DR79 uptake observed under some lower-loading conditions may reflect altered interfacial environments. These interpretations remain provisional, and experiments under strictly matched pH and concentration conditions are required to quantitatively resolve the contribution of co-solute interactions.

BP7003 retained most of its adsorption performance after four regeneration cycles under the investigated laboratory conditions, although a greater decline was observed for DR79. The origin of this decline remains unresolved. Overall, BP7003 exhibited adsorbate- and concentration-dependent behaviour, reflecting the combined influence of surface chemistry, interfacial electrostatics, and pore accessibility. These findings support further evaluation of BP7003 for mixed-contaminant adsorption, particularly under realistic water matrices and continuous-flow conditions. Longer-term regeneration studies and coordination-sensitive measurements are needed to assess operational stability and further resolve the basis of possible Cu-associated interfacial coupling.

## Author contributions

Ngoc Anh Hoang: resources, investigation, data curation, formal analysis, writing – original draft; Thi Thuy Ha Dinh: investigation, data curation, formal analysis; Thuong Tuan Nguyen: resources, software, visualization; Truong Xuan Vuong and Tien Duc Pham: supervision, writing – review & editing, validation; Truong Thi Truong: visualization, validation, supervision, methodology, data curation, conceptualization, project administration, writing – original draft, writing – review & editing. All authors read and approved the final manuscript.

## Conflicts of interest

The authors declare that they have no known competing financial interests or personal relationships that could have appeared to influence the work reported in this paper.

## Supplementary Material

RA-016-D6RA07689K-s001

## Data Availability

The data supporting this article are provided in the supplementary information (SI). Supplementary information: experimental datasets, calibration results, statistical analyses, and selected material characterization results. The kinetic datasets used for model fitting are reported in Table S4, the equilibrium datasets used for adsorption isotherm modelling in Table S5A, and the corresponding model-fit statistics in Table S5B. Quantitative comparisons of single- and binary-solute adsorption and their associated statistical analyses are provided in Tables S6A and S6B, respectively. Additional supporting results are presented in the remaining tables and figures. See DOI: https://doi.org/10.1039/d6ra07689k.
