# Supplementary material for: Black bean pod-derived pyrochar for single and binary adsorption of Cu(ii) and direct red 79: adsorption performance, interfacial interactions and regeneration
Source: RSC Adv. 2026 Oct 29;16(49):67006–33. doi: 10.1039/d6ra07689k (PMC13717515; doi:10.1039/d6ra07689k)
Supplement: Supplementary file 1 [file RA-016-D6RA07689K-s001.pdf]

Supplementary Information (SI) for RSC Advances.
This journal is © The Royal Society of Chemistry

SUPPLEMENTARY INFORMATION

Title: Black bean pod-derived pyrochar for single and binary adsorption of Cu(II) and Direct Red 79: Adsorption performance, interfacial interactions and regeneration

Table S1. Pyrolysis conditions, pyrochar masses, and mass yields from three independent preparations.

Sample	Temperature (°C)	Residence time (h)	Mass of biomass (g)	Pyrochar mass, Rep. 1 (g)	MY Rep. 1 (%)	Pyrochar mass, Rep. 2 (g)	MY Rep. 2 (%)	Pyrochar mass, Rep. 3 (g)	MY Rep. 3 (%)	MYave (%)	SD (%)	RSD (%)
BP3001	300	1	10.00	2.815	28.15	2.857	28.57	2.849	28.49	**28.40**	0.22	0.79
BP4501	450	1	10.00	2.701	27.01	2.667	26.67	2.691	26.91	**26.86**	0.17	0.65
BP6001	600	1	10.00	2.542	25.42	2.512	25.12	2.533	25.33	**25.29**	0.15	0.61
BP7001	700	1	10.00	2.476	24.76	2.454	24.54	2.462	24.62	**24.64**	0.11	0.45
BP7003	700	3	10.00	2.433	24.33	2.405	24.05	2.421	24.21	**24.20**	0.14	0.58
BP7005	700	5	10.00	2.408	24.08	2.384	23.84	2.401	24.01	**23.98**	0.12	0.51

***Note**: MY was calculated as ($m_{biochar}/m_{biomass}$*100%). Values are means of three independent preparations (n =3); SD and RSD denote standard deviation and relative standard deviation, respectively.*

Table S2. Calibration characteristics, limits of detection (LOD), and limits of quantification (LOQ) for DR79 and Cu(II) determination under the investigated conditions.

Analyte	Matrix/condition	λ (nm)	Linear range (mg L$^{⁻1}$)	Slope	Intercept	R^2	Blank SD (n = 6)	LOD (mg L$^{⁻1}$)	LOQ (mg L$^{⁻1}$)
DR79	pH 2	501	0–30	0.02118	0.00476	0.9982	0.0001412	0.0220	0.0668
DR79	pH 4	501	0–30	0.02172	0.01169	0.9987	0.0001632	0.0248	0.0752
DR79	Aqueous matrix	496	0–20	0.02097	0.01014	0.9979	0.0006971	0.1097	0.3323
DR79	Sulfate-containing matrix	496	0–20	0.02034	0.01128	0.9982	0.0001633	0.0265	0.0803
**Cu(II)**	Aqueous	324.8	0.1–10	0.013706	0.001453	0.9998	0.0001030	0.0248	0.0752

Table S3. Dilution of experimental solutions

adsorbate	Co	dilution factor	The solution after adsorption	
			C_o of solution before adsorption	dilution factor
DR79	50	10		
	100	100	100	10
	200	100	200	10
	500	100	500	100
Cu(II)	20	10	C_o= 20 mg L^{-1} after adsorption for 5 to 30 min	10
	30	10		
	50	10		
	100	100	100	10
	200	100	200	10
	300	100	300	10
	400	100	400	100

Table S4. Experimental conditions and kinetic data used for model fitting of single-solute and binary adsorption.

t (min)	single adsorption				t (min)	Simultaneous adsorption			
	DR79, pH 2, C_o=28.703 mg L^{-1}		Cu(II), pH 5, C_o = 19.328 mg L^{-1}			DR79, pH 4, C_o = 9.6904 mg L^{-1}		Cu(II), pH 4, C_o = 19.4240 mg L^{-1}	
	$q_{t, ave}$	Standard Deviation (SD)	$q_{t, ave}$	Standard Deviation (SD)		$q_{t, ave}$	Standard Deviation (SD)	$q_{t, ave}$	Standard Deviation (SD)
5	8.02487	0.1579	4.0855	0.0081	5	3.0510	0.0216	7.7794	0.0199
15	9.62653	0.1327	6.9119	0.0124	10	3.5855	0.0348	8.5651	0.0085
30	10.8019	0.1104	8.5011	0.0272	20	4.0117	0.0018	9.0994	0.0110
50	12.0009	0.1283	9.1319	0.0232	30	4.2511	0.0332	9.3372	0.0064
80	12.8647	0.1427	9.3502	0.0356	45	4.3919	0.0178	9.3965	0.0062
120	13.4135	0.1044	9.4886	0.0263	60	4.4629	0.0107	9.4872	0.0046
180	13.5783	0.1371	9.5299	0.0191	90	4.5733	0.0055	9.5692	0.0049
					120	4.6581	0.0027	9.6038	0.0031

Table S5A. Experimental equilibrium data used for adsorption isotherm modelling of DR79 and Cu(II) on BP7003.

(A) Experimental equilibrium data:

C₀ (mg L⁻^{1})	DR79: Cₑ (mg L⁻^{1})	DR79: qₑ (mg g⁻^{1})	SD (mg g⁻^{1})	Cu(II): Cₑ (mg L⁻^{1})	Cu(II): qₑ (mg g⁻^{1})	SD (mg g⁻^{1})
9.354028	0.0412	4.66	0.0052	—	—	—
19.019430	0.8538	9.08	0.0138	0.2681	9.53	0.0067
28.703320	3.0664	12.82	0.1284	0.8018	13.86	0.0098
51.082460	8.8147	21.13	0.2180	2.9314	22.61	0.0655
100.681500	27.1370	36.77	0.3483	10.4390	42.93	0.1103
199.879600	88.1890	55.85	0.8531	33.7650	79.42	0.8690
497.473900	365.3800	66.05	1.1465	82.6760	103.13	0.7088
—	—	—	—	150.9600	117.13	0.9450

Note : Single adsorption, DR79, pH 2

DR79 C₀ (mg L⁻^{1})	DR79 Cₑ (mg L⁻^{1})	DR79 qₑ (mg g⁻^{1})	SD (mg g⁻^{1})	Cu(II) C₀ (mg L⁻^{1})	Cu(II) Cₑ (mg L⁻^{1})	Cu(II) qₑ (mg g⁻^{1})	SD (mg g⁻^{1})
9.354028	0.0412	4.66	0.0052	9.585	0.0691	4.76	0.0028
19.019430	0.8538	9.08	0.0138	19.328	0.2681	9.53	0.0067
28.703320	3.0664	12.82	0.1284	28.525	0.8018	13.86	0.0098
51.082460	8.8147	21.13	0.2180	48.152	2.9314	22.61	0.0655
100.681500	27.1370	36.77	0.3483	96.304	10.4390	42.93	0.1103
199.879600	88.1890	55.85	0.8531	192.608	33.7650	79.42	0.8690
497.473900	365.3800	66.05	1.1465	288.912	82.6760	103.12	0.7088
—	—	—	—	385.216	150.960	117.13	0.9450

Note : Single adsorption, Cu(II), pH 5. The equilibrium uptake values were calculated according to $q_e = (C_o - C_e)V/m$, where V/m = 0.5 L g⁻^{1}, corresponding to an adsorbent dosage of 2 g L⁻^{1}. The reported errors represent the experimental uncertainty associated with the equilibrium uptake measurements.

Table S5B. Goodness-of-fit statistics and fitted parameters of the adsorption isotherm models.

Adsorbate	Model	R^2	RMSE (mg g⁻^{1})
DR79	Freundlich	0.955	4.74
DR79	Langmuir	0.976	3.42

Adsorbate	Model	R^2	RMSE ($mg\ g^{-1}$)
DR79	Temkin	0.848	8.68
DR79	Redlich–Peterson	0.981	3.03
DR79	Sips	0.989	2.30
Cu(II)	Freundlich	0.986	4.98
Cu(II)	Langmuir	0.985	5.10
Cu(II)	Temkin	0.891	13.81
Cu(II)	Redlich–Peterson	0.992	3.80
Cu(II)	Sips	0.996	2.53

Table S6A. Quantitative comparison of single- and binary-solute adsorption under matched concentration conditions.

Adsorbate	Single system	Binary system	q_{single} (mg g⁻^{1})	q_{binary} (mg g⁻^{1})	q_{binary}/q_{single}	Change (%)
Cu(II)	Cu5	Cu5 + DR20	2.3948 ± 0.0011	2.3949 ± 0.0018	1.00004	+0.004
Cu(II)	Cu5	Cu5 + DR25	2.3948 ± 0.0011	2.3884 ± 0.0011	0.99733	−0.267
Cu(II)	Cu10	Cu10 + DR10	4.7477 ± 0.0014	4.7175 ± 0.0091	0.99364	−0.636
Cu(II)	Cu10	Cu10 + DR20	4.7477 ± 0.0014	4.6955 ± 0.0114	0.98901	−1.099
Cu(II)	Cu20	Cu20 + DR5	9.6794 ± 0.0038	9.6140 ± 0.0111	0.99324	−0.676
Cu(II)	Cu20	Cu20 + DR10	9.6794 ± 0.0038	9.5009 ± 0.0109	0.98156	−1.844
DR79	DR5	DR5 + Cu20	2.3223 ± 0.0077	2.3522 ± 0.0059	1.01288	+1.288
DR79	DR5	DR5 + Cu25	2.3223 ± 0.0077	2.3313 ± 0.0058	1.00388	+0.388
DR79	DR10	DR10 + Cu10	4.5758 ± 0.0141	4.7216 ± 0.0164	1.03186	+3.186
DR79	DR10	DR10 + Cu20	4.5758 ± 0.0141	4.6543 ± 0.0126	1.01715	+1.715
DR79	DR20	DR20 + Cu5	8.7600 ± 0.0444	8.6256 ± 0.0204	0.98466	−1.534
DR79	DR20	DR20 + Cu10	8.7600 ± 0.0444	8.6023 ± 0.0086	0.98200	−1.800

Values are presented as mean ± SD (n = 3). The q_{binary}/q_{single} ratio was calculated from the corresponding mean adsorption capacities. Positive and negative changes represent enhancement and inhibition, respectively.

Table S6B. Statistical comparison of single- and binary-solute adsorption under matched concentration conditions.

A. Overall one-way ANOVA

Adsorbate	Matched concentration (mg L^{-1})	F(2,6)	Overall p-value
Cu(II)	5	7.3516	0.024341
Cu(II)	10	9.5955	0.013512
Cu(II)	20	95.3970	2.38 × 10⁻⁵
DR79	5	5.5261	0.043562
DR79	10	25.4969	0.001167
DR79	20	8.8312	0.016303

B. Tukey's multiple-comparison test

Adsorbate	Matched concentration (mg L^{-1})	Comparison	Mean difference (mg g^{-1})	Tukey-adjusted p-value	Significance	Tukey grouping
Cu(II)	5	Cu5 vs Cu5+DR20	−0.0001	0.99854	ns	a, a, b
		Cu5 vs Cu5+DR25	+0.0064	**0.03794**	*	
		Cu5+DR20 vs Cu5+DR25	+0.0065	**0.03567**	*	

Adsorbate	Matched concentration (mg L^{-1})	Comparison	Mean difference (mg g^{-1})	Tukey-adjusted p-value	Significance	Tukey grouping
Cu(II)	10	Cu10 vs Cu10+DR10	+0.0302	0.09899	ns	a, ab, b
		Cu10 vs Cu10+DR20	+0.0522	**0.01127**	*	
		Cu10+DR10 vs Cu10+DR20	+0.0220	0.23615	ns	
Cu(II)	20	Cu20 vs Cu20+DR5	+0.0654	**0.00587**	*	a, b, c
		Cu20 vs Cu20+DR10	+0.1785	**<0.0001**	*	
		Cu20+DR5 vs Cu20+DR10	+0.1131	**0.00032**	*	
DR79	5	DR5 vs DR5+Cu20	−0.0299	**0.04053**	*	a, b, ab
		DR5 vs DR5+Cu25	−0.0090	0.61737	ns	
		DR5+Cu20 vs DR5+Cu25	+0.0209	0.13781	ns	
DR79	10	DR10 vs DR10+Cu10	−0.1458	**0.00093**	*	c, a, b
		DR10 vs DR10+Cu20	−0.0785	**0.02001**	*	
		DR10+Cu10 vs DR10+Cu20	+0.0673	**0.03802**	*	
DR79	20	DR20 vs DR20+Cu5	+0.1344	**0.03690**	*	a, b, b
		DR20 vs DR20+Cu10	+0.1577	**0.01887**	*	
		DR20+Cu5 vs DR20+Cu10	+0.0233	0.83785	ns	

*Values are based on mean ± SD from three independent adsorption experiments ($n = 3$). One-way ANOVA was performed separately for each matched concentration set, followed by Tukey's multiple-comparison test. Mean differences are calculated as the first-listed condition minus the second-listed condition. Tukey-adjusted p-values are reported for pairwise comparisons. Statistical significance was considered at $p < 0.05$; * indicates $p < 0.05$ and ns indicates $p \geq 0.05$. Different lowercase letters indicate significant differences according to Tukey's test.*

Table S7. XPS peak fitting parameters of C 1s and O 1s spectra

Region	Sample	Component	BE (eV)	Assignment	FWHM (eV)	Area *%)
C 1s	BP7003	C1	283.68	low-binding-energy carbon contribution/additional carbon component associated with the heterogeneous carbon matrix	3.29	23.45
		C2	284.65	C–C/C=C carbon framework	1.95	42.26
		C3	285.90	oxygenated carbon, C–O-related	2.73	30.53
		C4	288.21	carboxyl/carboxylate-like, O–C=O-related	1.51	2.54
		C5	291.98	π–π* shake-up / high-BE satellite carbon contributon	1.69	1.22
	BP7003 Cu	C1	282.62		2.46	17.13
		C2	284.66		2.34	38.91
		C3	286.12		4.37	43.70
	BP7003 DR79	C1	282.92		1.81	8.60
		C2	284.64		1.98	16.88
		C3	285.33		4.80	72.46
		C4	290.09		2.02	2.06
O 1s	BP7003	O1	520.29		2.51	10.08
		O2	532.46		2.97	61.29
		O3	533.04		6.44	28.64
	BP7003 Cu	O1	528.98		1.27	1.96
		O2	531.86		3.77	68.16
		O3	533.96		4.73	29.88
	BP7003 CuDR79	O1	528.79		1.51	2.04
		O2	531/69		4.34	72.54
		O3	533.87		5.71	25.42

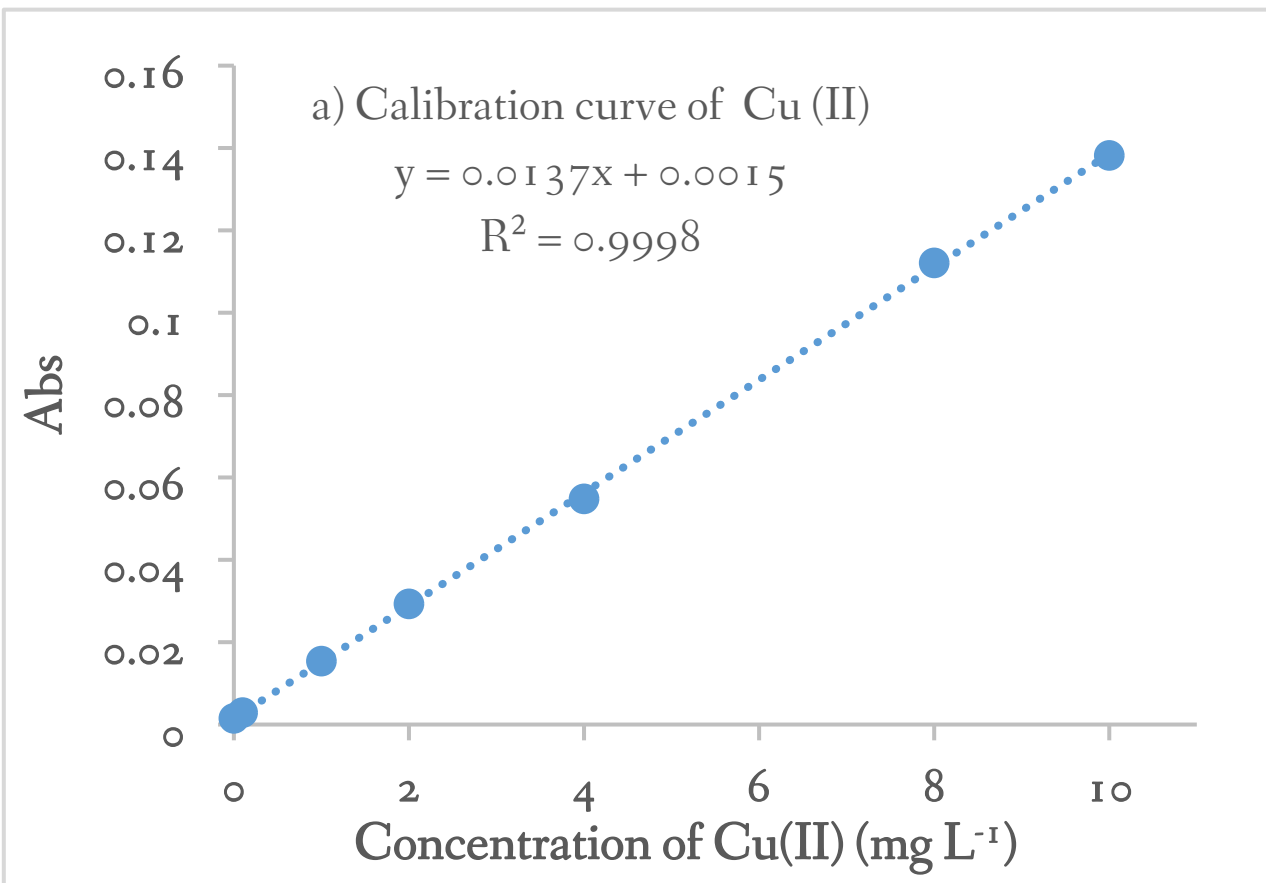
a) Calibration curve of Cu (II)
y = 0.0137x + 0.0015
R² = 0.9998
Abs
Concentration of Cu(II) (mg L-1)
0
0.02
0.04
0.06
0.08
0.1
0.12
0.14
0.16
2
4
6
8
10

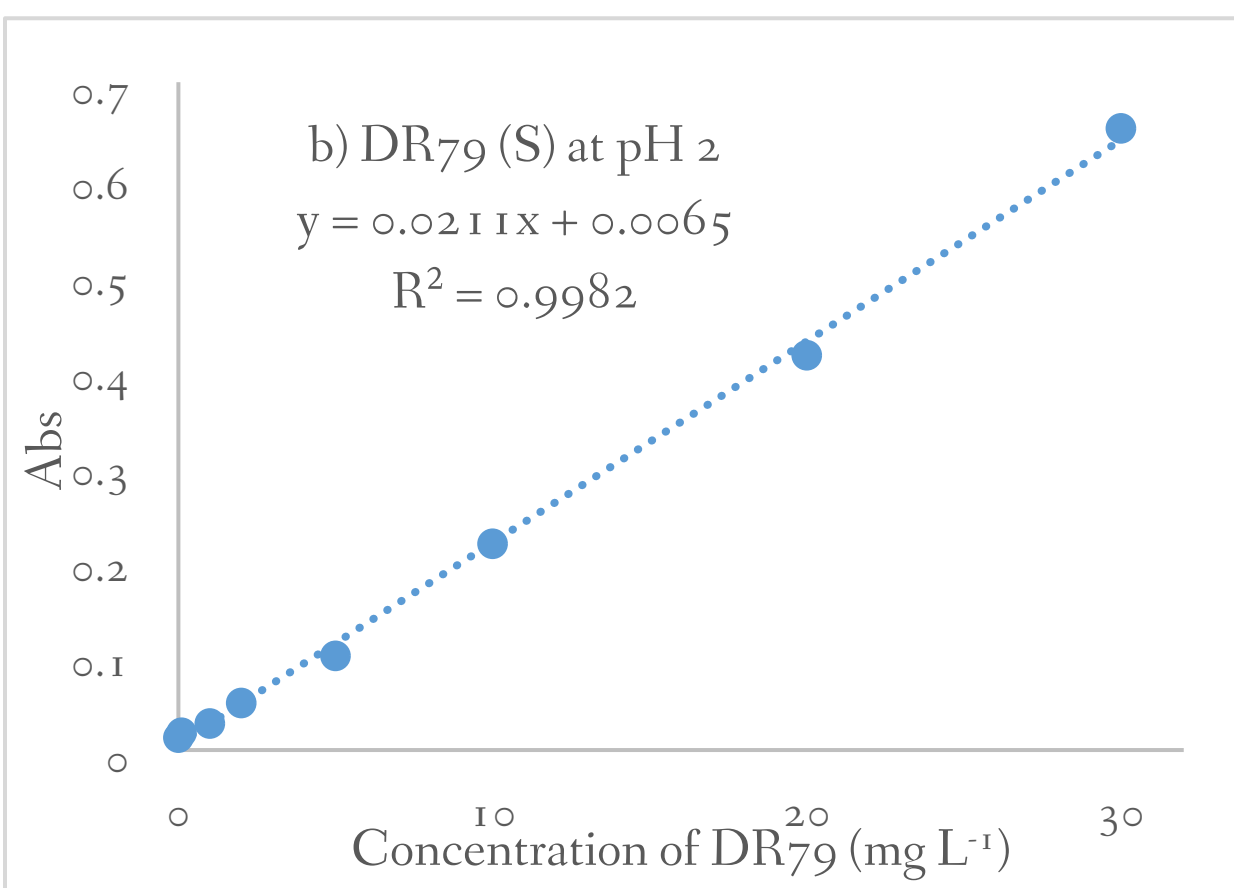
b) DR79 (S) at pH 2
y = 0.0211x + 0.0065
R² = 0.9982
Abs
Concentration of DR79 (mg L-1)
0
0.1
0.2
0.3
0.4
0.5
0.6
0.7
10
20
30

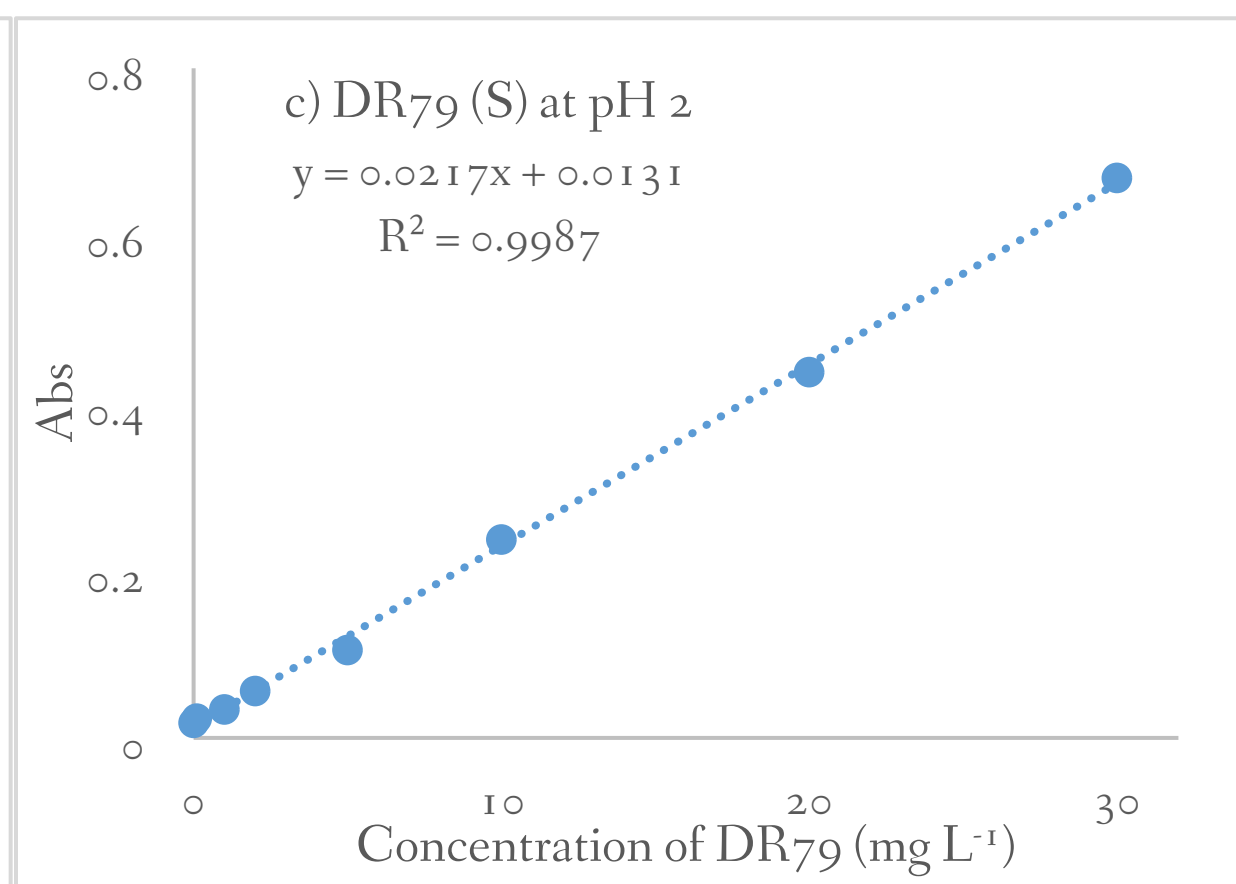
c) DR79 (S) at pH 2
y = 0.0217x + 0.0131
R² = 0.9987
Abs
Concentration of DR79 (mg L-1)
0
0.2
0.4
0.6
0.8
10
20
30

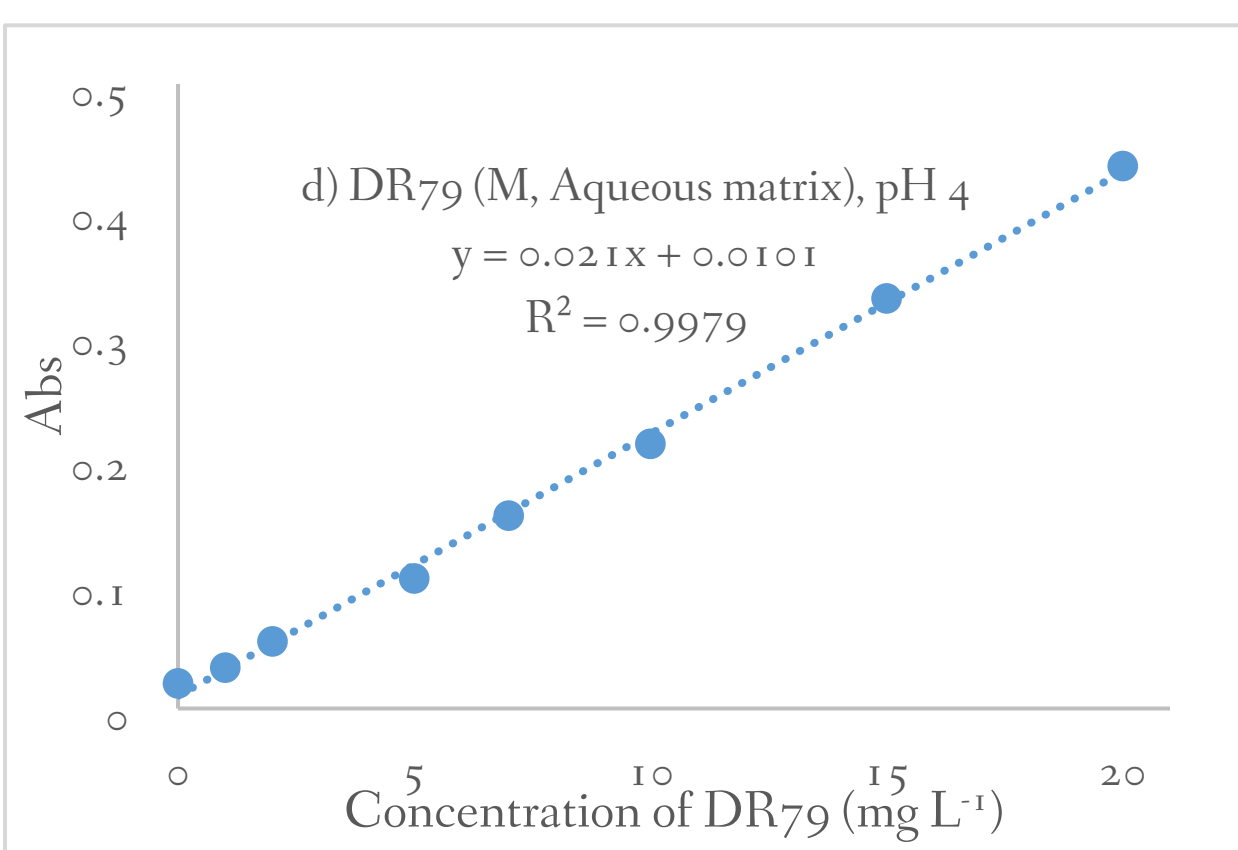
d) DR79 (M, Aqueous matrix), pH 4
y = 0.021x + 0.0101
R² = 0.9979
Abs
Concentration of DR79 (mg L-1)
0
0.1
0.2
0.3
0.4
0.5
5
10
15
20

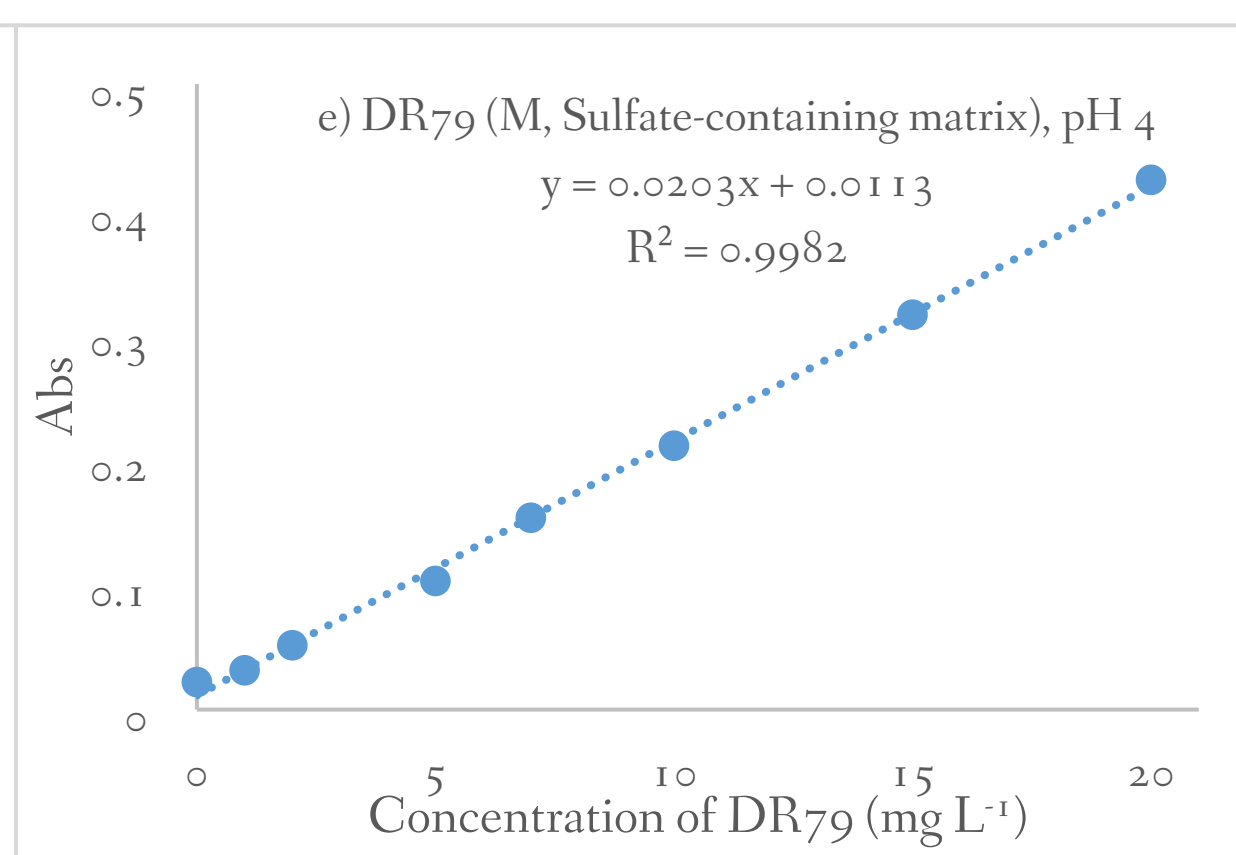
e) DR79 (M, Sulfate-containing matrix), pH 4
y = 0.0203x + 0.0113
R² = 0.9982
Abs
Concentration of DR79 (mg L-1)
0
0.1
0.2
0.3
0.4
0.5
5
10
15
20

Fig. S1. Calibration curves for Cu(II) determination by atomic absorption spectrometry (AAS) (a) and DR79 determination by UV–Vis spectroscopy in DR79 solution at pH 2 (b) and pH 4 (c), in mixtures of DR79 and Cu(II) at pH 4 in aqueous matrix (d) and 0.1 M K_2SO_4 matrix (e).

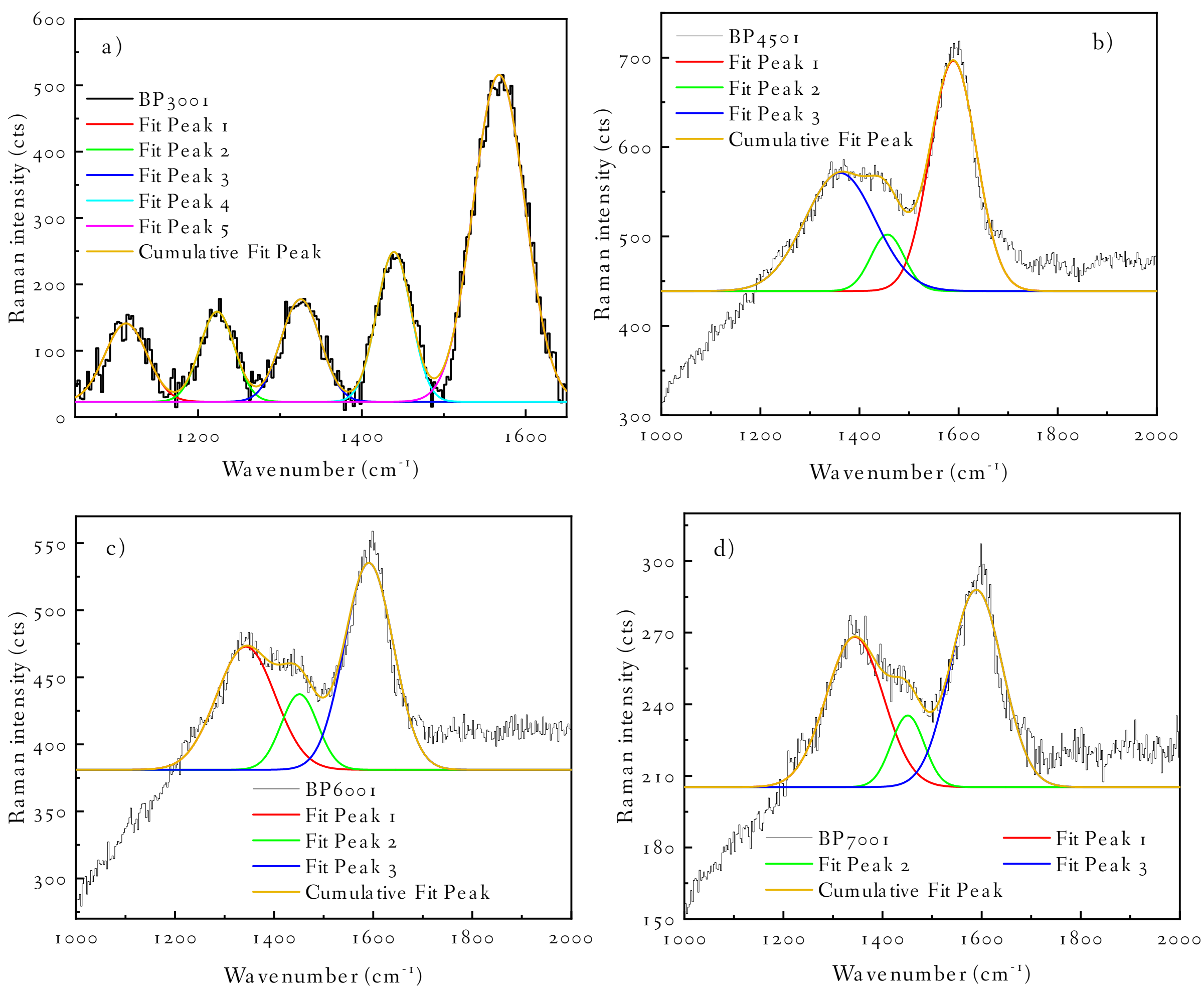

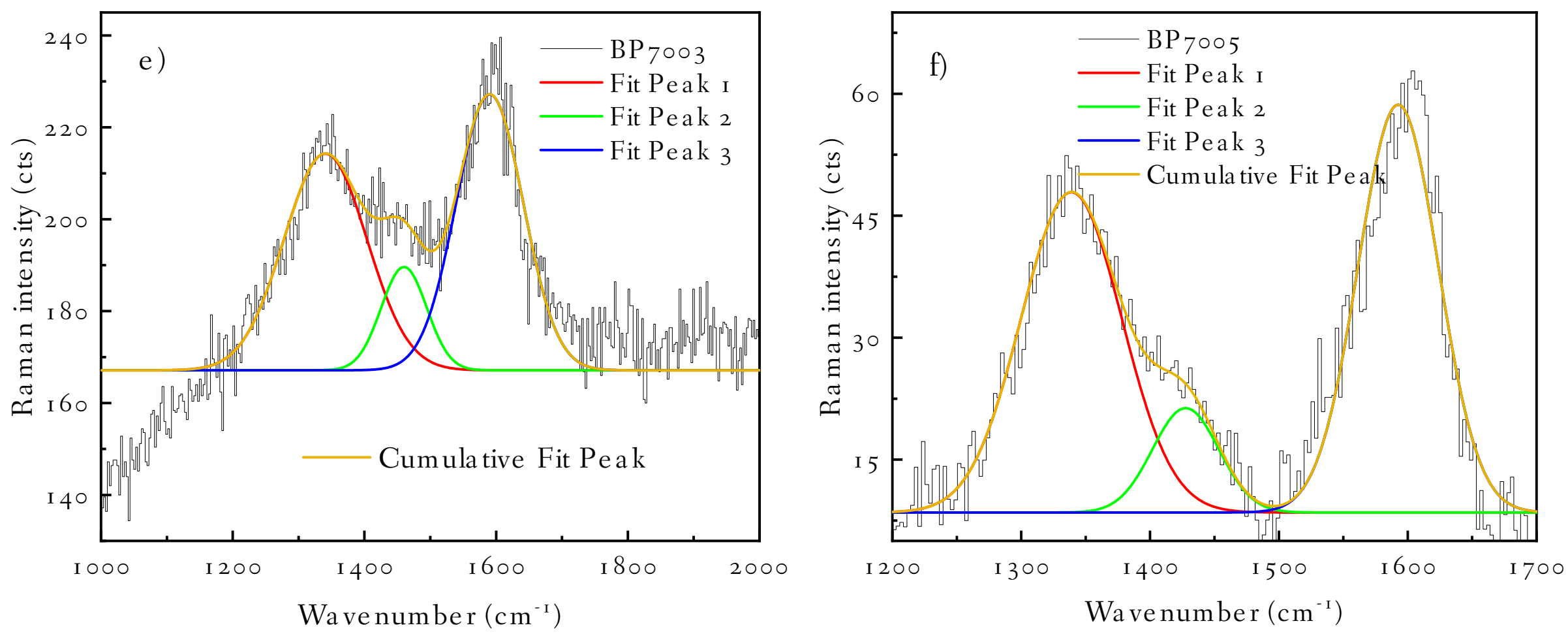

Fig. S2. Fitting Gaussian of Raman spectrum of bean pod pyrochar

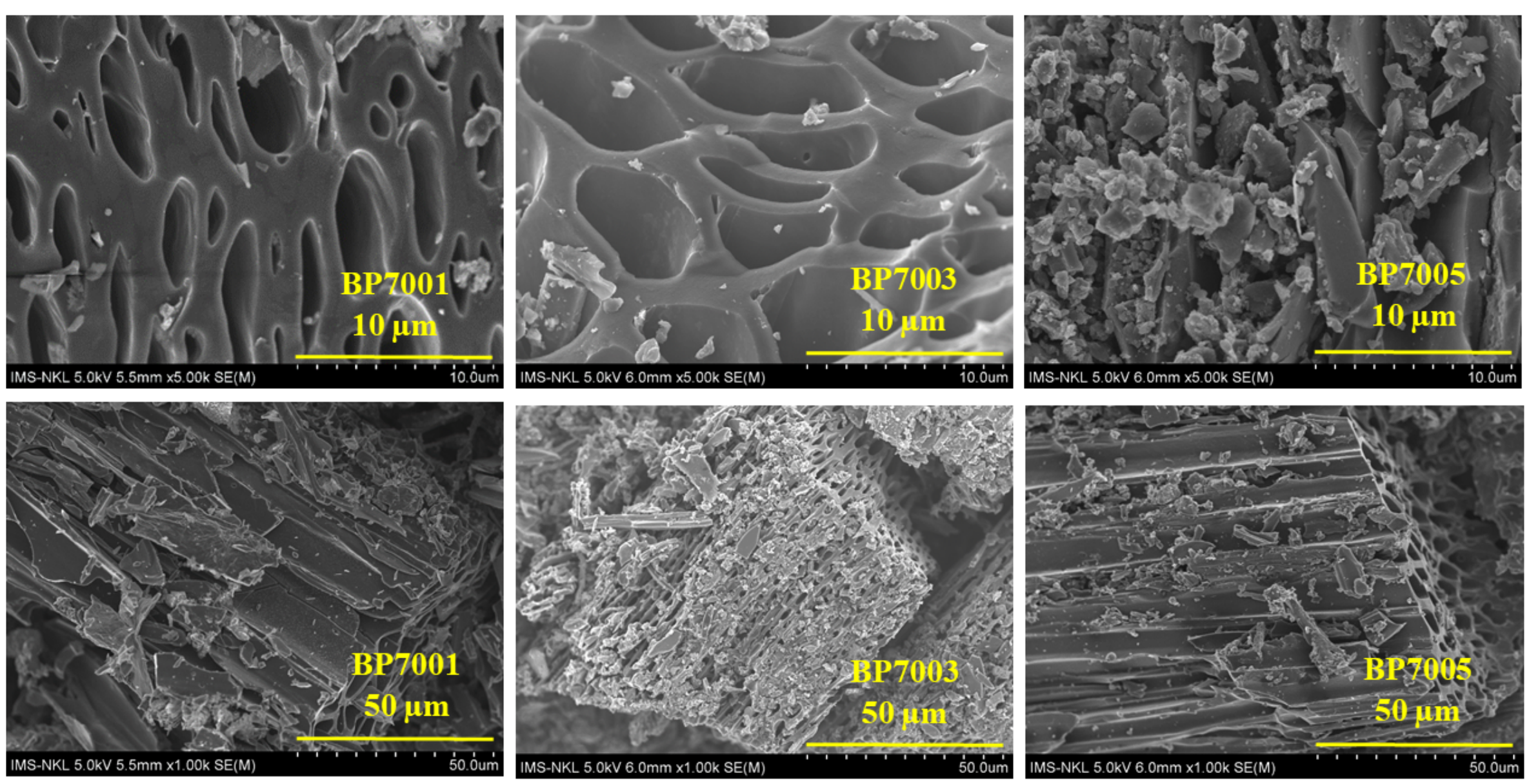

Fig. S3 SEM images at different magnifications of BP700 pyrochars

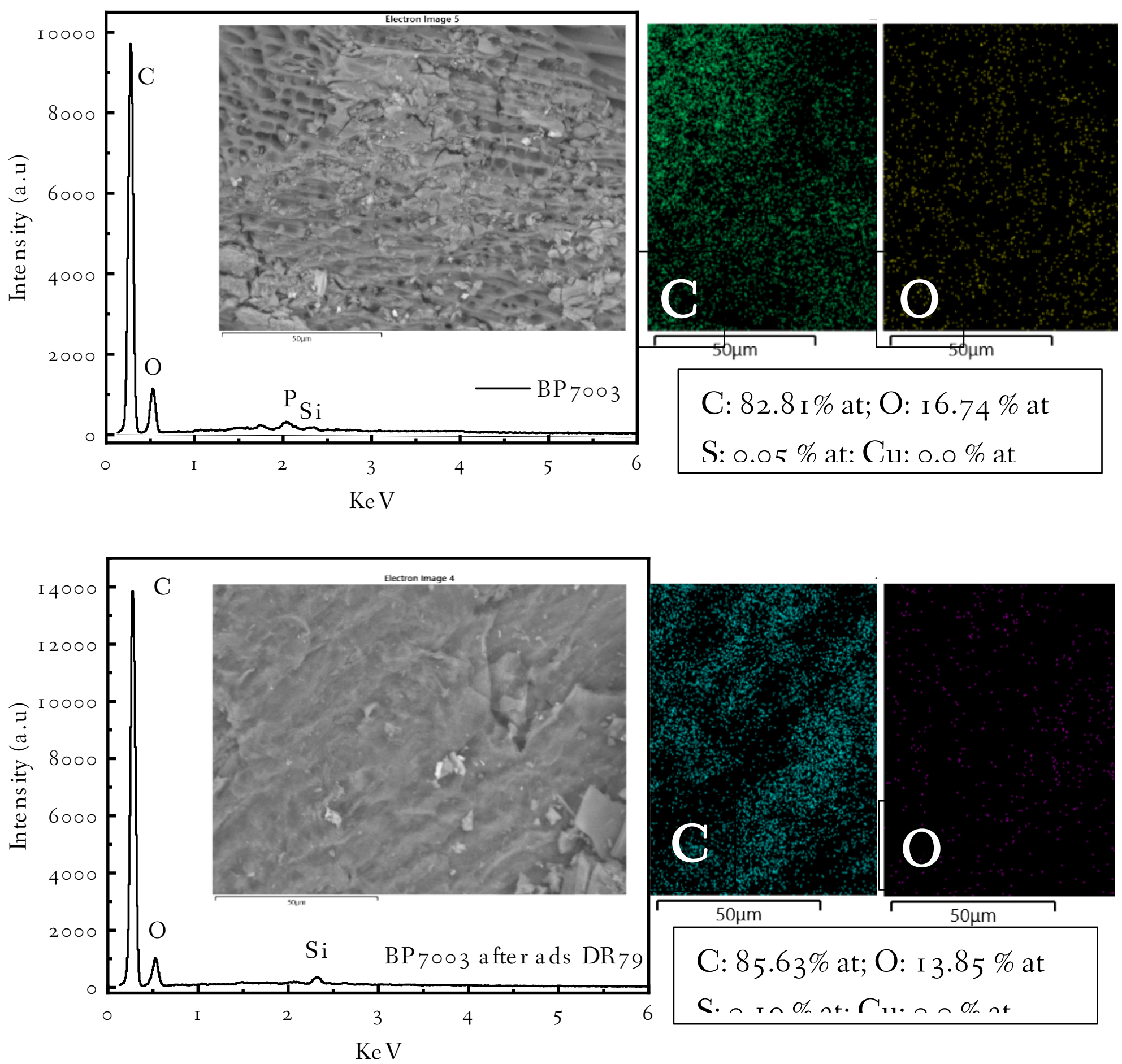

10000
8000
6000
4000
2000
0
Intensity (a.u)
C
O
P
Si
Electron Image 5
50µm
BP7003
0
1
2
3
4
5
6
KeV
C
O
50µm
50µm
C: 82.81% at; O: 16.74 % at
S: 0.05 % at; Cu: 0.0 % at
14000
12000
10000
8000
6000
4000
2000
0
Intensity (a.u)
C
O
Si
Electron Image 4
50µm
BP7003 after ads DR79
0
1
2
3
4
5
6
KeV
C
O
50µm
50µm
C: 85.63% at; O: 13.85 % at

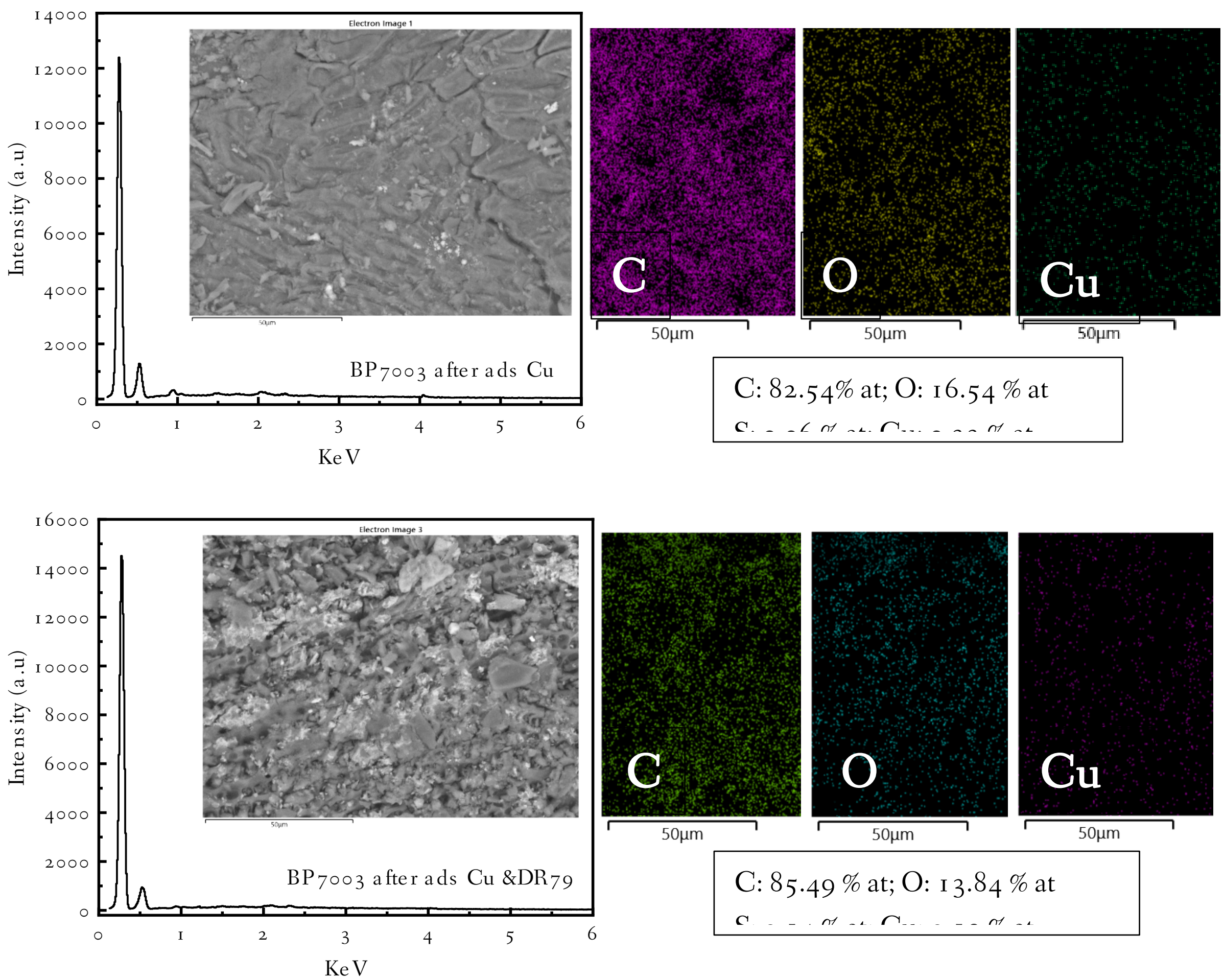

Fig. S4. EDS-mapping of BP7003 before and after adsorption
